# Quercetin Increases Expression of Membrane-TRAIL in Glioblastoma Cells Resulting in Apoptosis

**DOI:** 10.3390/cancers17193197

**Published:** 2025-09-30

**Authors:** Erin M. Thorpe, Gaëlle Muller-Greven, Jamila Hirbawi, Candece L. Gladson, Michael Kalafatis

**Affiliations:** 1 Department of Chemistry, Cleveland State University, Science and Research Center, Cleveland, OH 44115, USA; e.m.thorpe@vikes.csuohio.edu (E.M.T.); j.hirbawi@csuohio.edu (J.H.); 2 Center for Gene Regulation in Health and Disease (GRHD), Cleveland State University, Cleveland, OH 44115, USA; 3 Department of Cancer Science, Cleveland Clinic, Cleveland, OH 44195, USA; mullerg@ccf.org (G.M.-G.); gladsoc@ccf.org (C.L.G.); 4 Brain Tumor and Neuro-Oncology Center, Cleveland Clinic, Cleveland, OH 44195, USA

**Keywords:** apoptosis, intrinsic, extrinsic, glioblastoma, caspase, quercetin, TRAIL

## Abstract

Background/Objectives: Glioblastoma isocitrate dehydrogenase (IDH)-wild type (GBM) belongs to a deadly class of cancers with a limited number of effective therapies and a dismal prognosis. Quercetin is a natural flavonoid with proven anti-cancer effects. This study aimed to assess the effect of quercetin on recombinant human tumor necrosis factor-related apoptosis-inducing ligand (rhTRAIL)-mediated apoptosis in various GBM cells and control astrocytes. Methods: Two astrocyte cell lines and three GBM cell lines, M059K, T98G, and A172, were treated with quercetin (±rhTRAIL), and the results were evaluated by Western blotting, confocal microscopy, and flow cytometry analyses. Results: Quercetin alone did not induce apoptosis in normal astrocytes. Surprisingly, quercetin alone induced apoptosis in all GBM cell lines through both the intrinsic and extrinsic pathways of apoptosis in a TRAIL-dependent manner. M059K were the most sensitive to quercetin-induced apoptosis, followed by T98G and A172. We determined that GBM cells possess endogenous membrane-TRAIL, and that quercetin, in a time- and concentration-dependent manner, increased the trafficking of membrane-TRAIL to the cell surface. Conclusions: We demonstrate that quercetin alone induces apoptosis in GBM cell lines by facilitating endogenous membrane-TRAIL trafficking to the cell surface, where it can interact with death receptors already present on the surface of neighboring cancer cells, resulting in cell death. This unexpected finding may prove to be invaluable for potential future treatment of patients with GBM, since administration of quercetin can cause increased trafficking of membrane-TRAIL to the cell surface, inducing cancer cell apoptosis without affecting neighboring normal cells.

## Simple Summary

Glioblastoma (GBM) is a highly aggressive type of brain cancer with limited treatment options and typically a terminal prognosis. The immune system possesses a protein that is called tumor necrosis factor-related apoptosis-inducing ligand (TRAIL) that naturally can destroy cancer cells. Because our aim was to explore a natural course of treatment for GBM, we assessed the ability of the natural compound quercetin to induce cell death in various GBM cell lines and non-cancerous normal brain cells. In the absence of added TRAIL, quercetin did not induce cell death in normal brain cells, but quercetin alone induced cell death in all GBM cell lines, in a TRAIL-dependent manner. This led to the novel observation that GBM cells already have TRAIL inside them. We further demonstrated that quercetin increases TRAIL trafficking from inside the cell to its surface, resulting in the destruction of the neighboring cancer cells. Thus, hypothetically, in the presence of quercetin, an entire brain tumor could be treated without any harmful effects on bordering normal brain cells. This novel and unexpected mechanism of GBM self-destruction in the presence of quercetin, combined with quercetin’s inability to harm normal brain cells, highlights its potential as a promising therapeutic for GBM.

## 1. Introduction

Glioblastoma isocitrate dehydrogenase (IDH)-wild type (GBM) is one of the most aggressive brain cancers with a dismal prognosis of an 18–21 month survival rate after initial diagnosis [1]. According to the National Cancer Institute, it accounts for 15% of adult brain tumors with no long-term cures. Only palliative measures, such as surgical removal of part, or all, of the tumor, along with chemotherapeutic and radiation therapies, are currently available. Many efforts to combat GBM have involved the use of chemotherapeutic agents, such as temozolomide (TMZ), to increase survival times, but resistance inevitably develops [2]. Sadly, these existing options still only extend a patient’s life by approximately six months, and the negative side effects associated with these treatments can greatly impact the overall quality of life [3]. Therefore, it is imperative to develop innovative therapies that increase patient survival rates without adverse side effects.

Apoptosis is the body’s innate way of disposing of damaged or excess cells during development and aging. The process of apoptosis is divided into two distinct pathways: the intrinsic mitochondrial-mediated pathway and the extrinsic death-receptor mediated pathway [4,5,6,7,8]. Tumor necrosis factor-related apoptosis-inducing ligand (TRAIL) is an endogenous membrane-associated cytokine that can selectively eradicate cancer cells by activating the body’s natural mechanism of apoptosis [5,6]. The recombinant human form of TRAIL (rhTRAIL) is a soluble version of the protein that has also been found to induce apoptosis in cancer cells without negatively impacting normal non-transformed cells [6,9]. Membrane-TRAIL (and rhTRAIL) can bind to five known receptors, but apoptosis is only induced upon binding to membrane death receptors (DRs), DR4 and DR5, with full-length intracellular death domains [5,7]. TRAIL binding to DR4 or DR5 causes the adaptor protein FADD and pro-caspase-8 to be recruited to the functional death domain of the receptor, leading to the cleavage and activation of initiator caspase-8. Active caspase-8 cleaves downstream executioner caspase-3, caspase-6, or caspase-7, which can then cleave and inactivate the DNA repair enzyme, PARP. PARP cleavage ultimately leads to the characteristic events of apoptosis, including chromatin condensation, nuclear fragmentation, cell membrane blebbing, and the formation of apoptotic bodies [4,5,6,7,8]. Alternatively, the intrinsic pathway of apoptosis is initiated by DNA damage, which signals p53 to transcriptionally upregulate pro-apoptotic proteins that lead to mitochondrial outer membrane permeabilization, and the subsequent release of cytochrome c. Cytosolic cytochrome c interacts with APAF1 and pro-caspase-9 to form the apoptosome signaling complex. This complex activates caspase-9, which can then cleave the executioner caspases and induce apoptosis in the same fashion as the extrinsic pathway. Anti-apoptotic proteins prevent mitochondrial outer membrane permeabilization, and inhibitors of apoptosis block caspase-9 from cleaving executioner caspases. Although separate, both pathways of apoptosis converge at the executionary phase, and the extrinsic pathway can activate the intrinsic pathway in a p53-independent manner through caspase-8-mediated cleavage of the pro-apoptotic protein BID to truncated BID, which enters the mitochondria and leads to the release of cytochrome c [4,5,6,7,8].

Various studies have been performed to clarify the effects of TRAIL therapy on malignant gliomas, leading to the finding that some brain-derived glioma cell lines are sensitive to TRAIL while others are resistant or develop resistance over time [1]. Multiple causes for TRAIL resistance have been proposed [10], but the variety of underlying factors responsible is still largely unknown. Fortunately, TRAIL resistance can often be overcome through cotreatment with other compounds, including those found in nature [1,6,11,12,13,14,15,16].

A recent review highlighted the substantial potential of bioactive phytochemicals for GBM prevention and therapy, supported by preclinical evidence across multiple tumorigenic pathways [17]. Quercetin, a dietary flavonoid abundant in plants, grains, fruits, and vegetables, exhibits multi-target anti-tumor activity in GBM cells by inducing apoptosis and inhibiting angiogenesis, proliferation, migration, and invasion [17]. Previous research in our laboratory showed that quercetin significantly improved rhTRAIL-mediated apoptosis in breast cancer [11] and malignant melanoma cell lines [14]. In several glioma cell lines, the ability of quercetin to enhance TRAIL-mediated apoptosis through the degradation of survivin, a key member of the inhibitors of apoptosis family, was demonstrated [15]. Importantly, quercetin exhibits potent anti-cancer activity on a wide array of cancer types with practically nonexistent adverse side effects on healthy cells [18,19]. The objective of this research was to explore the effects of quercetin and rhTRAIL in various GBM cell lines and normal non-transformed astrocytes.

## 2. Materials and Methods

### 2.1. Drugs and Chemicals

rhTRAIL was synthesized according to previously described procedures [20,21,22]. Briefly, a strain-optimized cDNA construct was used to produce rhTRAIL in *E. coli* through a high-density cell culture and feeding strategy. After induction for 22 h, the cell paste was collected and rhTRAIL was purified by sequential FPLC, followed by verification through SDS-PAGE and Coomassie Blue staining after each step. Ultimately, the final product was found to exceed 99% purity by HPLC. Quercetin dihydrate (Thermo Fisher Scientific, Pittsburgh, PA, USA) and staurosporine (Thermo) were solubilized in dimethyl sulfoxide (DMSO) (Sigma-Aldrich, St. Louis, MO, USA).

### 2.2. Cell Culture

The human GBM cell line M059K (American Type Culture Collection (ATCC), Manassas, VA, USA, Cat#: CRL-2365, RRID: CVCL_0401) was maintained in DMEM-F12 (Cleveland Clinic Cell Services Media Core, Cleveland, OH, USA) supplemented with 10% fetal bovine serum (FBS; Gibco, Gaithersburg, MD, USA), 1% antibiotic–antimycotic (Gibco), and 1% minimal essential media non-essential amino acids (Gibco). The human GBM cell lines A172 (ATCC, Cat#: CRL-1620, RRID: CVCL_0131) and T98G (ATCC, Cat#: CRL-1690, RRID: CVCL_0556) were maintained in high glucose DMEM (Cleveland Clinic) containing 10% FBS and 1% antibiotic–antimycotic. Two different primary non-tumorigenic human astrocyte cell isolates, denoted as Astro-1 (Cell Systems Corporation, Kirkland, WA, USA, Cat#: ACBRI-371) and Astro-2 (Celprogen, Torrance, CA, USA, SKU: 36058-01), were maintained in high glucose DMEM supplemented with 10% FBS, 1% antibiotic–antimycotic, and 0.2% Bac-off (Cell Systems). Cells were incubated at 90% humidity with 5% CO_2_ at 37 °C.

### 2.3. Western Blot Analysis for Markers of Apoptosis

Cells were plated and allowed to adhere for 24 h prior to treatment. After 24, 48, or 72 h of treatment the total cell lysates were prepared using RIPA lysis buffer (Sigma) containing 150 mM sodium chloride, 1.0% Triton X-100, 0.5% sodium deoxycholate, 0.1% SDS and 50 mM Tris, at pH 8.0 plus a 1X cocktail of protease inhibitors (Protease Inhibitor Cocktail Set I, Calbiochem (Sigma)). Cells were lysed for 30 min on ice, centrifuged at 10,000 rpm for 10 min at 4 °C, and then the supernatant containing the solubilized proteins was collected. Protein concentrations were calculated using the Pierce BCA assay with a bovine serum albumin standard curve (Thermo). Whole cell lysates containing 35 µg of total protein were mixed with 4X Laemmli sample buffer (250 mM Tris-HCl, 8% SDS, 40% glycerol, 8% βME, and 0.02% Bromophenol blue), heated at 100 °C for 5 min, and loaded onto 12% or 15% polyacrylamide gels. Proteins were separated by SDS-PAGE, transferred to a polyvinylidene fluoride (PVDF) membrane (Sigma) which was blocked with 5% non-fat milk for 1 h at room temperature and then incubated overnight at 4 °C with diluted primary antibodies to blot for expression of PARP (Cell Signaling Technology (CST), Danvers, MA, USA, Cat# 9542, RRID: AB_2160739), caspase-8 (CST, Cat# 9746, RRID: AB_2275120 and Cat# 9496, RRID: AB_561381), caspase-9 (Thermo, Cat# MA1-16842, RRID: AB_568482), caspase-3 (CST, Cat# 9668, RRID: AB_2069870 and Cat# 9661, RRID: AB_2341188), and caspase-7 (CST, Cat# 9492, RRID: AB_2228313). Following incubation, the membrane was washed with TBS-Tween (0.15 M NaCl, 0.02 M Tris, and 0.3% Tween-20 with pH 7.4) and then incubated with the appropriate HRP-conjugated secondary antibody (Bio-Rad Laboratories, Hercules, CA, USA, Cat# 1706516, RRID: AB_2921252 or Cat# 1706515, RRID: AB_11125142) for 1 h at room temperature. The membrane was washed again, and the proteins were visualized through chemiluminescence using WesternBright^TM^ ECL substrate spray (Advansta, San Jose, CA, USA) followed by exposure on HyBlot CL Autoradiography Film (Denville Scientific, Holliston, MA, USA). Each blot was reprobed with β-actin (Sigma, Cat# A3854, RRID: AB_262011) to confirm equal loading, and a representative β-actin blot is shown for each set of experiments.

### 2.4. FITC-Annexin V Flow Cytometry Apoptosis Assay

Apoptotic cells were identified using FITC-Annexin V Apoptosis Detection Kit I (Becton, Dickinson and Company (BD) Biosciences, San Jose, CA, USA, Cat# 556547, RRID: AB_2869082). After 72 h of treatment, cells were collected, counted, and 1 × 10^5^ cells were placed in each flow tube. Cells were spun at 1250 rpm for 5 min and washed twice with PBS. Then the cells were resuspended in 100 μL of Annexin-V binding buffer containing 5 μL of FITC-Annexin V and allowed to incubate at room temperature for 20 min in the dark. Cells were washed with Annexin V binding buffer, centrifuged, and resuspended in Annexin-V binding buffer containing 5 μL of propidium iodide (PI). An unstained control and two single-color controls (FITC-Annexin V or PI only) were used to set up compensation and quadrants for flow cytometry. BDFACS Canto II with FACSDiva software version 6.1.3 firmware version 1.47 (RRID: SCR_001456) was used to analyze stained cells. Further analysis and visualization were completed using Flowjo software version 10.10.0 (Flowjo LLC, RRID: SCR_008520). Each experiment was performed in triplicate (*n* = 3) to obtain the average percent of apoptotic cells ± the standard error of the mean (SEM).

### 2.5. Flow Cytometry Analysis of Membrane DR Expression

After 72 h of treatment, cells were collected, counted, and 1 × 10^5^ cells were placed in each flow tube. Cells were spun at 1500 rpm for 5 min and washed three times with 1 mL of 1X flow cytometry staining buffer (R & D systems Inc., Minneapolis, MN, USA). Cells were resuspended in 100 μL of 1X flow cytometry staining buffer containing 5 μL of Human Seroblock (Bio-Rad) and allowed to incubate for 10 min at room temperature to reduce Fc-receptor-mediated binding. Unstained samples were kept for each treatment condition to assess autofluorescence and establish background noise. For the stained samples, 5 μL of either mouse anti-human DR4 PE (Thermo, Cat# 12-6644-42, RRID: AB_10852861), mouse anti-human DR5 PE (Thermo, Cat# 12-9908-42, RRID: AB_10668836), or the mouse IgG1 kappa PE isotype control (Thermo, Cat# 12-4714-82, RRID: AB_470060) were added directly into their respective tubes. All samples were mixed by gentle pipetting and allowed to incubate at room temperature for 20 min in the dark. Following staining, cells were washed three times with 1 mL of staining buffer. After the last wash, the cells were resuspended in 300 µL staining buffer, filtered, and subjected to flow cytometry analysis. Cells were analyzed on the BDFACS Canto II using FACSDiva software, and then Flowjo software was used to further evaluate and visualize the results. Stained samples were analyzed in triplicate (*n* = 3) to calculate the SEM. For each sample, 30,000 cells were analyzed, live cell populations were identified, doublets were removed, and then the unstained cells were used to establish PE gating parameters. The normalized blank-subtracted PE+ live single cell percentages (membrane DR positive cells) were calculated for each treatment condition using the following equation:

$$Membrane\;DR\;positive\;cells=\frac{Average\;stained\;{PE}_{+}\;cells}{Average\;stained\;{PE}_{Total}\;cells}-\frac{Unstained\;{PE}_{+}\;cells}{Unstained\;{PE}_{Total}\;cells}\times 100$$

### 2.6. Western Blot Analysis for Membrane-TRAIL Expression

Untreated cells were collected at 80% confluency, centrifuged at 1000 rpm for 5 min, washed twice with PBS, and resuspended in RIPA containing protease inhibitors. Cells were lysed as described above, the supernatant was collected, total protein concentration was determined through BCA, and then aliquots of 50 or 100 µg of total protein were used for analysis. A set of rhTRAIL standards (0.5 and 0.25 ng) diluted in 1X TBS was used as positive controls. rhTRAIL consists only of the active C-terminal domain with a molecular weight (MW) of 20 kilodaltons (kDa) [22]. A lysate from HEK293T cells with transient human membrane-TRAIL over-expression and a whole cell HEK293T lysate without transfection were used as positive and negative controls, respectively (Origene Technologies Inc., Rockville, MD, USA, NM_003810, SKU: LC418411, Lot# RK035FO). The HEK293T lysates were prepared according to the manufacturer’s instructions with the provided 2X SDS Sample Buffer (4% SDS, 125 mM Tris-HCl, pH 6.8, 10% Glycerol, 0.002% Bromophenol blue, 100 mM DTT), and then further dilutions of HEK293T lysates were carried out in 4X Laemmli sample buffer. Membrane-TRAIL in the HEK293T cells contains a c-Myc/DDK tag, changing the observed MW of membrane-TRAIL from the expected 32.5 kDa [23] to 36 kDa. All samples were mixed with 4X Laemmli sample buffer, heated at 100 °C for 10 min, and then separated on a 15% SDS-polyacrylamide gel. Proteins were transferred to PVDF, blocked for 1 h at room temperature with 2.5% milk, and incubated overnight at 4 °C with a 0.5 mg/mL mouse anti-human TRAIL (SouthernBiotech, Birmingham, AL, USA, Cat# 11040-01, RRID: AB_2794597) at a dilution of 1:334 (1.5 µg/mL) in 2.5% milk. After incubation with the primary antibody, the membrane was washed with TBS-Tween and incubated with an HRP-conjugated anti-mouse secondary antibody (SouthernBiotech, Cat# 1030-05, RRID: AB_2619742) for 1.5 h at room temperature. The membrane was washed, and then the proteins were visualized with chemiluminescence, followed by exposure on X-ray film.

### 2.7. Flow Cytometry Analysis of Membrane-TRAIL Expression

Following 24, 48, or 72 h of treatment, cells were collected, counted, placed in flow tubes, spun, washed, Fc blocked, stained, washed, filtered, and subjected to flow analysis as previously described in the membrane DR analysis. For the stained samples, 5 μL of either mouse anti-human TRAIL Alexa Fluor 488 (R & D systems, Cat# FAB687G, RRID: AB_10972103) or the mouse IgG1 Alexa Fluor 488 isotype control (R & D Systems, Cat# IC002G, RRID: AB_10718385) were added directly into their respective tubes. BDFACS Canto II, using FACSDiva software and Flowjo software, was used to analyze cells and visualize the results. The normalized blank-subtracted *FITC*_+_ live single cell percentages (membrane-TRAIL positive cells) were calculated for each treatment condition using the following equation:

$$Membrane\;TRAIL\;positive\;cells=\frac{Average\;stained\;{FITC}_{+}\;cells}{Average\;stained\;{FITC}_{Total}\;cells}-\frac{Unstained\;{FITC}_{+}\;cells}{Unstained\;{FITC}_{Total}\;cells}\times 100$$

### 2.8. Double-Label Immunofluorescence

Double-label immunofluorescence was performed according to previously described methods [24,25]. Astro-2 and M059K cells were plated on separate glass chamber slides coated with 20 μg/mL laminin in their respective mediums for 2 days. Cells were then treated with quercetin or the DMSO control for 24 h, fixed, reacted with 5 µg/mL wheat germ agglutinin (WGA) Alexa Fluor 647 (Thermo, Cat# W32466) for 10 min, washed, and then incubated with 10 µg/mL goat anti-human TRAIL (R & D Systems, Cat# AF375, RRID: AB_355334) overnight at 4 °C. The next day, cells were reacted with 5 µg/mL donkey anti-goat IgG Alexa Fluor 488 (Thermo, Cat# A-11055, RRID: AB_2534102) for 1 h at 22 °C, stained with DAPI nuclear stain, and then cover-slipped. Cells were imaged on a Leica confocal microscope and analyzed using ImageJ software version 1.53t (RRID: SCR_003070, supplied through the NIH).

### 2.9. Statistical Analysis

For the flow cytometry analyses of each cell line, separate treatment concentrations were compared individually to the DMSO control, and additionally, when applicable, single treatments were compared to combination treatments, using Welch’s *t*-test (two-sample, two-tailed, unequal variance). For pairwise comparisons between conditions in the immunofluorescence experiments, non-parametric statistical analyses were performed using the Wilcoxon Rank Sum test. Statistical significance was defined as a *p*-value less than 0.05. In histograms, *p* < 0.05 was depicted as *, *p* < 0.01 as **, *p* < 0.001 as ***, and *p* < 0.0001 as ****.

## 3. Results

### 3.1. Quercetin Alone Does Not Induce Apoptosis in Normal Astrocytes

**Figure 1:**
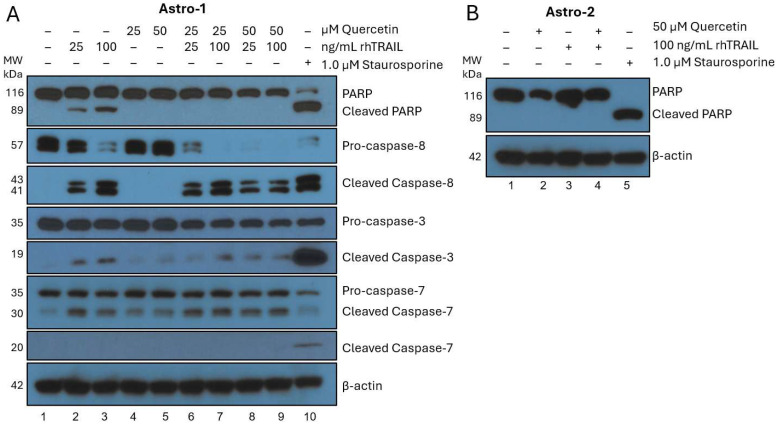
Western blot analysis of quercetin and rhTRAIL cotreatment in non-tumorigenic primary human astrocytes. Cells were collected, lysed, and normalized to protein after 72-h of treatment. Proteins were separated using gel electrophoresis and transferred to PVDF, then membranes were probed with various antibodies for apoptosis as detailed in the methods Section 2.3. Each blot was probed for β-actin to confirm equal loading. A representative β-actin is depicted for each set of experiments. Molecular weight (MW) markers from pre-stained ladder are shown on the left in kilodaltons (kDa). (**A**) Astro-1 were treated with quercetin (0, 25, or 50 µM) plus or minus rhTRAIL (25 or 100 ng/mL) or with 1.0 µM staurosporine which was added 24-h prior to collection. Quercetin did not induce apoptosis but rhTRAIL did show minor apoptotic induction which increased corresponding to concentration. Interestingly, when quercetin was added in combination with rhTRAIL, PARP cleavage was mitigated. The observed protective effect of quercetin may in-part be through a reduction of cleaved caspase-8 and caspase-3. As expected, staurosporine induced significant apoptosis. (**B**) Astro-2 were treated with quercetin (0 or 50 µM) with or without rhTRAIL (100 ng/mL) or with 1.0 µM staurosporine added 24-h prior to collection. No treatment with quercetin or rhTRAIL showed apoptosis but staurosporine showed substantial PARP cleavage. PARP: poly (ADP-ribose) polymerase; PVDF: polyvinylidene fluoride; rhTRAIL: recombinant human tumor necrosis factor-related apoptosis inducing ligand.

To examine the effects of quercetin and TRAIL on healthy cells, commercially available astrocytes (two distinct control cell lines, Astro-1 and Astro-2, from different vendors) were treated with quercetin plus or minus rhTRAIL (Figure 1, panels A and B). A control plate was spiked with 1.0 µM staurosporine 24 h prior to collecting to serve as a positive control, as this compound has been shown to induce apoptosis in astrocytes [26]. Astro-1 treated with DMSO or quercetin alone did not exhibit cleavage of PARP or pro-caspases, indicating that quercetin alone has no apoptotic effect on these normal brain cells (Figure 1A, lanes 1, 4, and 5). rhTRAIL alone caused a slight amount of apoptosis in the Astro-1 cell line through cleavage of PARP, pro-caspase-8, pro-caspase-3, and pro-caspase-7, which increased depending on rhTRAIL concentration (Figure 1A, lanes 2 and 3). A non-canonical cleavage fragment of pro-caspase-7 at 30 kDa was also observed, which has been recently detected both *in vitro* and *in vivo*, in normal and cancerous cells. Unlike the 20 kDa fragment that is associated with apoptosis and seen with staurosporine treatment, the 30 kDa fragment has been linked to a cytoprotective autophagic response that can occur following non-lethal stress [27]. This adaptive alternative cleavage still needs to be further investigated to fully characterize its physiological roles.

Interestingly, PARP cleavage was not observed in the combination treatment of quercetin plus rhTRAIL, despite cleavage of pro-caspase-8 (Figure 1A, lanes 6–9). Astro-2 (different commercially available astrocyte cell line control) did not show any PARP cleavage in the presence of 50 µM quercetin, 100 ng/mL rhTRAIL, or the cotreatment (Figure 1B, lanes 2–4). As expected, staurosporine treatment of Astro-1 and Astro-2 resulted in near complete (Astro-1) and complete (Astro-2) cleavage of PARP (Figure 1A, lane 10; Figure 1B, lane 5). Detailed analysis of staurosporine treatment in Astro-1 cells showed cleavage of pro-caspase-8, along with strong cleavage of pro-caspase-3 (Figure 1A, lane 10). Taken together, this data highlights the safety of quercetin, as it does not induce apoptosis in either astrocyte cell line. In contrast, rhTRAIL may trigger apoptosis in some astrocytes and not others, suggesting an inherent difference between these control cell lines. Unexpectedly, our data also demonstrates that quercetin has a protective effect on the rhTRAIL-sensitive astrocyte cells, shielding them from apoptosis despite caspase-8 activation. Our findings highlight the substantial variability among commercially available astrocyte cell lines, emphasizing the inherent heterogeneity of these normal cells.

### 3.2. Treatment of Cancer Cells with Quercetin Alone Induces Cell Death Through Both the Intrinsic and Extrinsic Pathways of Apoptosis

The effect of quercetin and rhTRAIL on three different GBM cancer cell lines, M059K, T98G, and A172, was assessed. To better understand the impact of incubation time on quercetin’s effectiveness, we analyzed the apoptosis profile of M059K cells after treatment with varying concentrations of quercetin for 24 and 48 h. Surprisingly, after a 24-h incubation with 10 µM quercetin alone, a modest level of extrinsic pathway-mediated apoptosis was detected, as evidenced by cleavage of PARP, caspase-8, caspase-3, and caspase-7 (Figure S1, panel A). Incubation with 10 µM quercetin for 48 h showed a slight increase in apoptosis compared to the 24-h treatment, with nearly identical patterns of cleavage (Figure S1, panel B). Significant apoptosis was not observed at these earlier times, indicating that the effect of quercetin treatment is dependent on time (present data) as well as the concentrations of quercetin and rhTRAIL, as we have previously shown in other cancer cell lines [9,11,14].

**Figure 2:**
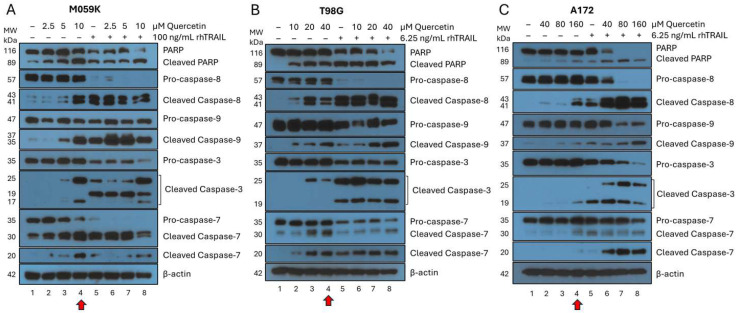
Western blot analysis of apoptosis in GBM cells following treatment with quercetin and rhTRAIL. After 72 h of treatment, cells were collected, lysed, and normalized to protein. Following gel electrophoresis and transfer to PVDF, membranes were probed with the appropriate antibodies for apoptosis as detailed in the methods section. Each blot was probed for β-actin to confirm equal loading, and a representative β-actin is shown for each experimental set. Molecular weight (MW) markers from a pre-stained ladder are shown on the left in kilodaltons (kDa). Quercetin improved rhTRAIL-induced apoptosis in each GBM cell line, which was shown through enhanced activation of the caspase cascade and PARP cleavage. (**A**) M059K cells were treated with quercetin (0, 2.5, 5, or 10 µM) plus or minus rhTRAIL (100 ng/mL). (**B**) T98G were treated with quercetin (0, 10, 20, or 40 µM) with or without rhTRAIL (6.25 ng/mL). (**C**) A172 were treated with quercetin (0, 40, 80, or 160 µM), including or not including rhTRAIL (6.25 ng/mL). The red arrow at the bottom of each panel indicates the results obtained when cells were incubated with the highest concentration of quercetin alone in the absence of exogenously added rhTRAIL. GBM: glioblastoma isocitrate dehydrogenase (IDH)-wild type; PARP: poly (ADP-ribose) polymerase; PVDF: polyvinylidene fluoride; rhTRAIL: recombinant human tumor necrosis factor-related apoptosis-inducing ligand.

M059K cells were treated with quercetin plus or minus rhTRAIL for 72 h (Figure 2, panel A). Surprisingly, the incubation of M059K with increasing concentrations of quercetin alone resulted in increased PARP cleavage (Figure 2A, lanes 2–4). In the presence of 10 μM quercetin alone, M059K cells underwent apoptosis through both the intrinsic and extrinsic pathways of apoptosis as visualized by the activation of caspase-9 and caspase-8, respectively (Figure 2A, lane 4, red arrow). rhTRAIL alone (100 ng/mL) also induced PARP cleavage above the basal level (Figure 2A, lane 5), suggesting that M059K cells are somewhat sensitive to rhTRAIL alone. Near-complete PARP cleavage was seen with the cotreatment of rhTRAIL in the presence of the highest concentration of quercetin (10 µM), indicating that quercetin enhances the sensitivity of M059K to rhTRAIL-induced apoptosis (Figure 2A, lane 8). It is noteworthy that in the presence of rhTRAIL alone, virtually complete cleavage of pro-caspase-8 was observed, which is consistent with the activation of the extrinsic pathway of apoptosis (Figure 2A, lane 5). rhTRAIL alone was also able to activate the intrinsic pathway of apoptosis, marked by pro-caspase-9 activation, and the combination with quercetin seemed to enhance this cleavage, even at the lowest quercetin concentrations (2.5 and 5 µM) (Figure 2A, lanes 6–8). Cleavage of executioner caspase-3 was seen at 10 µM quercetin alone and in all rhTRAIL-treated cells, but the cleavage pattern varied based on the concentrations of quercetin and rhTRAIL (Figure 2A, lanes 4–8). Near complete cleavage of pro-caspase-7 was observed in single treatments of 10 μM quercetin or 100 ng/mL rhTRAIL alone, and complete cleavage was seen in all combination treatments (Figure 2A, lanes 4–8). Interestingly, non-canonical cleavage of pro-caspase-7 at 30 kDa seemed to increase in all treated conditions, except the highest combination treatment, while prominent cleavage of pro-caspase-7 into the 20 kDa band, associated with apoptosis, was mostly observed in treatments with 10 µM quercetin (Figure 2A, lanes 4 and 8). In conclusion, this data demonstrates that quercetin alone and rhTRAIL alone induce apoptosis in M059K cells through a similar mechanism (Figure 2A, lanes 4 and 5).

T98G cells were treated with quercetin with or without rhTRAIL for 72 h (Figure 2, panel B). Similar to the data obtained with M059K, an increase in PARP cleavage was seen in the presence of 20 or 40 µM quercetin alone (Figure 2B, lanes 3 and 4). The amount of PARP cleavage with rhTRAIL alone (6.25 ng/mL) was comparable to that of the higher concentrations of quercetin alone (Figure 2B, lanes 4 and 5). Almost complete cleavage of PARP was observed when cells were incubated with the combination of rhTRAIL and 40 µM quercetin (Figure 2B, lane 8). Treatment with quercetin alone activated both the extrinsic and intrinsic apoptotic pathways, as indicated by the cleavage of pro-caspase-8 and pro-caspase-9, respectively (Figure 2B, lanes 2–4). By itself, rhTRAIL (6.25 ng/mL) induced complete cleavage of pro-caspase-8 and significant cleavage of pro-caspase-9, indicating that these cells are fairly rhTRAIL sensitive, with apoptosis proceeding through both pathways of apoptosis (Figure 2B, lane 5). Treatment of the cells with rhTRAIL in combination with 40 µM quercetin led to a substantial increase in cleavage of pro-caspase-9 (Figure 2B, lane 8). Quercetin alone above 20 µM showed activation of pro-caspase-3 and pro-caspase-7 (Figure 2B, lanes 3 and 4), and all treatments containing rhTRAIL displayed optimal cleavage of these executioner caspases (Figure 2B, lanes 6–8). Interestingly, the non-canonical 30 kDa cleavage of pro-caspase-7 was only notable in the treatments with 20 and 40 µM quercetin alone (Figure 2B, lanes 3 and 4). These results verify our findings with M059K and demonstrate that treatments with quercetin or rhTRAIL alone induce apoptosis in T98G cells through a similar mechanism as observed with M059K cells. There is a noticeable difference in sensitivity to quercetin and rhTRAIL between the two cell lines, with M059K being more sensitive to quercetin and less to rhTRAIL when compared to T98G.

A172 cells were treated with quercetin plus or minus rhTRAIL for 72 h (Figure 2, panel C). A172 cells were relatively resistant to quercetin treatment, with only minimal PARP cleavage seen at 160 µM (Figure 2C, lane 4, red arrow). However, the pattern seen with M059K and T98G continued as apoptosis in A172 cells occurred through both the extrinsic and intrinsic pathways, evidenced by cleavage of pro-caspase-8 and pro-caspase-9, respectively (Figure 2C, lane 4, red arrow). Cleavage of pro-caspase-3 was observed with 160 µM quercetin, and all treatments containing rhTRAIL, but the 25 kDa cleavage band was only seen in the cotreatments (Figure 2C, lanes 4–8). Interestingly, cleavage of pro-caspase-7 followed a similar trend with the non-canonical 30 kDa fragment appearing most prominently in 160 µM quercetin, and all treatments containing rhTRAIL, while the 20 kDa apoptosis-associated fragment was strongest in the cotreatments (Figure 2C, lanes 4–8). rhTRAIL alone showed a nearly identical cleavage profile to the 160 µM quercetin solo treatment with comparable intensity, with the only noticeable difference being an increase in cleaved pro-caspase-3 (Figure 2C, lanes 4 and 5). The combination treatments with higher concentrations of quercetin (80 and 160 µM) were significantly more effective, evidenced by complete PARP cleavage and enhanced activation of both caspase-8 and caspase-9 (Figure 2C, lanes 7 and 8). Overall, the combined data clearly demonstrate that the three GBM cell lines have varying sensitivities to rhTRAIL. Surprisingly, our data also established that GBM cell lines are sensitive to quercetin alone, albeit some more than others. Unexpectedly, the data suggest that quercetin alone, which can cross the blood–brain barrier (BBB) and has been detected in brain tissue in rats following oral administration [28], induces apoptosis in all three GBM cell lines through the intrinsic and extrinsic pathways of apoptosis, mimicking the mechanism employed by rhTRAIL alone.

### 3.3. Cotreatment Substantially Increases the Percentage of Cells Undergoing Apoptosis in All GBM Cell Lines

**Figure 3:**
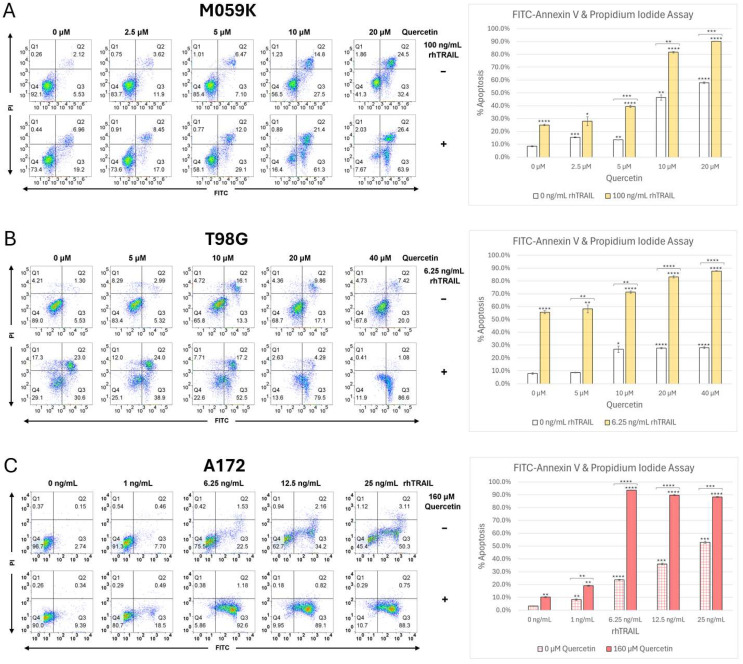
Quercetin enhances rhTRAIL-mediated apoptosis. The GBM cells (**A**) M059K, (**B**) T98G, and (**C**) A172 were treated with quercetin alone, rhTRAIL, or the combination of the two for 72 h. Cells were stained with FITC-Annexin V and PI, followed by flow cytometry analysis. The lower left quadrant (Q4) shows viable cells (Annexin V−/PI−), the lower right quadrant (Q3) shows early apoptotic cells (Annexin V+/PI−), the upper right quadrant (Q2) shows late apoptotic cells (Annexin V+/PI+), and the upper left quadrant (Q1) shows necrotic cells (Annexin V+/PI+). Representative images are displayed. The bar graphs contain the mean total percentage of total apoptotic cells (early and late apoptosis) ± SEM from each experiment performed in triplicate (*n* = 3). Each treatment was compared to the control, and single treatments were compared to combination treatments using Welch’s *t*-test (two-sample, two-tailed, unequal variance) to evaluate significance with *p* < 0.05 depicted as *, *p* < 0.01 as **, *p* < 0.001 as ***, and *p* < 0.0001 as ****. FITC: fluorescein isothiocyanate; GBM: glioblastoma isocitrate dehydrogenase (IDH)-wild type; PI: propidium iodide; SEM: standard error of the mean.

Flow cytometry analysis was performed on M059K, T98G, and A172 to further assess the apoptotic cell death that was previously observed in these GBM cell lines following quercetin and rhTRAIL treatment. This data shows that M059K cells treated with increasing concentrations of quercetin, rhTRAIL, or the combination have significant levels of apoptosis compared to the DMSO-treated control cells (Figure 3, panel A). Unexpectedly, and consistent with our Western blot results showing caspase activation (Figure 2A), M059K cells treated with quercetin alone induced significant apoptosis. The percentage of total apoptotic cells shifted from the basal level of 8% to 46% and 58%, in the presence of 10 and 20 μM quercetin alone, respectively (Figure 3A, histogram, white bars). M059K were slightly sensitive to 100 ng/mL rhTRAIL treatment alone with 25% of cells undergoing apoptosis (Figure 3A, histogram, yellow bar). Approximately 90% of M059K cells treated with rhTRAIL in the presence of 20 μM quercetin underwent apoptosis (Figure 3A, histogram, yellow bar). This data clearly demonstrates that quercetin alone can induce apoptosis at levels comparable to rhTRAIL alone; however, the combined treatment is more efficient.

Flow cytometry analysis was used to measure apoptosis in T98G cells (Figure 3, panel B). Quercetin treatment alone only induced significant apoptosis at concentrations of 10 µM and above (Figure 3B, histogram, white bars). However, notable differences were observed in the number of cells undergoing early and late apoptosis, with the highest amount of late apoptotic cells seen at 10 µM and the greatest percentage of early apoptotic cells detected at 40 µM (Figure 3B, flow data). T98G cells were very sensitive to rhTRAIL treatment alone, with the number of apoptotic cells increasing from the base level of 8% to 56% in the presence of 6.25 ng/mL rhTRAIL (Figure 3B, histogram, yellow bar) (16-fold less than the 100 ng/mL concentration required to produce a similar effect in the M059K cell line). Treatment of T98G cells with rhTRAIL plus 20 and 40 µM quercetin increased their corresponding levels of apoptosis to 83% and 88%, indicating that the cotreatment was significantly more effective than either treatment alone (Figure 3B, histogram, yellow bars). The combination of 5 µM quercetin with rhTRAIL showed the greatest number of T98G cells undergoing late apoptosis, which gradually decreased with increasing concentrations of quercetin. Simultaneously, the amount of early apoptotic cells steadily grew with increasing concentrations of quercetin (Figure 3B, flow data). This data confirmed our Western blot results with the T98G cells and demonstrated that quercetin alone could induce apoptosis at levels comparable to rhTRAIL alone, although the combined treatment was more efficient.

Finally, A172 cells were also treated for 72 h with rhTRAIL in the presence or absence of quercetin. A172 cells treated with increasing concentrations of rhTRAIL, quercetin, and the combination therapy all revealed significant levels of apoptosis compared to the DMSO-treated cell control (Figure 3, panel C). The singular treatments of 1.0 ng/mL rhTRAIL and 160 µM quercetin showed the least number of apoptotic cells. A steady rise in apoptosis was seen as rhTRAIL concentration increased, with 24% apoptosis seen at 6.25 ng/mL rhTRAIL, 36% at 12.5 ng/mL, and 53% at 25 ng/mL (Figure 3C, histogram, red grid pattern bars). Quercetin cotreatment increased apoptotic induction at every concentration of rhTRAIL, verifying the fact that the combination treatment was more effective than either treatment alone. Total apoptosis increased in the cotreatment from 8% with 160 µM quercetin alone to 88% with the addition of 25 ng/mL of rhTRAIL (Figure 3C, histogram, red bars). It is noteworthy that, compared to M059K and T98G, A172 cells are relatively resistant to apoptosis induced by quercetin alone, even at concentrations as high as 160 μM. Overall, these results confirm and expand on our previous findings with melanoma and breast cancer cells [11,14] and show for the first time that all three GBM cell lines undergo apoptosis in a TRAIL-like manner in the presence of quercetin alone, albeit with varying sensitivities.

### 3.4. Endogenous Membrane-TRAIL Protein Is Detected in GBM Cells and Astrocytes

**Figure 4:**
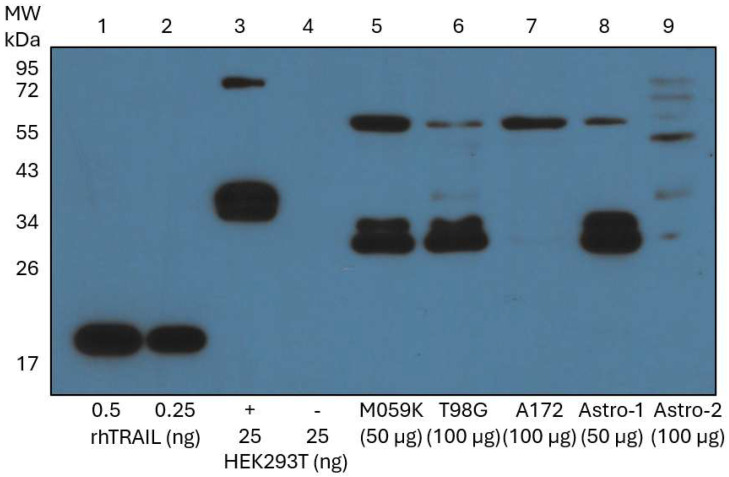
Endogenous TRAIL expression in brain-derived cells. Endogenous TRAIL expression in untreated whole cell lysates from the GBM cell lines and non-transformed human astrocytes was evaluated through Western blot analysis with a monoclonal mouse anti-human TRAIL antibody, as detailed in the methods section. A set of soluble rhTRAIL standards and an HEK293T transient membrane-TRAIL over-expression lysate were used as positive controls, and a whole cell HEK293T lysate was used as a negative control. Molecular weight (MW) markers from a pre-stained ladder are shown on the left in kilodaltons (kDa). The observed MW of soluble rhTRAIL is 20 kDa, soluble TRAIL is 24 kDa (due to N-terminal heterogeneity), HEK293T membrane-TRAIL with a c-Myc/DDK tag is 36 kDa (dimer is 72 kDa), membrane-TRAIL is 32.5 kDa (dimer is 65 kDa), and glycosylated membrane-TRAIL is 41 kDa. GBM: glioblastoma isocitrate dehydrogenase (IDH)-wild type; TRAIL: tumor necrosis factor-related apoptosis-inducing ligand.

We have shown thus far that quercetin alone induces apoptosis in all three GBM cell lines in a TRAIL-like manner through the activation of both caspase-8 and caspase-9, indicating the involvement of both the extrinsic and intrinsic pathways of apoptosis, respectively. Consequently, we hypothesized that GBM cells may contain endogenous TRAIL, and that incubation with quercetin increases the translocation of TRAIL to the cell membrane by a yet to be described mechanism. A Western blot analysis with a monoclonal antibody to TRAIL was used to ascertain whether untreated and unstimulated whole cell lysates from normal non-transformed astrocyte cells (Astro-1 and Astro-2) and from the GBM cell lines (M059K, T98G, and A172) expressed endogenous TRAIL (Figure 4). A set of soluble rhTRAIL protein standards (0.5 ng and 0.25 ng) with an approximate MW of 20 kDa [22] (Figure 4, lanes 1 and 2), together with an HEK293T transient membrane-TRAIL over-expression lysate, were used as positive controls (Figure 4, lane 3). The membrane-TRAIL in this lysate has a MW of 36 kDa due to a c-Myc/DDK tag. An HEK293T whole cell lysate was used as a negative control (Figure 4, lane 4) because kidney cells have been shown to express negligible levels of endogenous membrane-TRAIL under normal conditions [29] and have been used as negative controls for TRAIL in other experiments [30]. All positive controls migrated at their expected MWs, and the negative control did not show any bands. Taken together, these controls verified the ability of the primary antibody to bind and specifically recognize both soluble rhTRAIL and membrane-TRAIL. The MWs of soluble rhTRAIL, soluble TRAIL (heterogeneous N-terminus), full-length membrane TRAIL, and glycosylated membrane-TRAIL are 20, 24, 32.5, and 41 kDa, respectively [23]. A strong band corresponding to the molecular weight of membrane-TRAIL was observed in two glioblastoma cell lines (M059K and T98G), and one control astrocyte cell line (Astro-1 cells) (Figure 4, lanes 5, 6, and 8). The classic membrane-TRAIL in the A172 was below the limit of detection, but a prominent band was seen near 65 kDa that was also seen in the Astro-1, M059K, and T98G cells. This band may correspond to membrane-TRAIL dimers that resisted reduction, as suggested by the 72 kDa band observed in the HEK293T positive control, which is consistent with dimerization of c-Myc/DDK-tagged membrane-TRAIL. Astro-2 cells showed a distinct band pattern compared to all the other cell lines. Faint bands were observed for full-length membrane and glycosylated membrane-TRAIL, as well as various high molecular weight bands, with the strongest band seen near 48 kDa. No soluble TRAIL (24 kDa) was observed in any samples under our conditions. In summary, our combined data demonstrate that all cells studied herein express endogenous membrane-TRAIL to some degree without stimulation or treatment.

### 3.5. Quercetin Enhances Endogenous Cell Surface Membrane-TRAIL Expression in Cancer Cells but Not Astrocytes

Flow cytometry analysis was used to investigate the effect of quercetin on the cell surface expression of membrane-TRAIL in control astrocytes (Astro-1 and Astro-2), M059K, T98G, and A172 cells after 72 h of treatment. Additionally, cell surface expression of membrane-TRAIL was investigated in the M059K cells following treatment with quercetin after 24 and 48 h to examine the effect of quercetin incubation time on endogenous membrane-TRAIL release. The normalized blank-subtracted *FITC*_+_ live single cell percentages were calculated to determine the portion of membrane-TRAIL positive cells for each treatment condition. Histograms were produced to display the mean percentage of membrane-TRAIL positive cells ± SEM for each condition. Raw histogram data obtained from each analysis, with an overlay of unstained samples (black unfilled traces) and stained samples (filled and color-coded accordingly), were provided.

**Figure 5:**
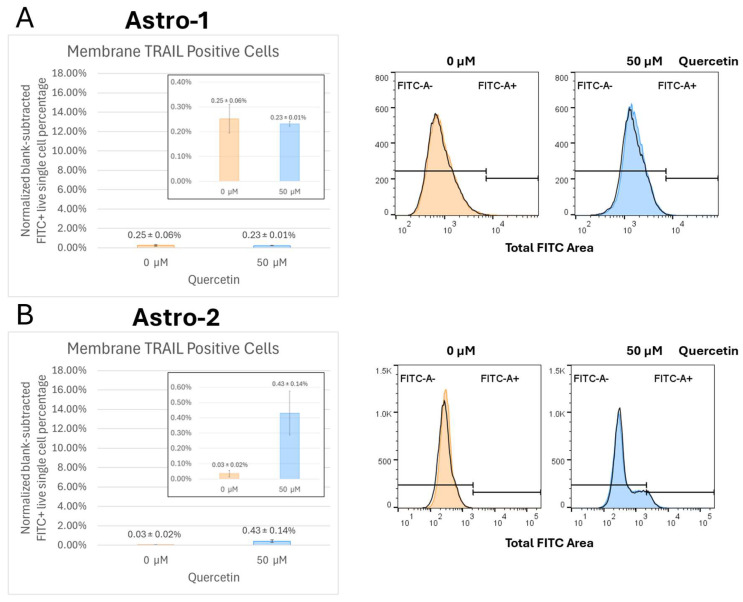
Expression of membrane-TRAIL on the surface of normal Astrocytes. (**A**) Astro-1 and (**B**) Astro-2 cells were treated with quercetin alone for 72 h. Cells were collected, washed, and Fc-receptors were blocked with Human Seroblock. Then the cells were stained with a mouse anti-human TRAIL Alexa Fluor 488 monoclonal antibody, washed, and subjected to a flow cytometry analyses as outlined in the methods section. Unstained cells for each treatment condition were used to analyze background fluorescence and set gating parameters. The normalized blank-subtracted *FITC*_+_ single cell percentages were used to determine the amount of membrane-TRAIL positive cells for each treatment condition which are represented as bar graphs. Stained cells for each experiment were analyzed in triplicate (*n* = 3) to obtain the SEM, and treatments were compared to the control using Welch’s *t*-test (two sample, two tailed, unequal variance) to evaluate significance with *p* < 0.05. Raw flow-cytometry overlays are shown on the right, with unstained controls for each concentration appearing as black, unfilled traces, while stained samples for each condition are filled and color-coded accordingly. Quercetin treatment of astrocytes did not significantly alter the levels of endogenous membrane-TRAIL on the cell surface. FITC: fluorescein isothiocyanate; SEM: standard error of the mean; TRAIL: tumor necrosis factor-related apoptosis inducing ligand.

The cell surface expression of membrane-TRAIL was not significant in either astrocyte cell line in the absence or presence of quercetin (Figure 5, panels A and B). A small but relatively substantial (14-fold) increase in surface membrane-TRAIL expression was observed in the Astro-2 cell line following quercetin treatment (Figure 5B), but this change did not translate into any noticeable increase in cell death (Figure 1B, lane 2).

**Figure 6:**
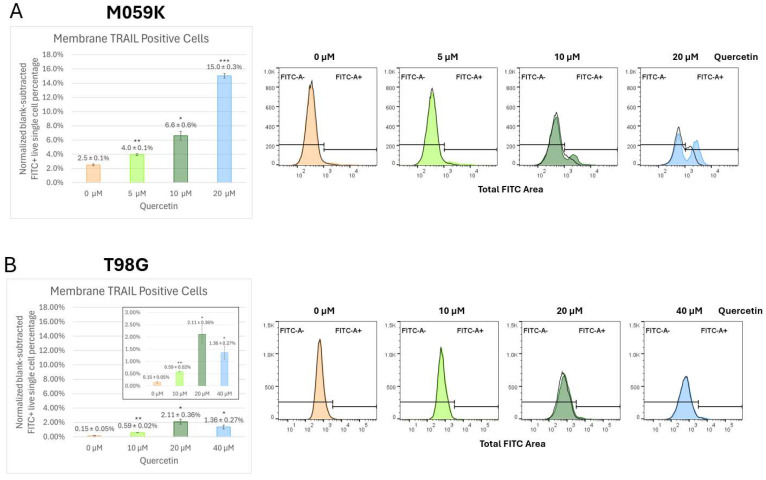

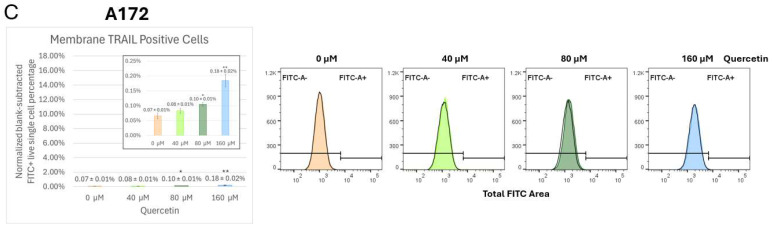
Quercetin increases the expression of membrane-TRAIL on the surface of GBM cells. The GBM cell lines (**A**) M059K, (**B**) T98G, and (**C**) A172 were treated with quercetin alone for 72 h. Cells were collected, washed, and Fc-receptors were blocked with human seroblock. Cells were stained with a mouse anti-human TRAIL Alexa Fluor 488 monoclonal antibody, washed, and subjected to flow cytometry analyses outlined in the methods section. Unstained cells for each treatment condition were used to analyze background fluorescence and set gating parameters. The normalized blank-subtracted *FITC*_+_ single cell percentages were used to determine the amount of membrane-TRAIL positive cells for each treatment condition, which are represented as bar graphs. Stained cells for each experiment were analyzed in triplicate (*n* = 3) to obtain the SEM, and treatments were compared to the control using Welch’s *t*-test (two-sample, two-tailed, unequal variance) to evaluate significance with *p* < 0.05 depicted as *, *p* < 0.01 as **, and *p* < 0.001 as ***. Inserts were utilized for the T98G and A172 graphs to better display the smaller scale differences. Raw flow-cytometry overlays are shown on the right, with unstained controls for each concentration appearing as black, unfilled traces, while stained samples for each condition are filled and color-coded accordingly. Quercetin treatment altered the levels of endogenous membrane-TRAIL expression on the cell surface in all the GBM cell lines to varying degrees. FITC: fluorescein isothiocyanate; GBM: glioblastoma isocitrate dehydrogenase (IDH)-wild type; SEM: standard error of the mean; TRAIL: tumor necrosis factor-related apoptosis-inducing ligand.

In the M059K, surface membrane-TRAIL increased as a function of quercetin concentration and incubation time. After 24 h, cells treated with 10 and 20 µM quercetin showed an increase in membrane TRAIL, from 1.4% to 3.3% and 3.8%, respectively (Figure S2, 24 h). After 48 h, cells treated with 10 and 20 µM quercetin showed significant increases in expression of membrane-TRAIL on the cell surface as compared to the control, with corresponding rises from 0.9% to 4.8% and 10.5% (Figure S2, 48 h). After 72 h, the increase in surface membrane-TRAIL was statistically significant in all quercetin-treated cells compared to the control, with the 20 µM quercetin treatment displaying the most substantial change, with a 6-fold increase, from 2.5% to 15% (Figure 6, panel A). This time- and concentration-dependent release of membrane-TRAIL to the surface in response to quercetin may, at least in part, explain the reason that 72-h incubation with quercetin in the M059K cells induced significantly more apoptosis compared to treatment for only 24 or 48 h (Figure 2A, lanes 2–4; Figure S1, panels A and B).

The T98G cells treated with 10, 20, and 40 µM quercetin for 72 h showed approximately 4-fold, 14-fold, and 9-fold increases, respectively, in cell surface membrane-TRAIL expression compared to the control (Figure 6, panel B). The greatest increase was observed in the 20 µM quercetin treatment, which rose from the basal level of 0.15% to 2.11%. Interestingly, a decline in surface membrane-TRAIL expression to 1.36% was seen when the concentration was increased to 40 µM quercetin, although expression was still markedly higher than in the control. The A172 cells showed the lowest basal level of membrane-TRAIL expression and the least amount of change in response to the 72-h quercetin treatment when compared to the M059K and T98G cell lines. Even at the highest concentration of 160 µM quercetin, only about a 2.5-fold increase was observed compared to the control (Figure 6, panel C). These original and surprising results clearly demonstrate that quercetin releases membrane-TRAIL to the surface of all the GBM cell lines tested. This provides an explanation for how quercetin alone activates the extrinsic pathway of apoptosis in these GBM cell lines in the absence of an exogenously added death ligand (Figure 2) and reinforces our earlier notion that quercetin alone was inducing apoptosis in a similar manner to that of rhTRAIL alone.

### 3.6. Quercetin Increases Trafficking of Endogenous Membrane-TRAIL to the Cell Surface of Cancer Cells but Not Normal Astrocytes

**Figure 7:**
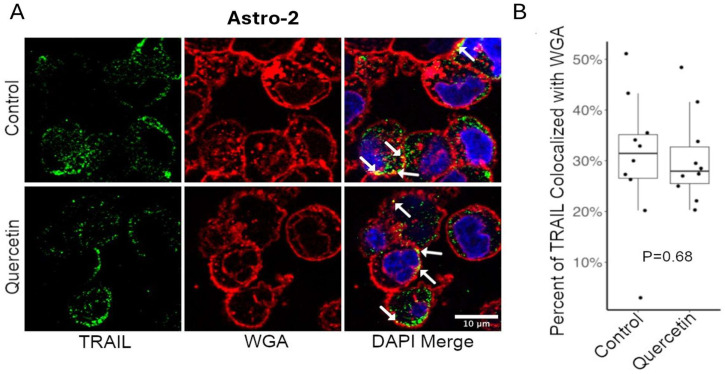
In astrocytes, quercetin has no effect on endogenous surface TRAIL localization. Astro-2 cells were plated on glass chamber slides coated with 20 μg/mL laminin in DMEM with 10% FBS for 2 days. Cells were then treated with 50 µM quercetin or DMSO for 24 h. Subsequently cells were washed, fixed, reacted with WGA Alexa Fluor 627 (WGA is a cell surface marker), washed, and then reacted with goat anti-human TRAIL IgG overnight at 4 °C, followed by reaction with donkey anti-goat IgG Alexa Fluor 488 (1 h, 22 °C), DAPI nuclear stain, and cover-slipped. Cells were imaged on a confocal microscope, and the images analyzed using ImageJ software. (**A**) TRAIL is stained green, WGA is stained red, and colocalization appears as yellow. Representative images are shown. Scale bar denotes 10 µm. Arrows point to colocalization of endogenous TRAIL with WGA, consistent with cell surface TRAIL localization. (**B**) Data was graphed as box and whisker plots. Statistical analysis: Wilcoxon rank sum-test (*p* = 0.68). This data shows that quercetin treatment of astrocytes does not alter the levels of endogenous TRAIL colocalized with surface WGA. DAPI: 4′,6-diamidino-2-phenylindole; DMEM: Dulbecco’s Modified Eagle Medium; DMSO: dimethyl sulfoxide; FBS: fetal bovine serum; TRAIL: tumor necrosis factor-related apoptosis inducing ligand; WGA: wheat germ agglutinin.

The effect of quercetin on membrane-TRAIL localization in normal astrocytes was investigated through confocal microscopy analysis. Astro-2 cells were double-labeled for membrane-TRAIL (Figure 7A, TRAIL panel, green) and the fluorescent-labeled lectin, wheat germ agglutinin (WGA) (Figure 7A, WGA panel, red), following a 24-h treatment with a DMSO vehicle control or 50 µM quercetin. WGA specifically binds cell surface glycoproteins and can be used to mark the cell surface [25,31]. Membrane-TRAIL staining (green) was predominantly detected near the cell surface in a sub-membranous location or at the cell surface embedded within the WGA staining (red), indicating colocalization of membrane-TRAIL and WGA (Figure 7A, DAPI merge panel, yellow, white arrows), along the surfaces of several cells per field. Membrane-TRAIL staining was also detected in vesicles within the cytoplasm of cells, consistent with the trafficking of membrane-TRAIL from the Golgi apparatus to the cell surface. Quantitation of the colocalization of membrane-TRAIL with WGA using ImageJ indicated there was no change in the percent colocalization of membrane-TRAIL with WGA after quercetin treatment (Figure 7B). This suggests that treatment with quercetin for 24 h does not significantly alter the cell surface endogenous membrane-TRAIL expression in normal astrocytes.

**Figure 8:**
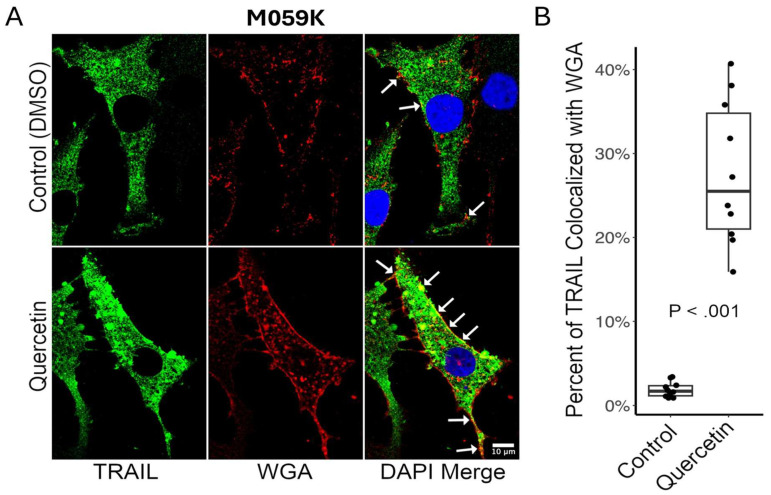
Quercetin increases colocalization of endogenous TRAIL with cell surface WGA in M059K GBM cells. The M059K cells were plated on glass chamber slides coated with 20 μg/mL laminin in DMEM-F12 with 10% FBS for 2 days. Cells were then treated with 20 µM quercetin or DMSO for 24 h. Subsequently cells were washed, fixed, reacted with goat anti-human TRAIL IgG and WGA Alexa Fluor 627 overnight at 4 °C, followed by reaction with anti-goat IgG Alexa Fluor 488 (1 h, 22 °C), DAPI nuclear stain, and cover-slipped. Cells were imaged on a confocal microscope, and the images analyzed using ImageJ software. (**A**) TRAIL is stained green, WGA is stained red, and colocalization appears as yellow. Representative images are shown. Scale bar denotes 10 µm. Arrows point to colocalization of endogenous TRAIL with WGA. (**B**) The data was graphed as box and whisker plots. Statistical analysis: Wilcoxon rank sum-test (*p* < 0.001). This data shows that quercetin treatment of the M059K cells results in a significant increase in the colocalization of endogenous TRAIL with surface WGA. DAPI: 4′,6-diamidino-2-phenylindole; DMEM: Dulbecco’s Modified Eagle Medium; DMSO: dimethyl sulfoxide; FBS: fetal bovine serum; GBM: glioblastoma isocitrate dehydrogenase (IDH)-wild type; TRAIL: tumor necrosis factor-related apoptosis inducing ligand; WGA: wheat germ agglutinin.

M059K cells were double labeled for membrane-TRAIL (Figure 8A, TRAIL panel, green) and lectin WGA (Figure 8A, WGA panel, red) to determine whether the increase in cell surface membrane-TRAIL protein observed after treatment with 20 µM quercetin for 24 h in the flow cytometry analysis (Figure S2A) was due to trafficking of the protein to the cell surface. M059K cells were treated with a DMSO vehicle control or 20 µM quercetin for 24 h, followed by confocal microscopy analysis. Very little colocalization of membrane-TRAIL with WGA on the cell surface was observed in the control (Figure 8A, DAPI merge panel, top section, yellow, white arrows) but quercetin treatment resulted in a large increase in the colocalization of membrane-TRAIL with cell surface WGA (Figure 8A, DAPI merge panel, bottom section, yellow, white arrows) with an approximate 8.5-fold increase, from 3% to 26%, in colocalization (Figure 8B). These findings are consistent with the Western blot and membrane-TRAIL flow cytometry analysis, strongly suggesting that quercetin induces increased trafficking of endogenous membrane-TRAIL to the cell surface in GBM cell lines.

### 3.7. Quercetin Effects Membrane DR4 and DR5 Expression

Since functional DRs on the cell surface are required for membrane-TRAIL to bind and induce apoptosis, membrane DR4 and DR5 expression in M059K, T98G, A172, and normal astrocytes were measured following a 72-h treatment with either a DMSO control or varying amounts of quercetin. The normalized blank-subtracted PE+ live single cell percentages were calculated to determine the portion of DR4 or DR5 positive cells for each treatment condition. Histograms were produced to display the mean percentage of membrane DR4 or DR5 positive cells ± SEM for the different cell lines under each condition. Raw histogram data obtained from each analysis, with an overlay of unstained samples (black unfilled traces) and stained samples (filled and color-coded accordingly) were provided.

In the M059K cells, quercetin increased DR4 surface expression from the basal level of 0.58% to 1.35%, 3.20%, and 1.49% in the 5 µM, 10 µM, and 20 µM treatments, respectively (Figure S3, panel A, top section). At 10 µM quercetin, an average 5.5-fold increase in surface DR4 was seen, while the 20 µM concentration dropped to only a 2.5-fold increase with a relatively large standard deviation, indicating high variability among cellular DR4 expression at this concentration. Quercetin steadily decreased membrane expression of DR5 in the M059K from 95.27% in the DMSO control to 90.89% in the 5 µM, 82.21% in the 10 µM, and 60.59% in the 20 µM (Figure S3, panel A, bottom section). In the T98G cells, quercetin gradually increased DR4 surface expression from the DMSO control level of 0.21% to 0.60%, 0.94%, and 1.32% in the 10 µM, 20 µM, and 40 µM treatments, respectively (Figure S3, panel B, top section). Levels of membrane DR5 in the T98G progressively decreased from 98.37% in the DMSO control to 98.22% in the 10 µM, 89.69% in the 20 µM, and 82.49% in the 40 µM (Figure S3, panel B, bottom section). In the A172 cells, treatment with 40 and 80 µM quercetin increased the amount of membrane DR4 from the base level of 0.04% to 0.16% and 0.22%, respectively. Membrane DR4 expression in the 160 µM quercetin treatment rose to 1.01% showing the most significant change in DR4 expression among all the tested cell lines, exhibiting a 25-fold increase compared to the control (Figure S3, panel C, top section). Surface DR5 expression in the A172 DMSO control was 99.80%, making it the highest of all the GBM lines tested. Quercetin treatment gradually decreased DR5 membrane expression to 99.65% in the 40 µM, 99.22% in the 80 µM, and 95.06% in the 160 µM (Figure S3, panel C, bottom section). This data clearly shows that membrane DR5 is present in abundance on the surface of all the tested GBM cell lines. More importantly, the significant decrease in DR5 seen with increasing quercetin concentrations may be a result of ligand-mediated DR5 endocytosis [32], corroborating our previous results (Figure 2 and Figure 3) that demonstrate the ability of quercetin alone to induce apoptosis in GBM cell lines in a TRAIL-like manner.

In the Astro-1 cells, the surface expression of membrane DR4 and DR5 were both unexpectedly high. The amount of DR4 on the surface in the DMSO control was greater than any of the GBM cell lines, with a basal level of 10.21% which was increased to 19.29% (1.89-fold) when treated with 50 µM quercetin (Figure S4, panel A, top section). Membrane DR5 started at 93.65% in the DMSO control and was significantly decreased following quercetin treatment to 72.35% (1.29-fold) (Figure S4, panel A, bottom section), in a pattern similar to the GBM cells. In contrast, the starting expression of DR4 in the Astro-2 cells was only 0.15%, which rose to 0.59% following treatment with 50 µM quercetin, a change that was not statistically significant (Figure S4, panel B, top section). Astro-2 also displayed a remarkably low DR5 basal expression of 0.05%, which did increase significantly to 0.72% after treatment with 50 µM quercetin (Figure S4, panel B, bottom section), but this did not translate into any observed increase in cell death (Figure 1B, lane 2). These results, at least partially, clarify the discrepancy between the two astrocyte cell lines since Astro-1 possesses significantly more DR4 and DR5 on its surface compared to Astro-2. The Astro-1 cells are sensitive to rhTRAIL, while Astro-2 cells are relatively resistant.

## 4. Discussion

Our data conclusively show that quercetin alone induces apoptosis in a TRAIL-like manner in all GBM cell lines tested. The apoptotic induction in response to quercetin treatment varied greatly depending on the cell line, and this was further verified through flow cytometry analysis. In contrast, quercetin did not induce apoptosis in non-tumorigenic human astrocyte cell lines. Surprisingly, rhTRAIL treatment of normal astrocytes led to a small amount of apoptosis in one of the control cell lines (Astro-1), while no apoptosis was observed in the other control cell line (Astro-2), indicating an inherent difference between the two control cell lines (Figure 1, panels A and B). Historically, astrocytes were viewed as a homogenous population of cells, but in recent years, their heterogeneity has been established. The molecular profiles of astrocytes vary both between people and within the same individual for a variety of reasons, including age, health, brain location, and regional chemical signals [33]. Generally, human astrocytes are resistant to TRAIL-induced apoptosis, but certain conditions can restore their sensitivity [34]. Interestingly, our data demonstrated that cotreatment with quercetin mitigated the apoptosis observed with the TRAIL-sensitive Astro-1 cells in response to rhTRAIL treatment (Figure 1A, lanes 6–9), suggesting that quercetin may exert a protective effect on astrocytes that have lost their TRAIL resistance.

**Figure 9:**
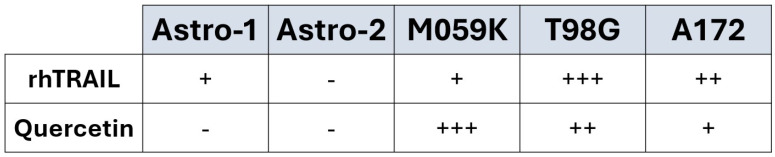
Summary of astrocyte and GBM relative sensitivity to apoptosis following treatment. A minus sign (–) denotes that no detectable apoptosis was seen in response to treatment, while a plus sign (+) specifies that apoptosis was observed. The number of plus signs increases in accordance with the detected amount of apoptosis, with consideration of the effective concentrations required for each treatment and cell line. Three plus signs (+++) for a particular treatment in a cell line indicate that comparatively lower concentrations were required to achieve a substantial amount of apoptosis. GBM: glioblastoma isocitrate dehydrogenase (IDH)-wild type.

GBM cell lines are notoriously resistant to TRAIL [1], but our laboratory has previously demonstrated the ability of quercetin to sensitize other TRAIL-resistant tumor cell types, including breast cancer and malignant melanoma, to rhTRAIL-induced apoptosis [11,14]. In accordance, the main goal of this study was to explore quercetin as a potential treatment for GBM. Surprisingly, in all the GBM cell lines, quercetin by itself was able to activate both the extrinsic and intrinsic pathways of apoptosis in the absence of exogenously added rhTRAIL, but the effective concentrations were significantly different depending on the GBM cell line. Based on effective concentrations for substantial apoptotic induction, M059K cells were the most sensitive to quercetin treatment, followed by T98G, and lastly A172 (Figure 9). By itself, rhTRAIL also induced apoptosis through both pathways in all the GBM cell lines tested, but again, the effective treatment concentrations were cell line dependent. Consistent with previously published data [35], we found that M059K were comparatively rhTRAIL-resistant while T98G and A172 were both more rhTRAIL-sensitive, with T98G showing the greatest sensitivity to rhTRAIL (Figure 9). Most notably, in every GBM cell line, the addition of quercetin significantly enhanced rhTRAIL-induced apoptosis. In line with these observations, flow cytometry analyses demonstrated that quercetin alone and rhTRAIL alone activated apoptosis in the GBM cells in a concentration-dependent manner and that apoptotic induction was substantially strengthened with the cotreatment.

It is important to note that a variety of underlying genetic differences exist between the GBM cancer cell lines studied herein and normal astrocytes that may be partially responsible for the observed variations in quercetin and rhTRAIL sensitivity. The tumor suppressor genes TP53 and PTEN, which encode for p53 and PTEN proteins, respectively, are central regulators of cell growth, division, DNA repair, and programmed cell death, so mutations in these genes are often associated with cancer treatment resistance [36]. The DNA repair gene MGMT encodes the DNA repair protein MGMT that removes alkyl groups from DNA to prevent mutations. Consequently, GBM cells that express high levels of MGMT are resistant to TMZ [37,38]. Human astrocytes have wild-type PTEN [39], TP53 [37], and generally express MGMT protein [37]. M059K carries homozygous loss-of-function TP53 [40], homozygous PTEN frameshift [41], and has been reported to be moderately TMZ-sensitive [38]. T98G has mutations in both TP53 and PTEN [42], and shows high MGMT protein expression, making it the canonical TMZ-resistant GBM model [38,43]. A172 cells do not have any reported TP53 mutations but carry a homozygous PTEN deletion [42], and expression of MGMT protein is extremely low or completely absent due to MGMT promoter hypermethylation, making these cells TMZ-sensitive [38,43]. In cancer cells, the mutated p53 proteins are often protected from degradation and accumulate [44]. In T89G cells, the TP53 mutation has been linked to oncogenic gain-of-function amyloid mutant p53 oligomer formation [45]. PTEN loss or mutation in GBM drives AKT signaling that increases stability of the anti-apoptotic protein, cFLIP_S_, which blunts caspase-8 activation and promotes resistance to TRAIL [46]. PTEN loss increases the AKT-dependent function of the transporter protein, ABCG2, through translocation to the cell membrane, where it can pump compounds outside the cell, diminishing drug sensitivity [47]. At low concentrations, ABCG2 affects intracellular concentrations of quercetin [48], but at higher concentrations, quercetin exhibits an inhibitory effect on ABCG2 [49]. All these genetic mutations detailed above could contribute one way or another to the quercetin/membrane-TRAIL sensitivity of the cancer cells tested. However, while the bulk of literature knowledge on a given subject is of the utmost importance, careful experimental analysis with the appropriate controls must be performed in each case to elucidate the exact role and mechanism of quercetin in promoting membrane-TRAIL trafficking in various subtypes of GBM.

Following the observation that quercetin alone induced apoptosis through the extrinsic pathway in a TRAIL-like manner in the GBM cells in the absence of an exogenously added death ligand, we hypothesized that quercetin was facilitating the trafficking of endogenous TRAIL within the GBM cells. Membrane-TRAIL is present in a variety of human tissues at the transcriptional level [50], and endogenous membrane-TRAIL expression has been demonstrated in various cells within the body [51], including the brain [52,53,54,55,56,57]. The presence of various forms of endogenous membrane-TRAIL in all the GBM cell lines without stimulation or treatment was confirmed through Western blotting. Flow cytometry analysis was subsequently employed to assess the trafficking of membrane-TRAIL to the cell surface of the GBM cells in response to treatment with quercetin for 72 h. Since the M059K cells had the greatest base level expression of endogenous membrane-TRAIL, the most substantial apoptosis in response to quercetin alone, and showed the most significant increase in surface membrane-TRAIL expression, we decided to investigate these cells further at earlier time points (24 and 48 h). We found that the trafficking of TRAIL to the cell membrane increased as a function of time, which was in line with our observation that substantial apoptotic initiation in these cells requires incubation with quercetin for 72 h. In all the GBM cell lines, increasing the concentrations of quercetin resulted in an increase in surface membrane-TRAIL after 72 h. The T98G cells had the second-highest basal expression of endogenous membrane-TRAIL and showed the greatest fold increase in surface membrane-TRAIL of all the GBM cell lines after quercetin treatment (15-fold increase at 20 µM quercetin compared to the control), displaying moderate induction of apoptosis with quercetin alone compared to the M059K. The A172 cells had the lowest endogenous membrane-TRAIL expression, showed the smallest change in surface membrane-TRAIL after quercetin treatment, and were the least sensitive to apoptotic induction in response to quercetin alone. Overall, this data emphasized the importance of basal levels of endogenous membrane-TRAIL in the effectiveness of quercetin alone in GBMs. The GBM cells that contained higher amounts of endogenous membrane-TRAIL exhibited increased surface expression of membrane-TRAIL in response to quercetin and showed the strongest apoptotic response to quercetin treatment alone. At this point, it is worth emphasizing that membrane-TRAIL has been shown to possess markedly greater proapoptotic potency than soluble TRAIL, largely due to its ability to form supramolecular structures [58,59,60].

Since activation of the extrinsic pathway of apoptosis relies on DRs, flow cytometry was used to assess membrane DR expression in GBM cells following quercetin treatment. These analyses revealed that M059K, T98G, and A172 GBM cells possessed a substantial amount of basal DR5 on their surface and a minimal amount of DR4. In general, for all the GBM cell lines, increasing concentrations of quercetin led to minor increases in membrane DR4 expression and significant decreases in membrane DR5 expression. In these GBM cells, the reduced surface expression of DR5 after quercetin treatment may be due to ligand-mediated endocytosis [32] triggered by DR5 binding to the endogenous membrane-TRAIL trafficked to the cell membrane after quercetin treatment. It is important to note that efficient DR5 activation requires membrane-bound TRAIL or cross-linked soluble TRAIL, while DR4 can be activated by non-cross-linked soluble TRAIL [58,59,60].

In the astrocyte control cells, we found that Astro-1 cells expressed higher levels of DR4 and DR5 compared to Astro-2 cells (Figure S4, panels A and B), which explains their greater sensitivity to rhTRAIL. Astro-1 cells exhibited endogenous membrane-TRAIL levels comparable to the M059K cells (Figure 4), expressed the highest DR4 levels among all tested cell lines, which was further increased by quercetin treatment (Figure S4A, upper section), and had elevated basal DR5 expression that decreased following quercetin exposure in a similar fashion to the GBM cells (Figure S4B, lower section). However, quercetin did not promote trafficking of endogenous TRAIL to the cell surface membrane in these cells (Figure 6A), and they did not undergo apoptosis in response to quercetin treatment (Figure 1A, lanes 4 and 5). Given their sensitivity to rhTRAIL (Figure 1A, lanes 2 and 3), these findings suggest that if quercetin had induced the trafficking of endogenous TRAIL to the surface, apoptosis would have occurred. This highlights an underlying difference between the non-transformed astrocytes and the GBM cells that is exploited by quercetin to selectively induce apoptosis in cancer cells.

In normal human T blast cells and in the human T cell leukemia cell line, Jurkat, bioactive endogenous membrane-TRAIL is stored in the cytoplasm in post-Golgi, pre-lysosomal multivesicular bodies, which are mobilized to the plasma membrane and released upon stimulation with the T cell activator phytohemagglutinin [61,62]. The presence, cellular location, and release of endogenous membrane-TRAIL in other cell lines have not been extensively investigated, but several cell lines have been shown to possess endogenous membrane-TRAIL at varying levels [29,51,63,64]. It has been well documented that quercetin can not only pass the cell membrane and be taken up by cells, but it can also intercalate into the cell membrane and cause structural changes to the lipid bilayer [65,66,67,68,69,70,71,72,73,74,75,76]. The extent to which quercetin enters or embeds into the membrane depends on a variety of factors, including the concentration of quercetin [65,66,73], the pH of the environment [66,67,69], and the membrane lipid composition [72,73,74]. These variables may, at least in part, account for the differential responses to quercetin observed among the GBM and normal astrocyte cell lines in our study. Both our flow cytometry and confocal analyses revealed that quercetin induced trafficking of endogenous membrane-TRAIL to the cell surface in the GBM cells, but not in the astrocytes. This puzzling contrast can be easily explained by the different environments present on the surface of normal cells as compared to GBM cells. As stated above, quercetin’s ability to penetrate the membrane is highly pH dependent. In the acidic microenvironment typical of GBM cells [77], the quercetin molecule becomes protonated, rendering it overall neutral and allowing it to integrate into the hydrophobic core of the lipid bilayer. In contrast, astrocytes maintain alkaline conditions on their surface by releasing bicarbonate to buffer excess acidity [78]. Under these conditions, quercetin remains deprotonated and negatively charged, favoring interactions with polar head groups rather than membrane insertion [79]. This pH-dependent behavior may help explain why endogenous membrane-bound TRAIL is not mobilized to the surface in Astro-1 cells following quercetin treatment.

**Figure 10:**
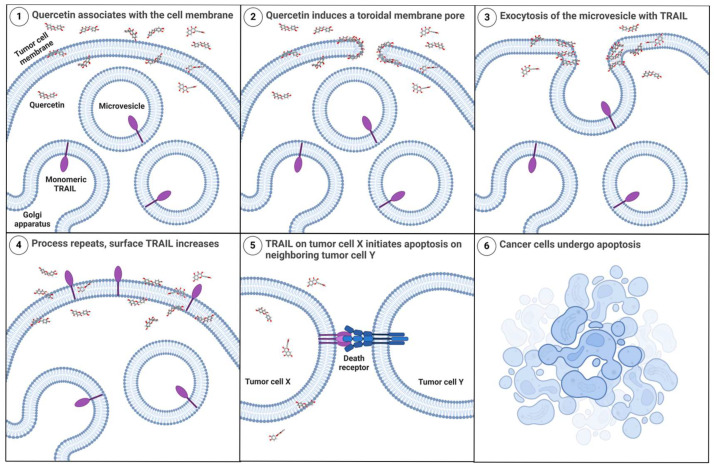
Hypothetical mechanism for the release of endogenous membrane-TRAIL in cancer cells in response to quercetin treatment. (**1**) Following treatment, the quercetin begins to accumulate both inside and around the tumor cell membrane. Endogenous monomeric membrane-TRAIL is released in microvesicles from the Golgi apparatus into the cytoplasm. (**2**) Once an adequate amount of quercetin has gathered and embedded into the lipid bilayer, a toroidal pore is formed by quercetin, where the membrane bends into itself. This pore is lined with both quercetin and the polar heads of the phospholipid bilayer. (**3**) A driving force causes the internal contents of the cytoplasm, including the microvesicles containing membrane-TRAIL, to be pulled towards the pore created by quercetin. The phospholipid bilayer of the microvesicle merges with the cell membrane, closing the pore and releasing membrane-TRAIL to the cell surface via exocytosis. (**4**) In a process dependent on both time and concentration, quercetin continues to accumulate and create multiple pores across the cell membrane, leading to the release of more endogenous monomeric membrane-TRAIL from the cytoplasm to the cell surface. (**5**) When “tumor cell X” containing surface membrane-TRAIL encounters a neighboring “tumor cell Y” with a functional membrane DR, the trimerized TRAIL binds to the trimerized DR, initiating apoptosis in the neighboring cell. (**6**) This ultimately results in the cancer cells undergoing self-assisted apoptotic cell death. Figure created in https://BioRender.com, accessed on 30 September 2025. DR: death receptor; TRAIL: tumor necrosis factor-related apoptosis-inducing ligand.

A recent electrophysiological study with planar lipid membranes indicated that quercetin was able to penetrate the hydrophobic core of the membrane and form a channel-like event [76]. Based on their signal pattern observations, the authors speculated that quercetin could be forming a transmembrane toroidal pore [76,80]. In this proposed toroidal configuration, quercetin would cause the bilayer to bend into itself, creating a pore that is lined by both quercetin and the polar hydrophilic phospholipid head groups [76,80]. The puncture of the lipid bilayer caused by sufficient accumulation of quercetin may be the mechanism by which membrane-TRAIL is trafficked to the cell surface, where it then can bind to DRs present on neighboring cells and induce apoptosis (Figure 10, created using BioRender, RRID:SCR_018361). It seems that this process depends on both the abundance of quercetin and incubation time, as different cells have variable sensitivity to quercetin and expression levels of endogenous membrane-TRAIL.

The increase in effectiveness of quercetin treatment alone after 48 or more hours has previously been demonstrated by other laboratories in various cancer cell types, including ovarian [81], lung [82], bladder [83], and breast cancers [84,85]. However, no clear explanation has yet been provided for the necessity of the prolonged time of incubation with quercetin. We hypothesize that 72 h is the time necessary for endogenous membrane-TRAIL to be trafficked to the cell surface membrane and accumulate in significant enough concentrations capable of inducing apoptosis in neighboring cells possessing DRs. Additionally, the intrinsic pathway of apoptosis only became activated after incubation with quercetin for 72 h, further emphasizing the significance of this time point as an important threshold to overcome.

Our data unexpectedly demonstrates that quercetin alone can induce apoptosis in GBM cell lines in a TRAIL-like manner through the trafficking of endogenous TRAIL to the cell membrane. While exogenous rhTRAIL cannot readily cross the BBB [86], the ability of quercetin to cross the BBB and enter the brain has been consistently demonstrated, *in vitro* [28,87,88]. Additionally, *in vivo* studies in mice, rats, and pigs showed that picomolar to nanomolar concentrations of quercetin could be detected in their brains following oral quercetin administration [28,89,90]. Moreover, in rats, the coadministration of quercetin with α-tocopherol (vitamin E) enhanced the ability of quercetin to cross the BBB [91]. In humans, plasma concentrations of quercetin from diet alone typically fall within the nanomolar range and may vary considerably based on the individual [92,93], but several studies have shown that supplementation with quercetin can significantly increase its plasma concentration [94,95,96]. Importantly, multiple studies have shown that quercetin selectively induces apoptosis in cancer cells while sparing normal cells [18,19]. Thus, the ability of quercetin alone to induce cell death in GBM cells through the intrinsic and extrinsic pathways of apoptosis, because of the trafficking of endogenous membrane-TRAIL to the cell surface of cancer cells, coupled with its overall safety, low cost, and availability, makes quercetin an extremely useful agent for the treatment of patients with GBM.

Our *in vitro* analysis is original and compelling; however, additional preclinical experiments with more cell lines, including patient-derived GBM cells and other non-transformed brain cells, as well as *in vivo* analyses (with mice), should be explored to shed more light on this potential therapeutic effect of quercetin. Key next steps include defining the intracellular role of endogenous membrane-TRAIL in cancer cells and astrocytes, pinpointing its specific transcriptional regulators (if any), and investigating expression of anti-apoptotic proteins in the presence and absence of quercetin. However, determining the differences in cell membrane composition of various cancer cells and normal astrocytes is of the utmost importance. Quercetin has been reported to rapidly inhibit lipid/cholesterol *de novo* synthesis (by downregulating ACC1, HMGCR, and SREBP2), plausibly reducing raft cholesterol over hours, thus altering raft structure by decreasing membrane cholesterol, therefore, modifying bilayer stiffness/elasticity [97,98]. These findings support the hypothesis that cholesterol-reliant cells (like GBM cells) may be functionally modulated by quercetin.

## 5. Conclusions

Our study demonstrated that while the combination of quercetin and rhTRAIL was more effective than either treatment alone, quercetin by itself unexpectedly induced apoptosis in all GBM cell lines through both the intrinsic and extrinsic pathways. We found that GBM cells expressed endogenous membrane-TRAIL, and that quercetin promoted its trafficking to the cell surface in a time- and concentration-dependent manner, resulting in self-assisted apoptosis of the GBM tumor through interaction with DRs on neighboring tumor cells. Importantly, in the normal astrocyte control cells, quercetin did not trigger the release of endogenous membrane-TRAIL, did not induce apoptosis, and even protected astrocytes that had lost their resistance to rhTRAIL-induced cell death. Overall, the ability of quercetin to cross the BBB, combined with the novel finding that it can selectively trigger the trafficking of endogenous membrane-TRAIL to the cell surface in GBM cells, while sparing healthy cells, highlights its potential as a valuable therapeutic strategy for treating patients with GBM.

## Data Availability

The original contributions presented in this study are included in the article/Supplementary Material. Further inquiries can be directed to the corresponding author.

## Supplementary Materials

The following supporting information can be downloaded at: https://www.mdpi.com/article/10.3390/cancers17193197/s1, Figure S1: Analysis of apoptosis in M059K after treatment with quercetin for 24 and 48 h; Figure S2: Quercetin-induced increase in membrane-TRAIL expression in M059K is dependent on concentration and time; Figure S3: Effect of quercetin on membrane DR expression in GBMs. Flow cytometry analysis of membrane DR expression in (A) M059K (B) T98G and (C) A172 GBM cells was carried out after 72 h of quercetin treatment; Figure S4: Effect of quercetin on membrane DR expression in astrocytes. Flow cytometry analysis of membrane DR expression in normal astrocyte cell lines (A) Astro-1 and (B) Astro-2 was carried out after 72 h of quercetin treatment.

## Abbreviations

ABCG2	ATP-binding cassette subfamily G member 2
ACC1	acetyl-CoA Carboxylase 1
APAF1	apoptotic protease-activating factor 1
BBB	blood–brain barrier
BCA	bicinchoninic acid
BID	BH3-interacting domain death agonist
cFLIP_S_	cellular FLICE-inhibitory protein short isoform
DAPI	4′,6-diamidino-2-phenylindole
DMEM	Dulbecco’s Modified Eagle Medium
DMSO	dimethyl sulfoxide
DR	death receptor
FADD	Fas-associated death domain
FITC	fluorescein isothiocyanate
FPLC	fast protein liquid chromatography
GBM	glioblastoma isocitrate dehydrogenase (IDH)-wild type
HMGCR	HMG-CoA reductase
HPLC	high-performance liquid chromatography
HRP	horseradish peroxidase
kDa	kilodalton
MGMT	O6-methylguanine-DNA methyltransferase
MW	molecular weight
p53	tumor protein 53
PAGE	polyacrylamide gel electrophoresis
PARP	poly (ADP-ribose) polymerase
PBS	phosphate-buffered saline
PE	phycoerythrin
PI	propidium iodide
PTEN	phosphatase and tensin homolog gene
rhTRAIL	recombinant human TRAIL
RIPA	radioimmunoprecipitation assay
SDS	sodium dodecyl sulfate
SEM	standard error of the mean
SREBP2	sterol regulatory element-binding protein 2
TMZ	temozolomide
TP53	tumor protein 53 gene
TRAIL	tumor necrosis factor-related apoptosis-inducing ligand
WGA	wheat germ agglutinin
