# Supplementary material for: Quercetin Increases Expression of Membrane-TRAIL in Glioblastoma Cells Resulting in Apoptosis
Source: Cancers (Basel). 2025 Sep 30;17(19):3197. doi: 10.3390/cancers17193197 (PMC12523679; doi:10.3390/cancers17193197)
Supplement: Supplementary file 1 [file cancers-17-03197-s001.zip › cancers-3854090-SuppFigures.pptx]

## Slide 1
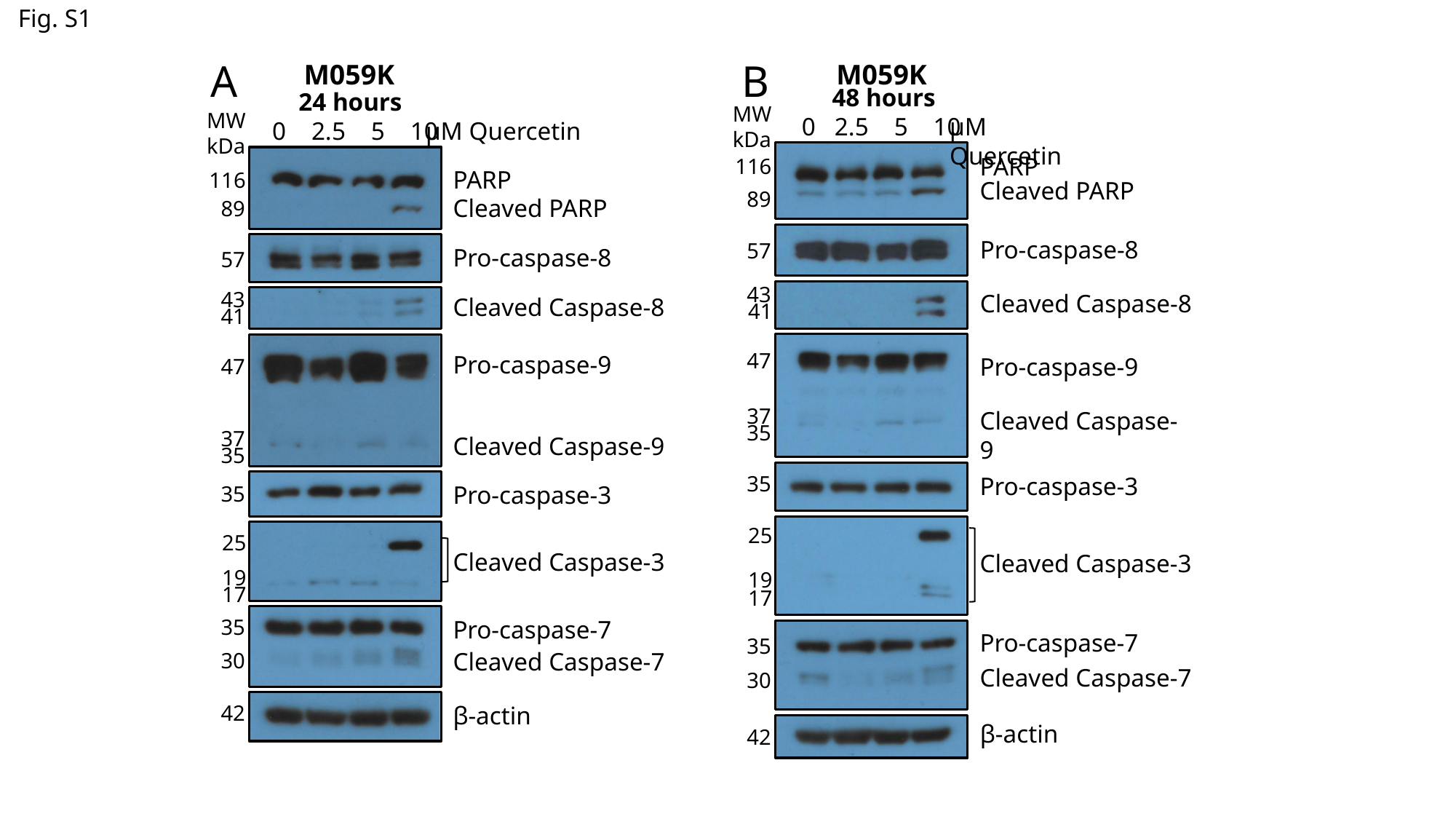

# Fig. S1
M059K
M059K
A
B
48 hours
24 hours
MW
kDa
MW
kDa
0 2.5 5 10
µM Quercetin
0 2.5 5 10
µM Quercetin
PARP
116
PARP
116
Cleaved PARP
89
Cleaved PARP
89
Pro-caspase-8
57
Pro-caspase-8
57
43
43
Cleaved Caspase-8
Cleaved Caspase-8
41
41
47
Pro-caspase-9
Pro-caspase-9
47
37
Cleaved Caspase-9
35
37
Cleaved Caspase-9
35
35
Pro-caspase-3
Pro-caspase-3
35
25
25
Cleaved Caspase-3
Cleaved Caspase-3
19
19
17
17
35
Pro-caspase-7
Pro-caspase-7
35
Cleaved Caspase-7
30
Cleaved Caspase-7
30
42
β-actin
β-actin
42

## Slide 2
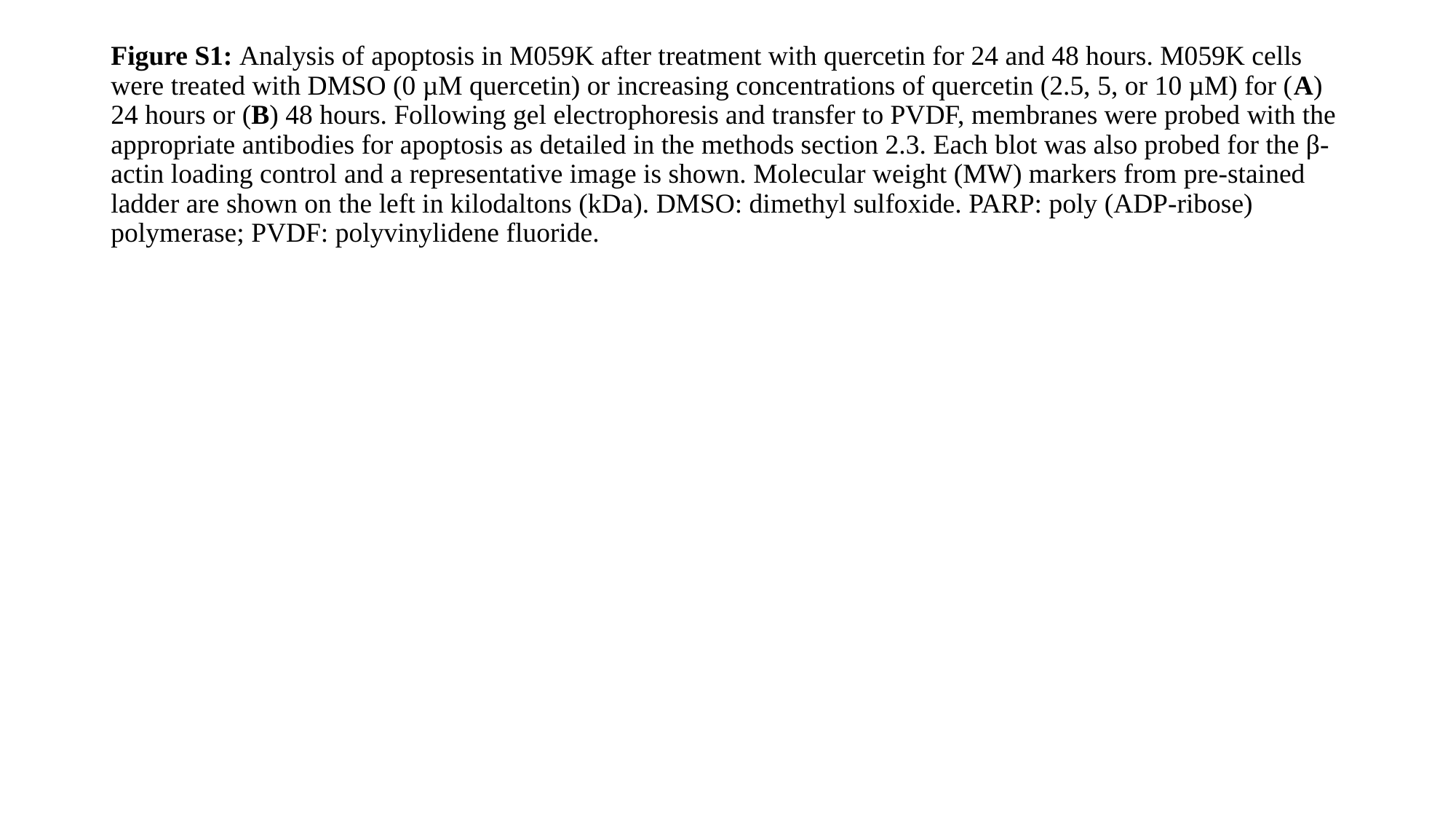

Figure S1: Analysis of apoptosis in M059K after treatment with quercetin for 24 and 48 hours. M059K cells were treated with DMSO (0 µM quercetin) or increasing concentrations of quercetin (2.5, 5, or 10 µM) for (A) 24 hours or (B) 48 hours. Following gel electrophoresis and transfer to PVDF, membranes were probed with the appropriate antibodies for apoptosis as detailed in the methods section 2.3. Each blot was also probed for the β-actin loading control and a representative image is shown. Molecular weight (MW) markers from pre-stained ladder are shown on the left in kilodaltons (kDa). DMSO: dimethyl sulfoxide. PARP: poly (ADP-ribose) polymerase; PVDF: polyvinylidene fluoride.

## Slide 3
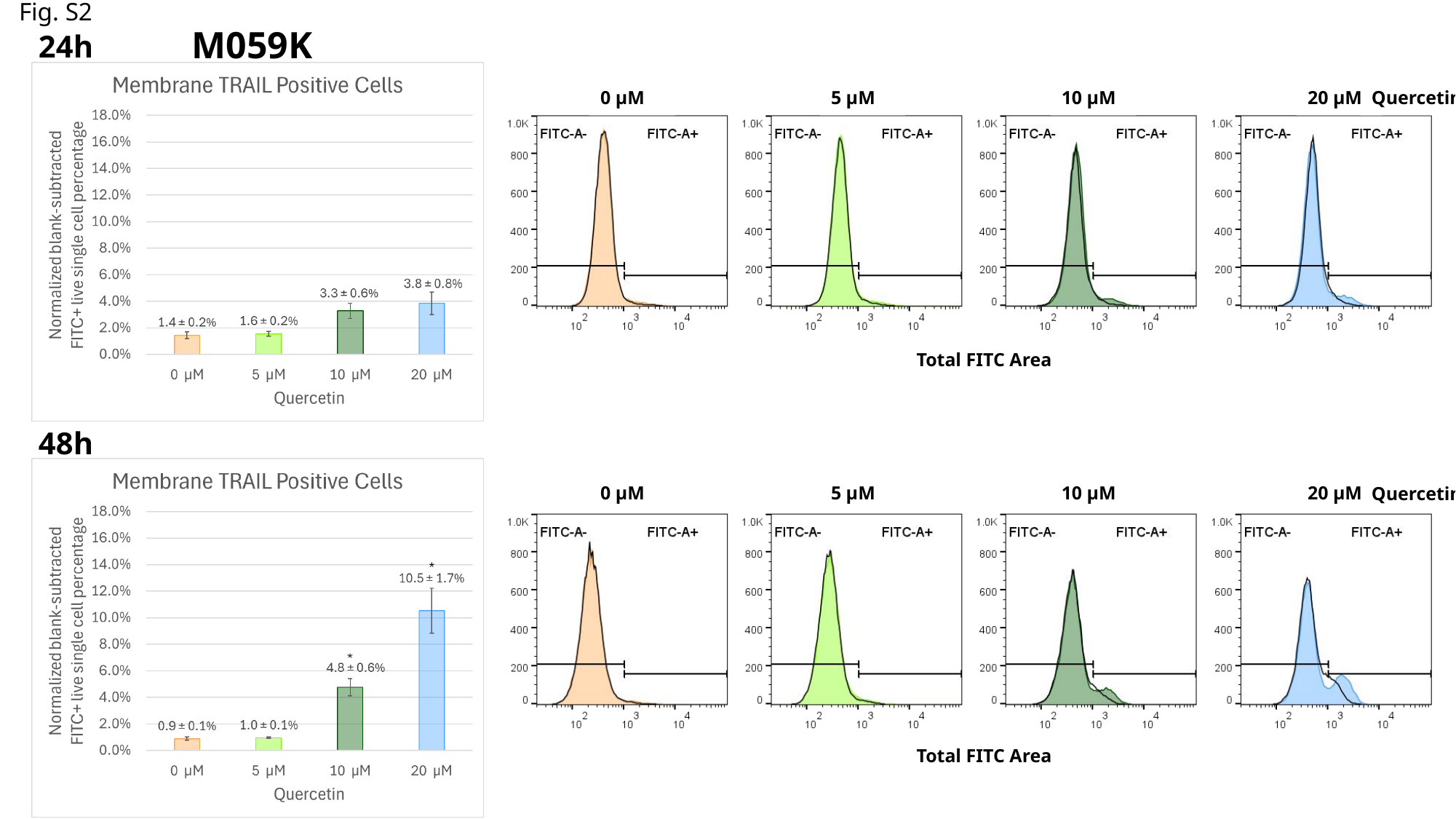

# Fig. S2
M059K
24h
0 µM
5 µM
10 µM
20 µM
Quercetin
Total FITC Area
48h
0 µM
5 µM
10 µM
20 µM
Quercetin
Total FITC Area

## Slide 4
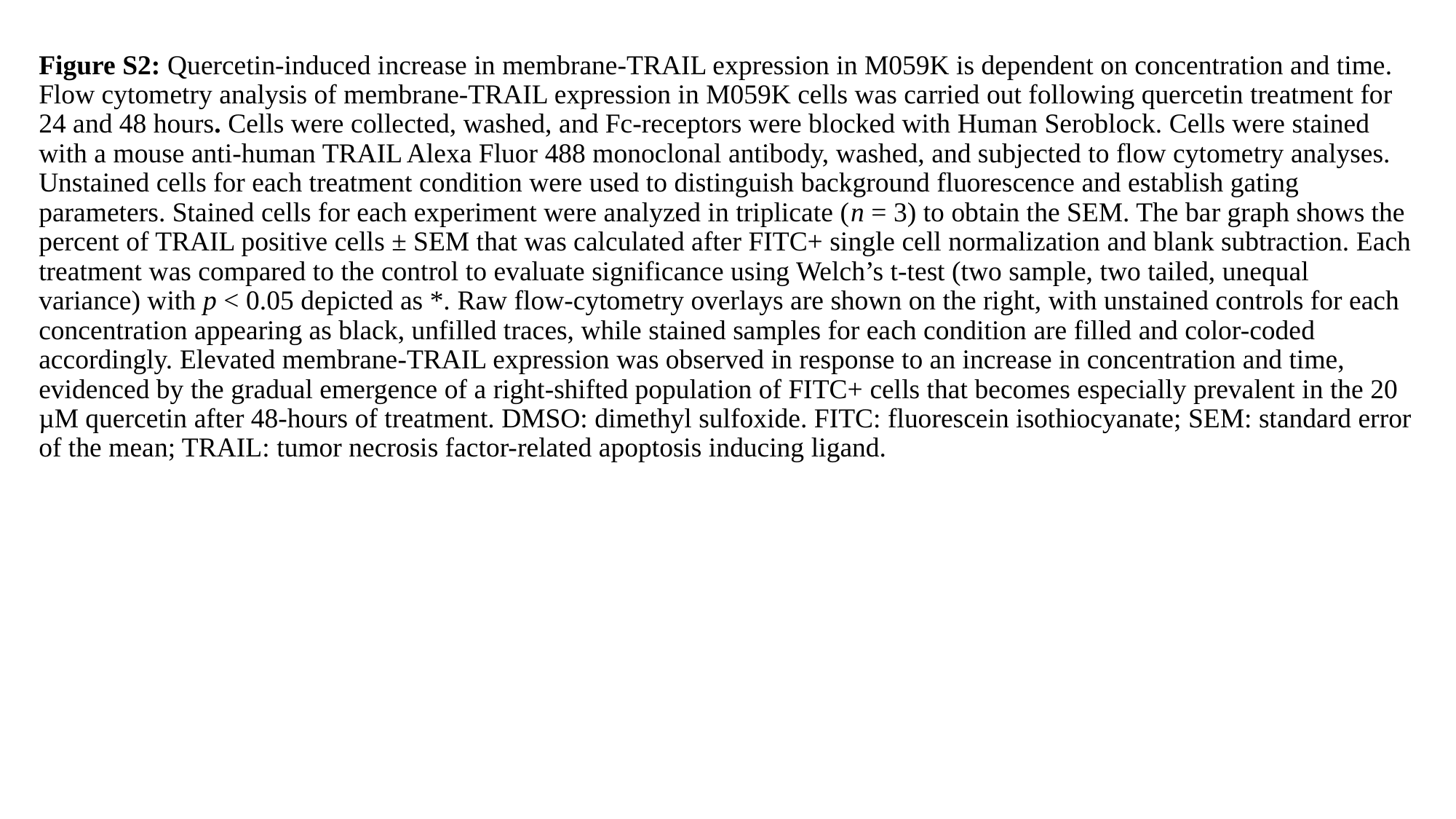

Figure S2: Quercetin-induced increase in membrane-TRAIL expression in M059K is dependent on concentration and time. Flow cytometry analysis of membrane-TRAIL expression in M059K cells was carried out following quercetin treatment for 24 and 48 hours. Cells were collected, washed, and Fc-receptors were blocked with Human Seroblock. Cells were stained with a mouse anti-human TRAIL Alexa Fluor 488 monoclonal antibody, washed, and subjected to flow cytometry analyses. Unstained cells for each treatment condition were used to distinguish background fluorescence and establish gating parameters. Stained cells for each experiment were analyzed in triplicate (n = 3) to obtain the SEM. The bar graph shows the percent of TRAIL positive cells ± SEM that was calculated after FITC+ single cell normalization and blank subtraction. Each treatment was compared to the control to evaluate significance using Welch’s t-test (two sample, two tailed, unequal variance) with p < 0.05 depicted as *. Raw flow-cytometry overlays are shown on the right, with unstained controls for each concentration appearing as black, unfilled traces, while stained samples for each condition are filled and color-coded accordingly. Elevated membrane-TRAIL expression was observed in response to an increase in concentration and time, evidenced by the gradual emergence of a right-shifted population of FITC+ cells that becomes especially prevalent in the 20 µM quercetin after 48-hours of treatment. DMSO: dimethyl sulfoxide. FITC: fluorescein isothiocyanate; SEM: standard error of the mean; TRAIL: tumor necrosis factor-related apoptosis inducing ligand.

## Slide 5
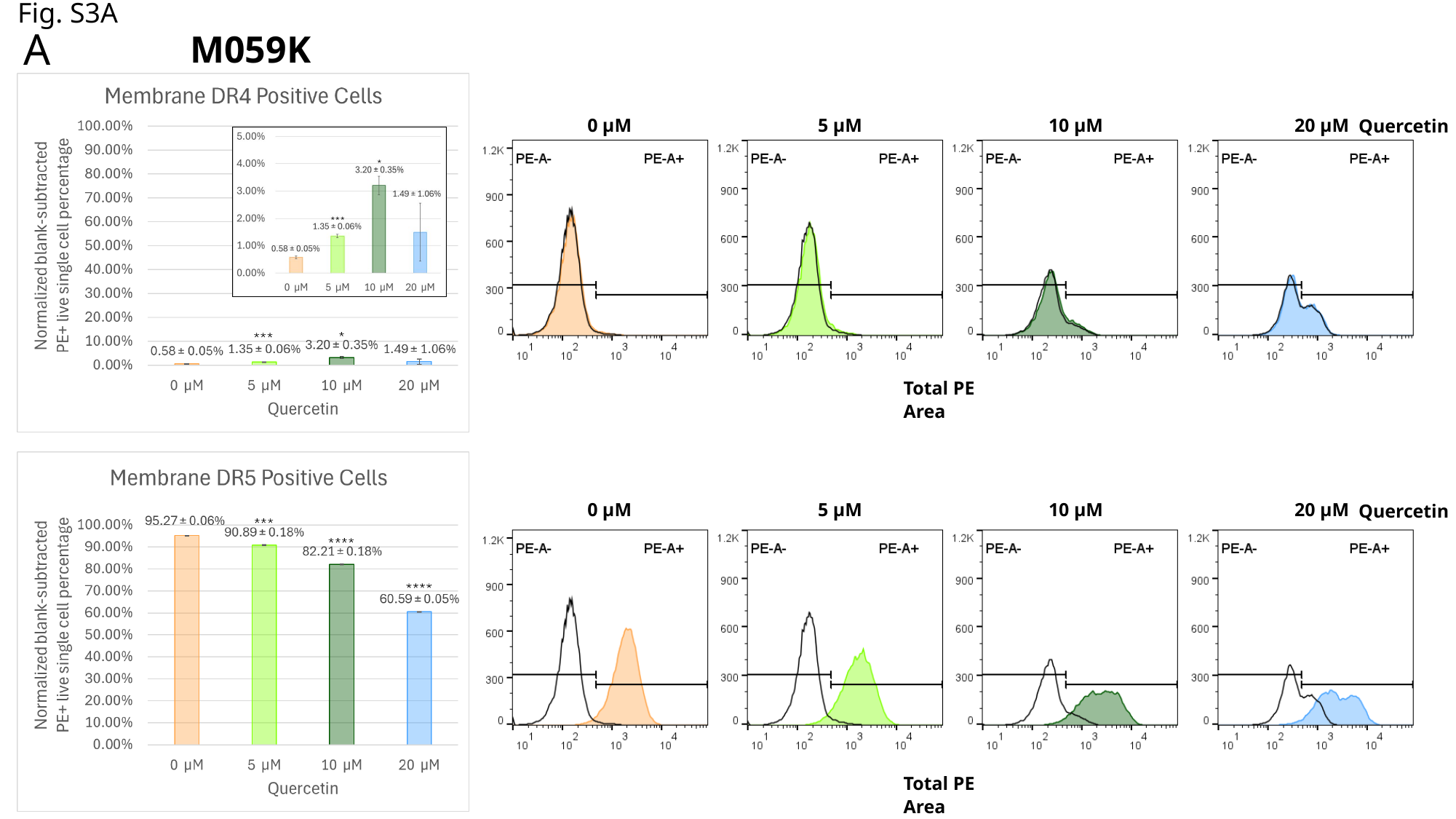

Fig. S3A
M059K
A
0 µM
5 µM
10 µM
20 µM
Quercetin
Total PE Area
0 µM
5 µM
10 µM
20 µM
Quercetin
Total PE Area

## Slide 6
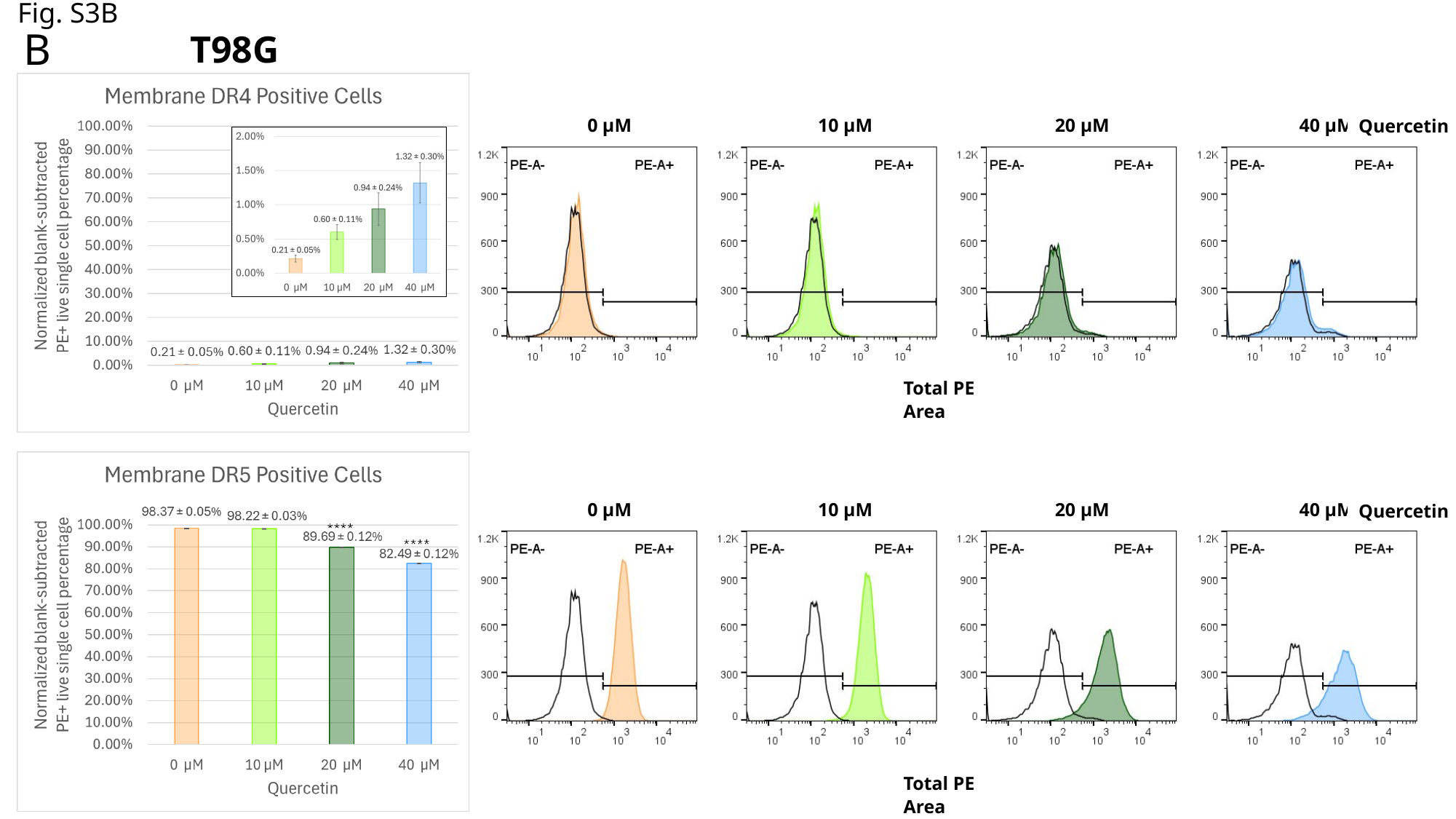

Fig. S3B
T98G
B
0 µM
10 µM
20 µM
40 µM
Quercetin
Total PE Area
0 µM
10 µM
20 µM
40 µM
Quercetin
Total PE Area

## Slide 7
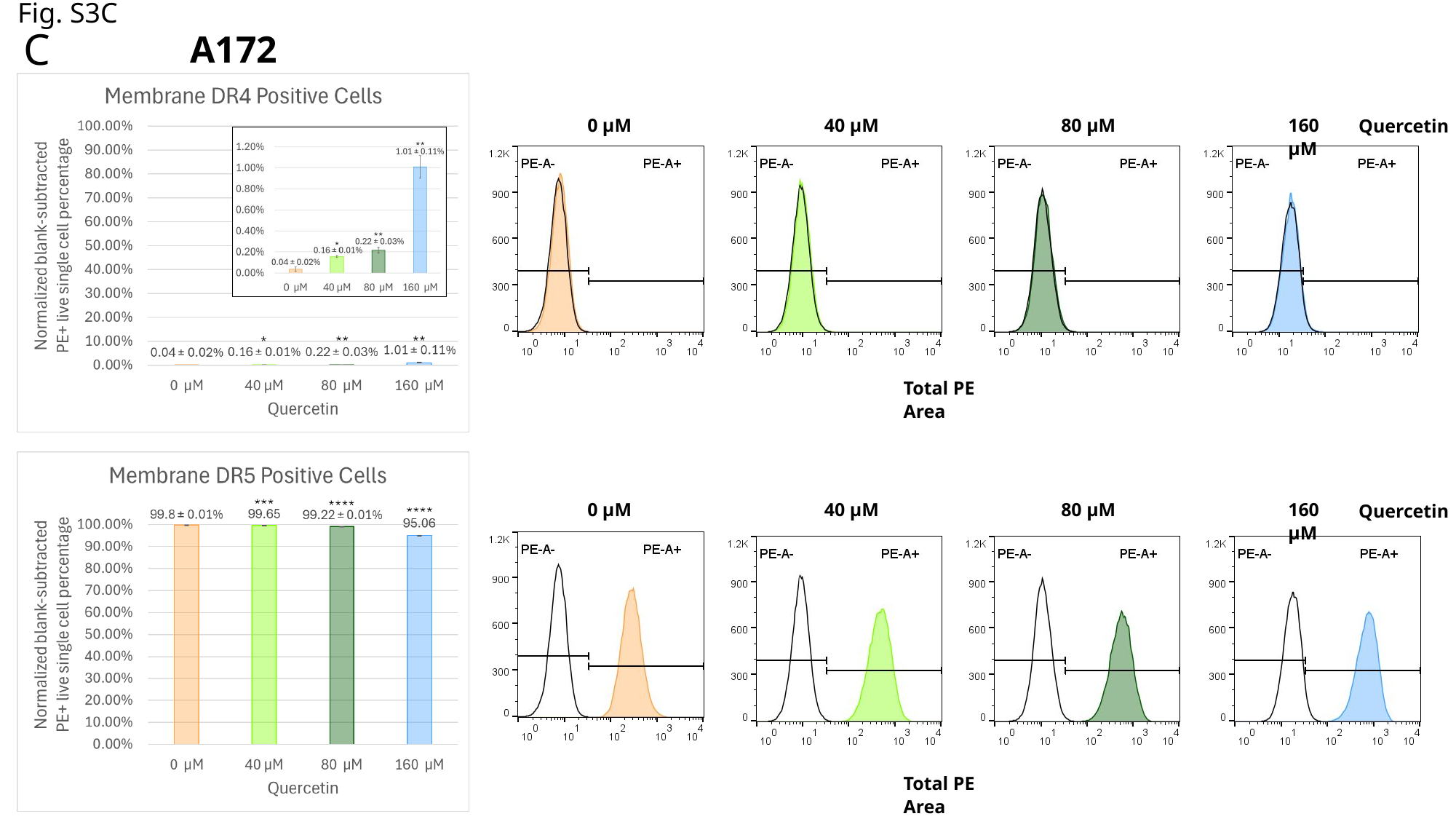

Fig. S3C
A172
C
0 µM
40 µM
80 µM
160 µM
Quercetin
Total PE Area
0 µM
40 µM
80 µM
160 µM
Quercetin
Total PE Area

## Slide 8
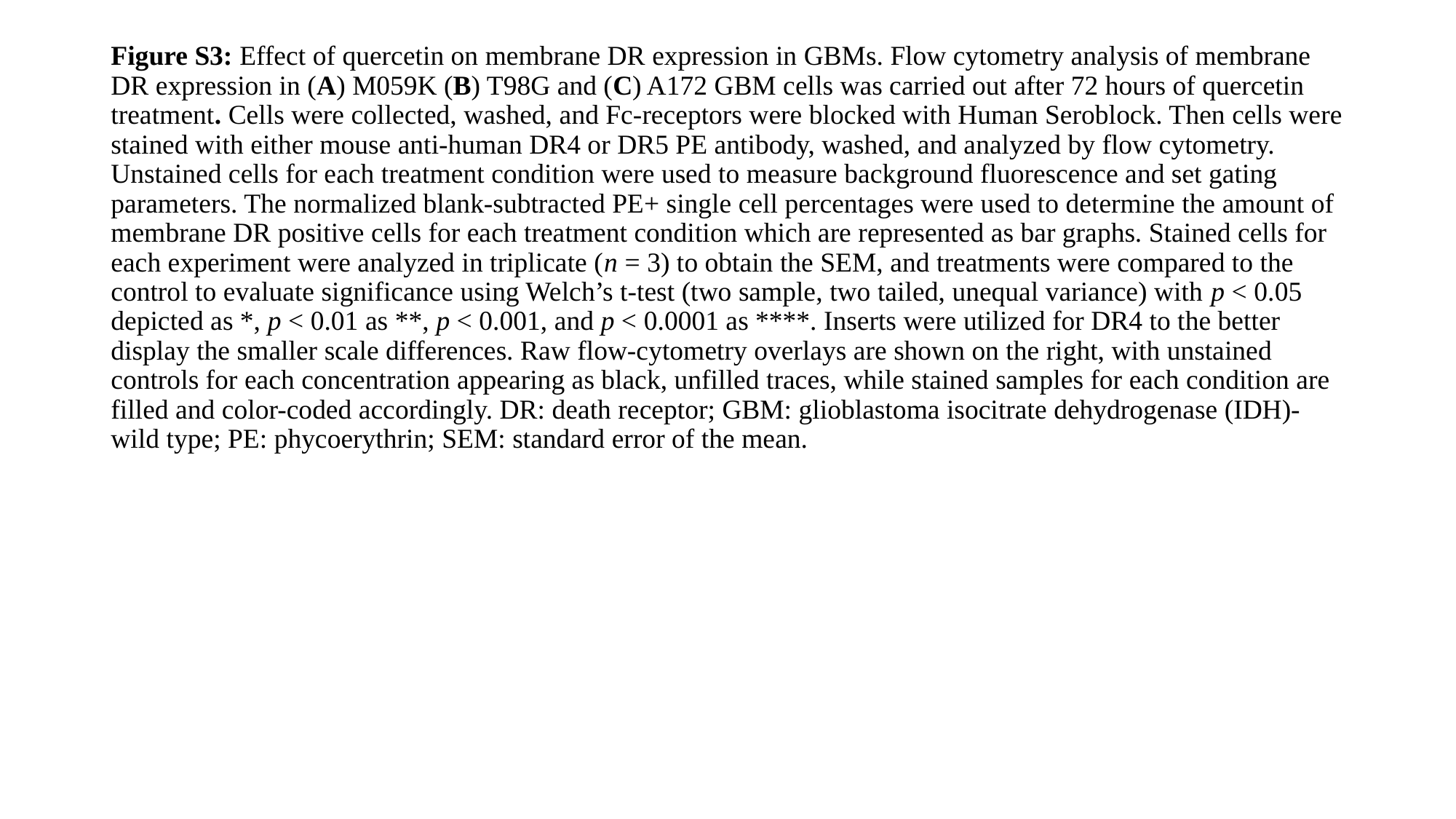

Figure S3: Effect of quercetin on membrane DR expression in GBMs. Flow cytometry analysis of membrane DR expression in (A) M059K (B) T98G and (C) A172 GBM cells was carried out after 72 hours of quercetin treatment. Cells were collected, washed, and Fc-receptors were blocked with Human Seroblock. Then cells were stained with either mouse anti-human DR4 or DR5 PE antibody, washed, and analyzed by flow cytometry. Unstained cells for each treatment condition were used to measure background fluorescence and set gating parameters. The normalized blank-subtracted PE+ single cell percentages were used to determine the amount of membrane DR positive cells for each treatment condition which are represented as bar graphs. Stained cells for each experiment were analyzed in triplicate (n = 3) to obtain the SEM, and treatments were compared to the control to evaluate significance using Welch’s t-test (two sample, two tailed, unequal variance) with p < 0.05 depicted as *, p < 0.01 as **, p < 0.001, and p < 0.0001 as ****. Inserts were utilized for DR4 to the better display the smaller scale differences. Raw flow-cytometry overlays are shown on the right, with unstained controls for each concentration appearing as black, unfilled traces, while stained samples for each condition are filled and color-coded accordingly. DR: death receptor; GBM: glioblastoma isocitrate dehydrogenase (IDH)-wild type; PE: phycoerythrin; SEM: standard error of the mean.

## Slide 9
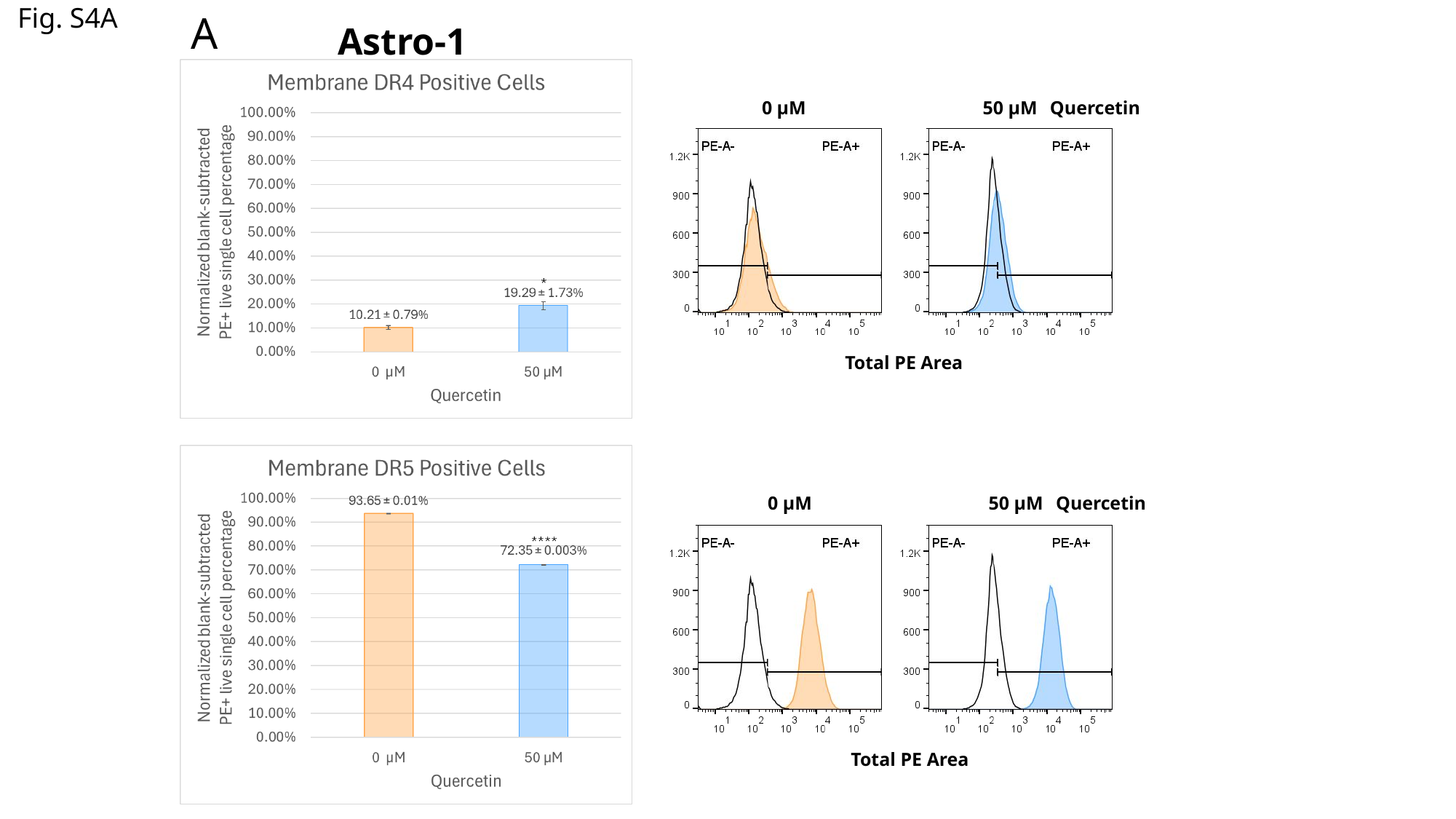

Fig. S4A
Astro-1
A
0 µM
50 µM
Quercetin
Total PE Area
0 µM
50 µM
Quercetin
Total PE Area

## Slide 10
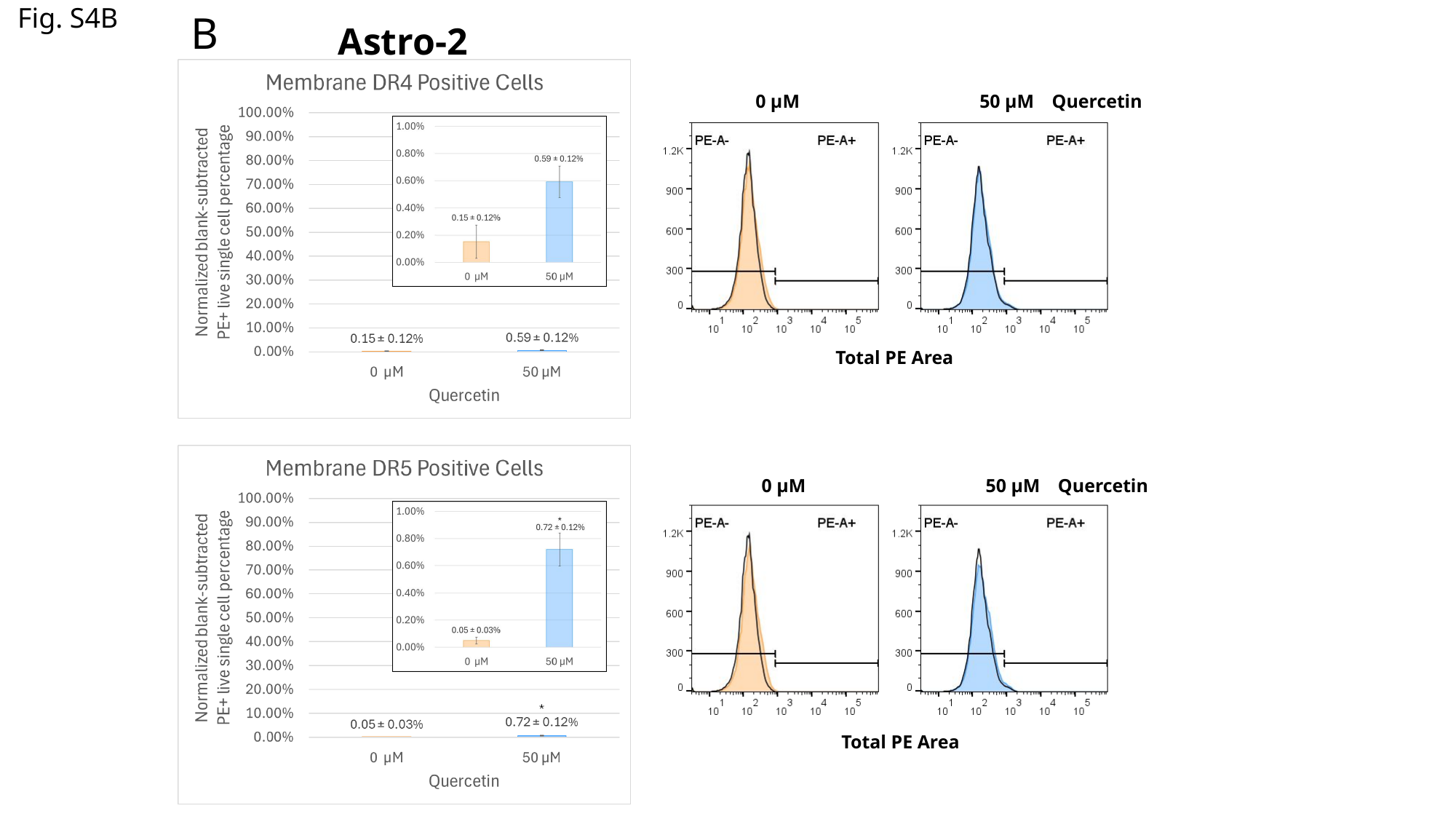

Fig. S4B
Astro-2
B
0 µM
50 µM
Quercetin
Total PE Area
0 µM
50 µM
Quercetin
Total PE Area

## Slide 11
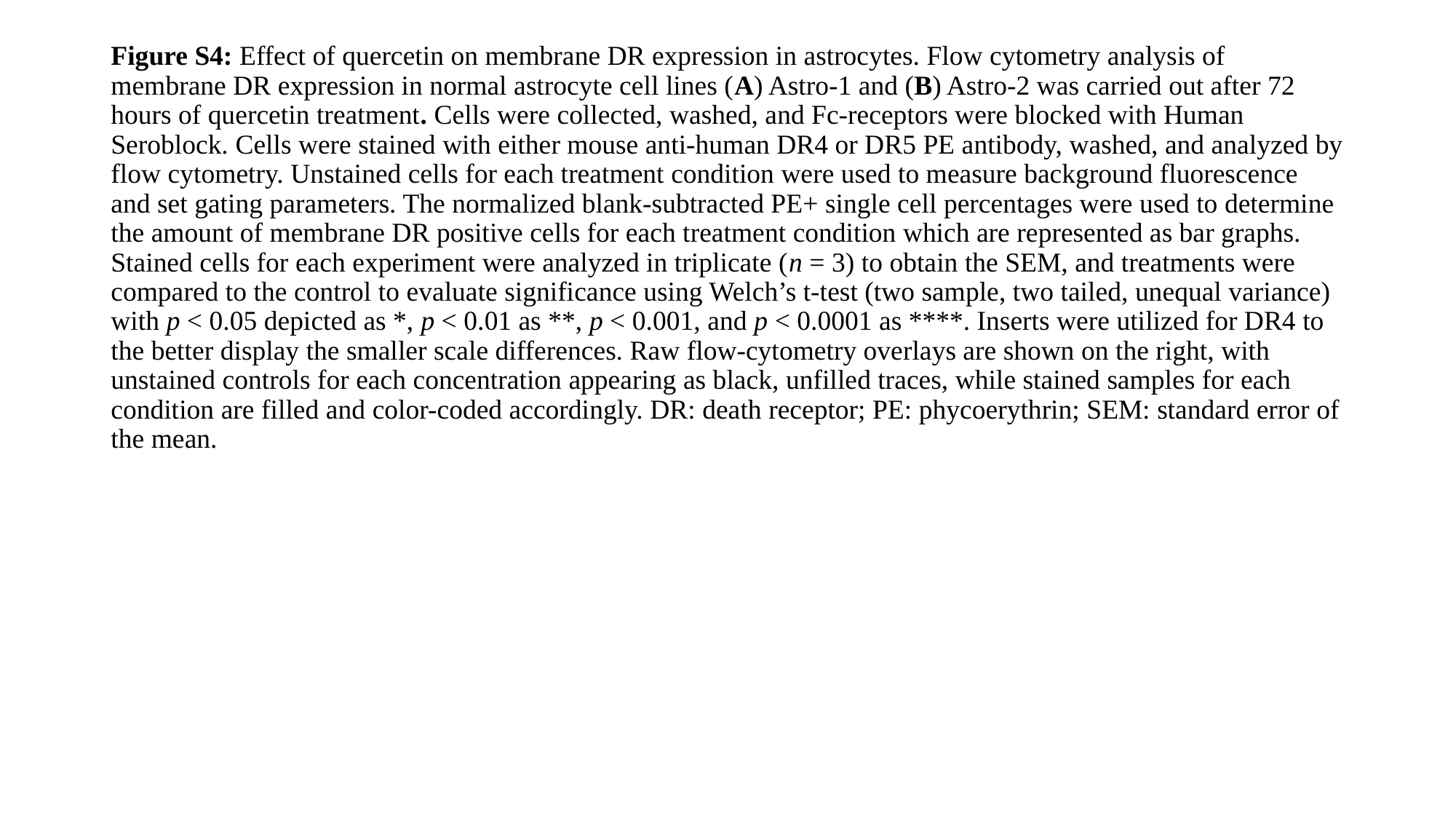

Figure S4: Effect of quercetin on membrane DR expression in astrocytes. Flow cytometry analysis of membrane DR expression in normal astrocyte cell lines (A) Astro-1 and (B) Astro-2 was carried out after 72 hours of quercetin treatment. Cells were collected, washed, and Fc-receptors were blocked with Human Seroblock. Cells were stained with either mouse anti-human DR4 or DR5 PE antibody, washed, and analyzed by flow cytometry. Unstained cells for each treatment condition were used to measure background fluorescence and set gating parameters. The normalized blank-subtracted PE+ single cell percentages were used to determine the amount of membrane DR positive cells for each treatment condition which are represented as bar graphs. Stained cells for each experiment were analyzed in triplicate (n = 3) to obtain the SEM, and treatments were compared to the control to evaluate significance using Welch’s t-test (two sample, two tailed, unequal variance) with p < 0.05 depicted as *, p < 0.01 as **, p < 0.001, and p < 0.0001 as ****. Inserts were utilized for DR4 to the better display the smaller scale differences. Raw flow-cytometry overlays are shown on the right, with unstained controls for each concentration appearing as black, unfilled traces, while stained samples for each condition are filled and color-coded accordingly. DR: death receptor; PE: phycoerythrin; SEM: standard error of the mean.
