# Supplementary figures and images for: Quercetin Increases Expression of Membrane-TRAIL in Glioblastoma Cells Resulting in Apoptosis
Source: Cancers (Basel). 2025 Sep 30;17(19):3197. doi: 10.3390/cancers17193197 (PMC12523679; doi:10.3390/cancers17193197)

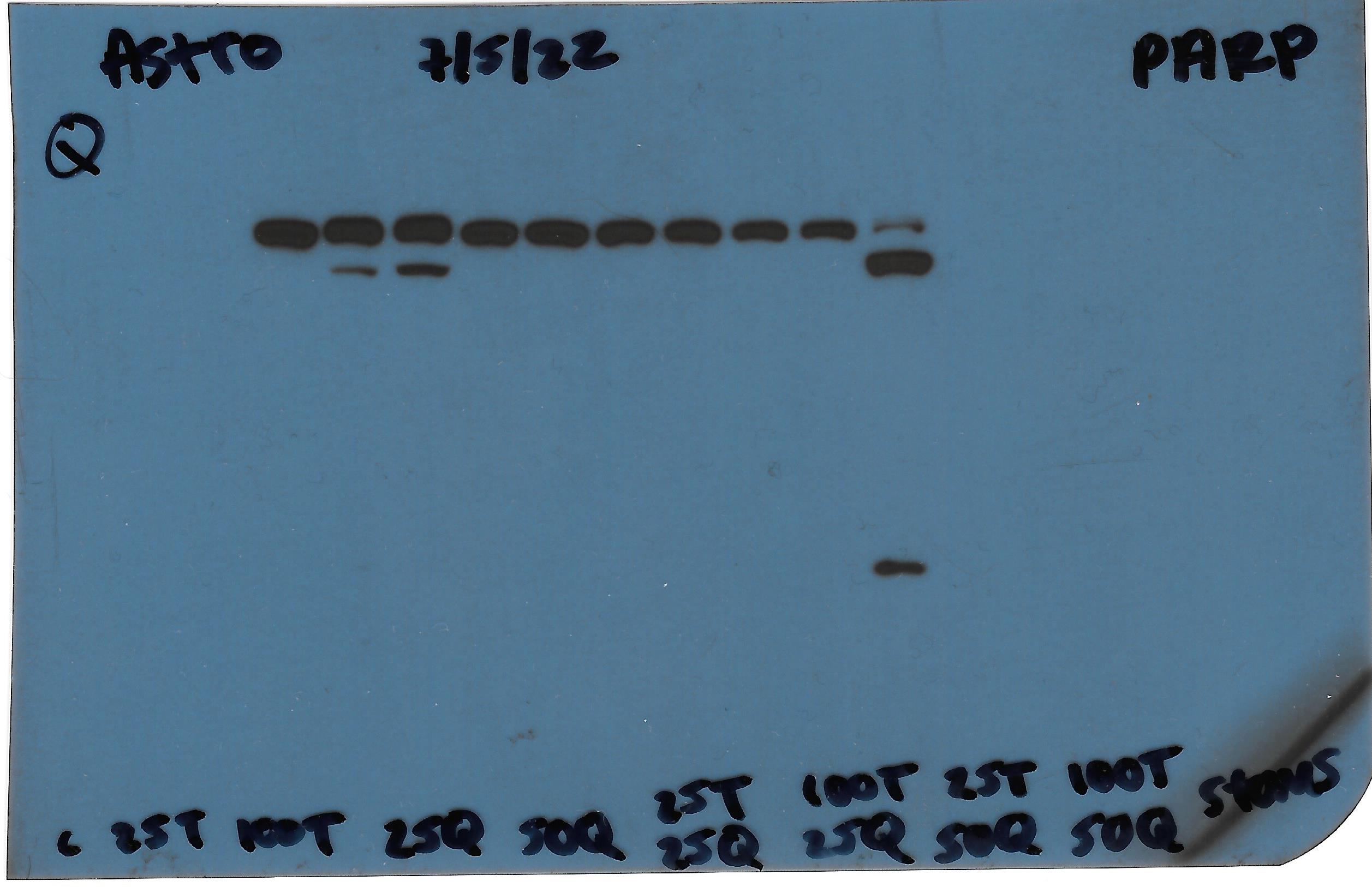

Supplement: Supplementary file 1 [file cancers-17-03197-s001.zip › OriginalBlots/Figure1A-Astro1/2022-07-05_Astro_Q+TRAIL_PARP1.jpg]

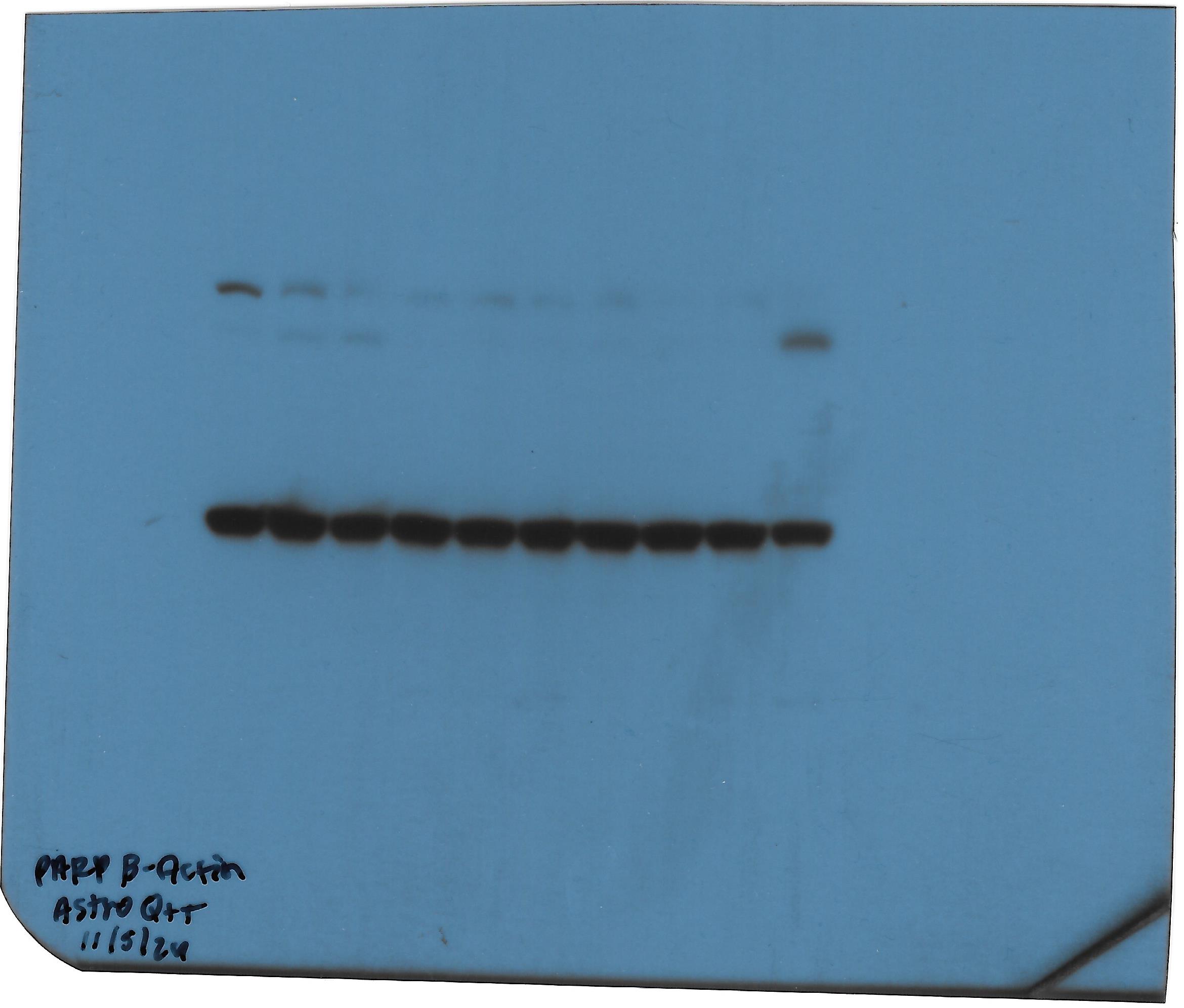

Supplement: Supplementary file 1 [file cancers-17-03197-s001.zip › OriginalBlots/Figure1A-Astro1/2024-11-05_Astro_Q+T_PARP_Actin_2.jpg]

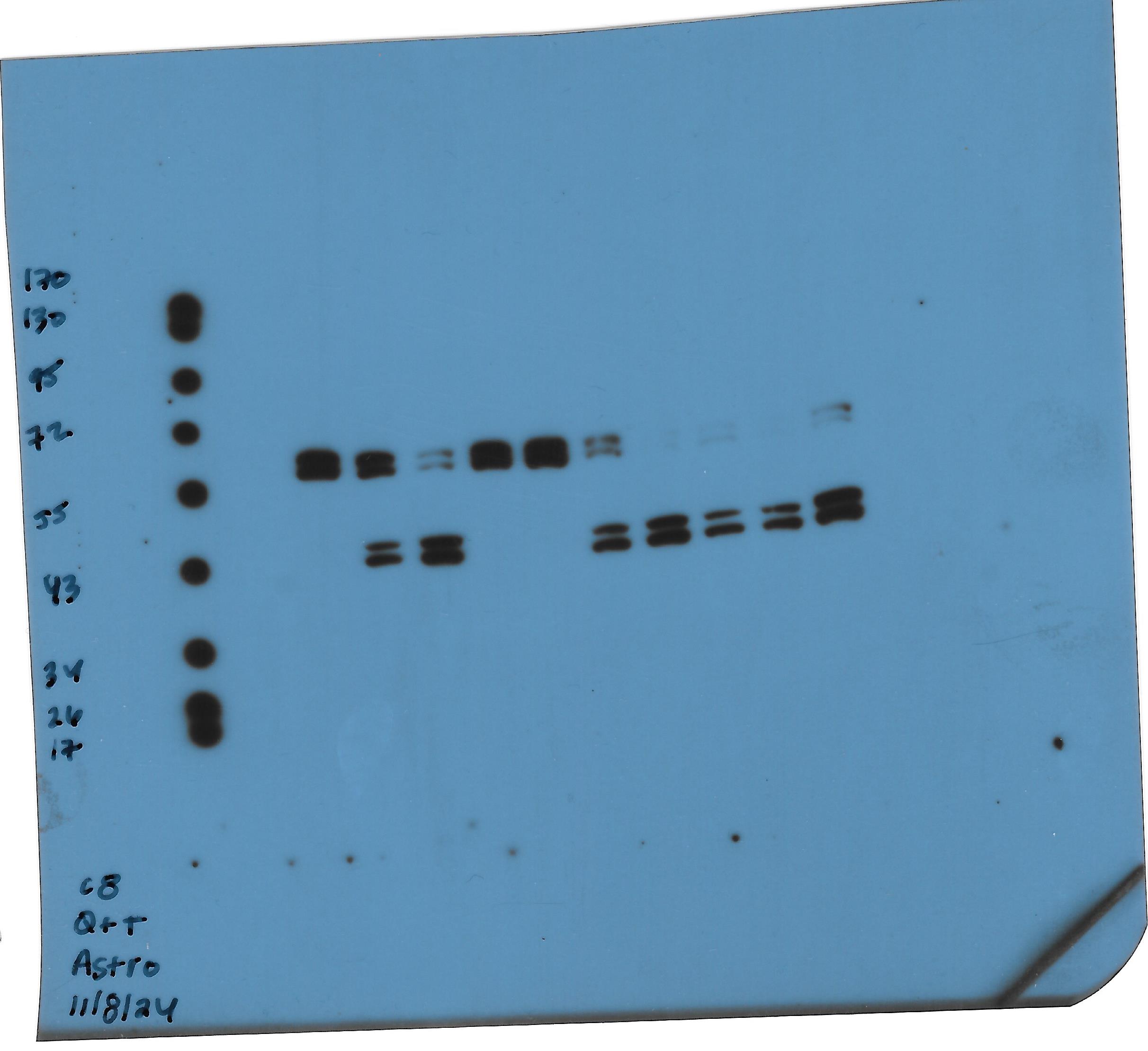

Supplement: Supplementary file 1 [file cancers-17-03197-s001.zip › OriginalBlots/Figure1A-Astro1/2024-11-09_Astro_Q+T_C8_5.jpg]

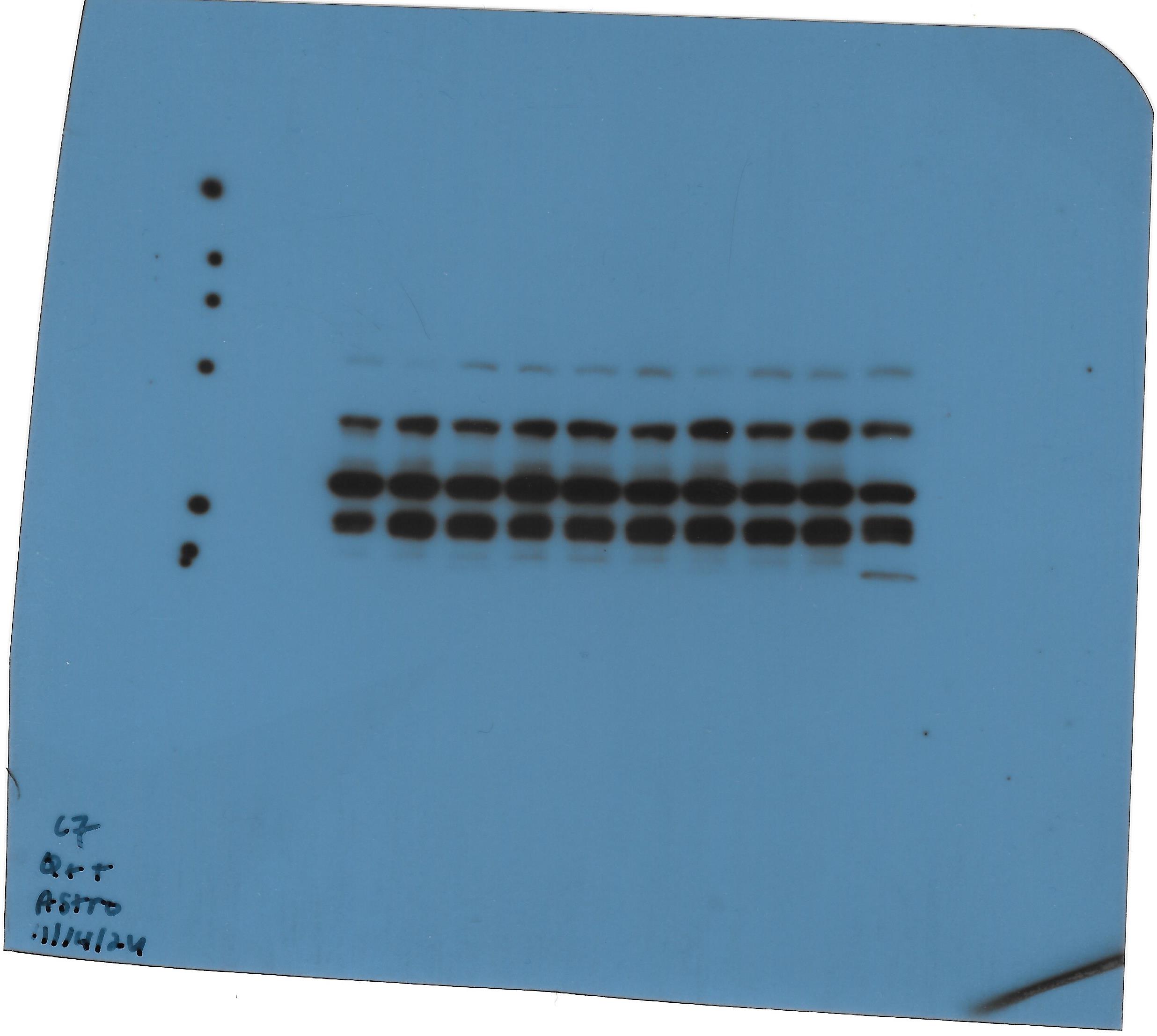

Supplement: Supplementary file 1 [file cancers-17-03197-s001.zip › OriginalBlots/Figure1A-Astro1/2024-11-14_Astro_Q+T_C7_10.jpg]

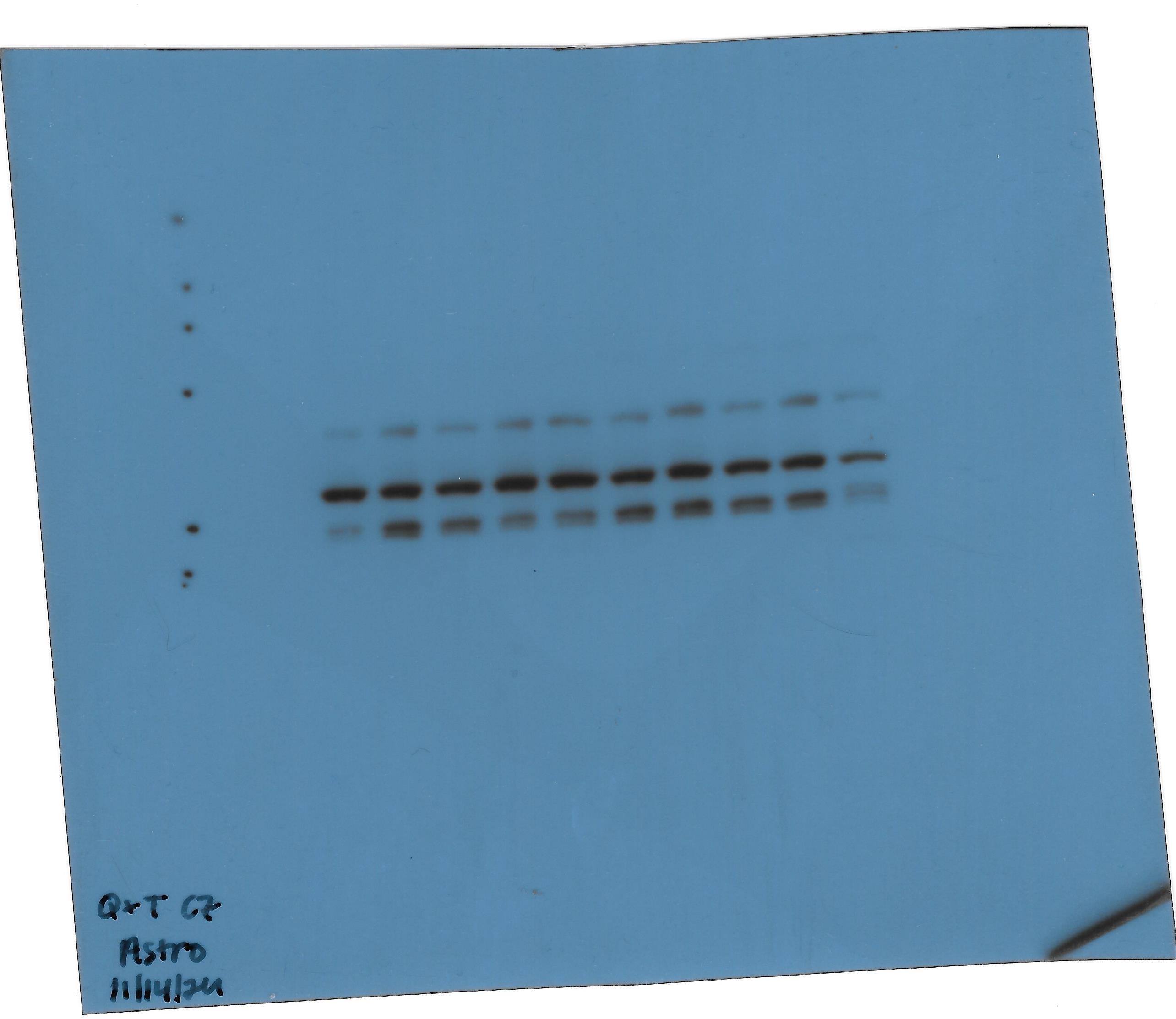

Supplement: Supplementary file 1 [file cancers-17-03197-s001.zip › OriginalBlots/Figure1A-Astro1/2024-11-14_Astro_Q+T_C7_6.jpg]

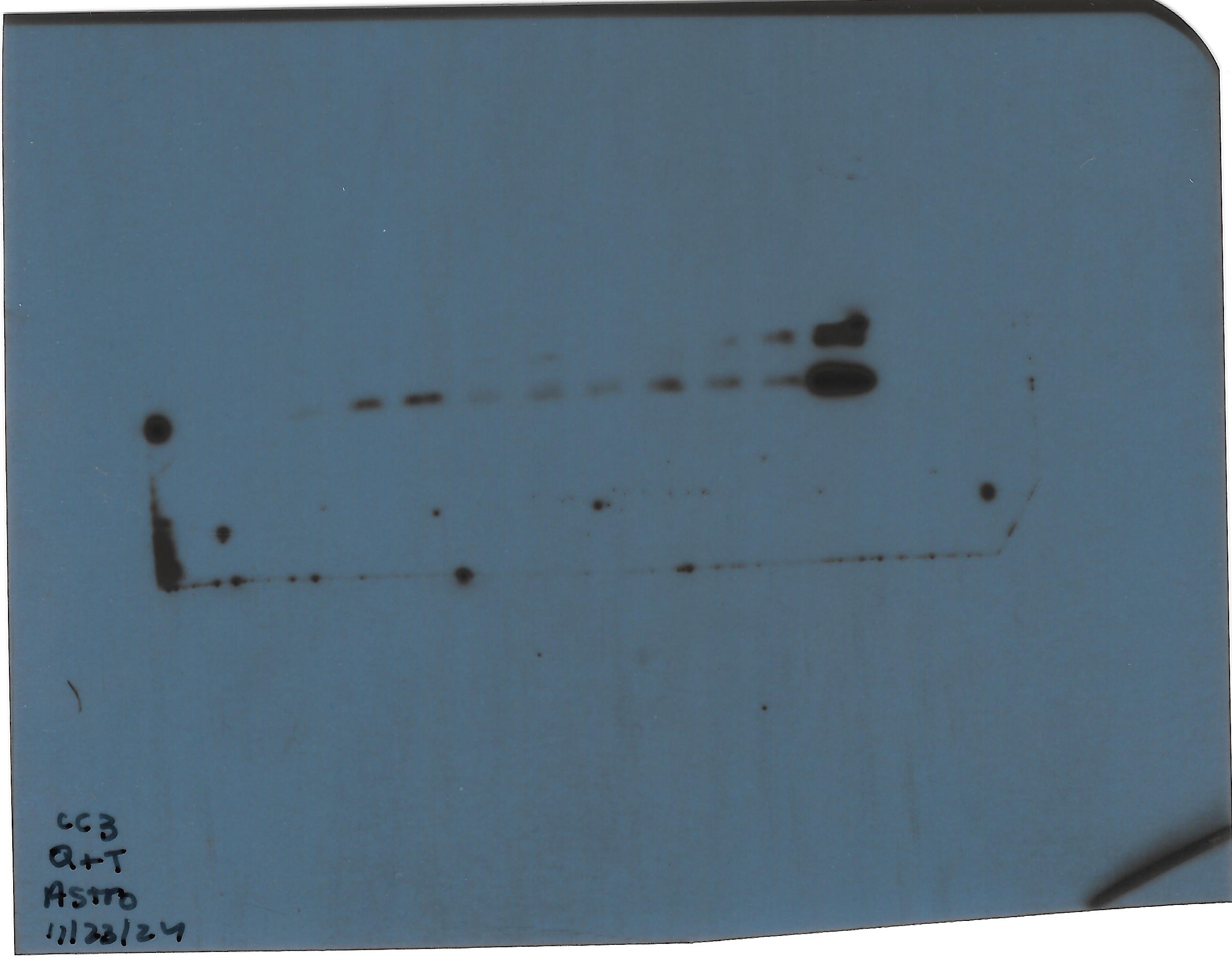

Supplement: Supplementary file 1 [file cancers-17-03197-s001.zip › OriginalBlots/Figure1A-Astro1/2024-11-23_Astro_Q+T_CC3_2.jpg]

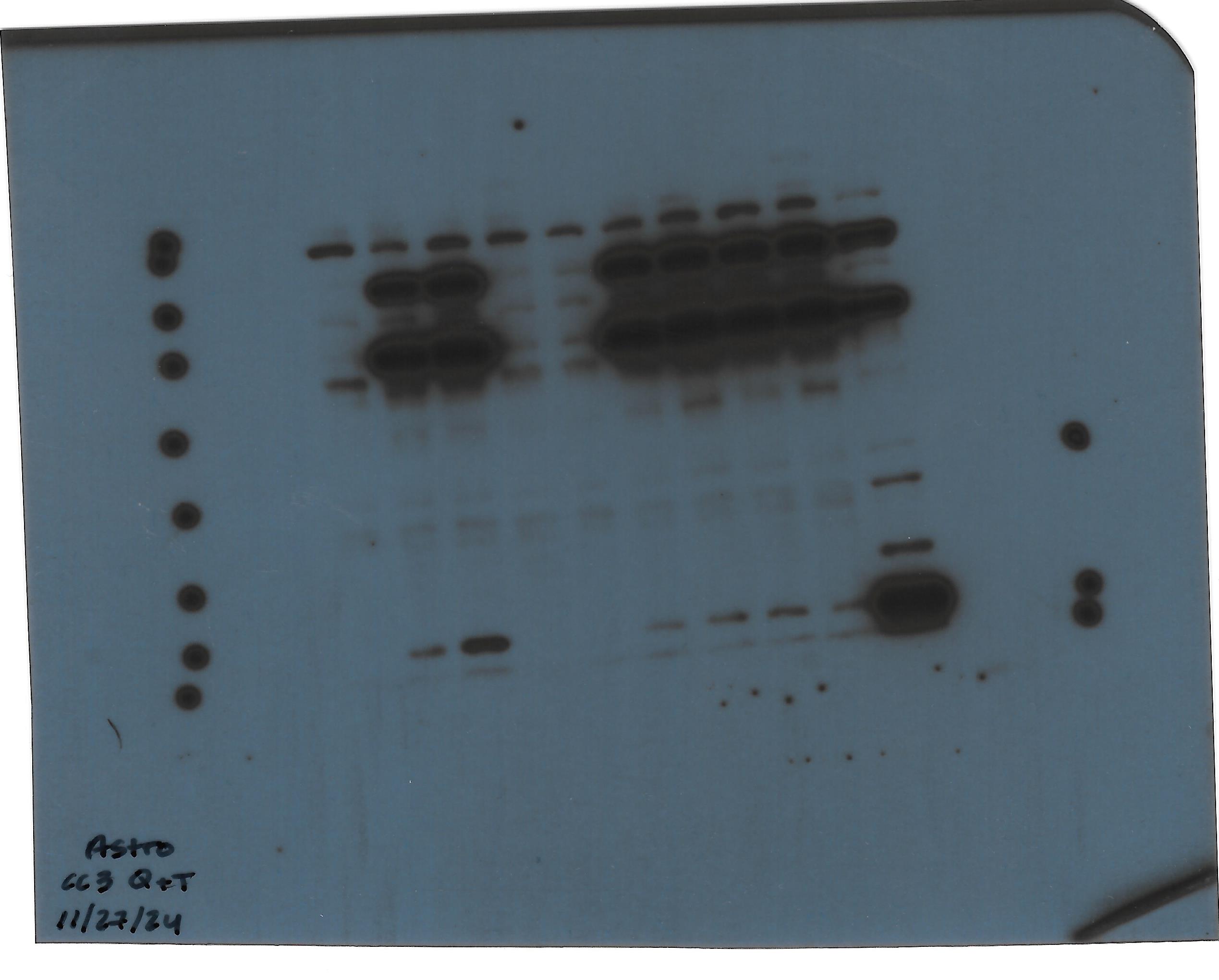

Supplement: Supplementary file 1 [file cancers-17-03197-s001.zip › OriginalBlots/Figure1A-Astro1/2024-11-27_Astro_Q+T_CC3_1.jpg]

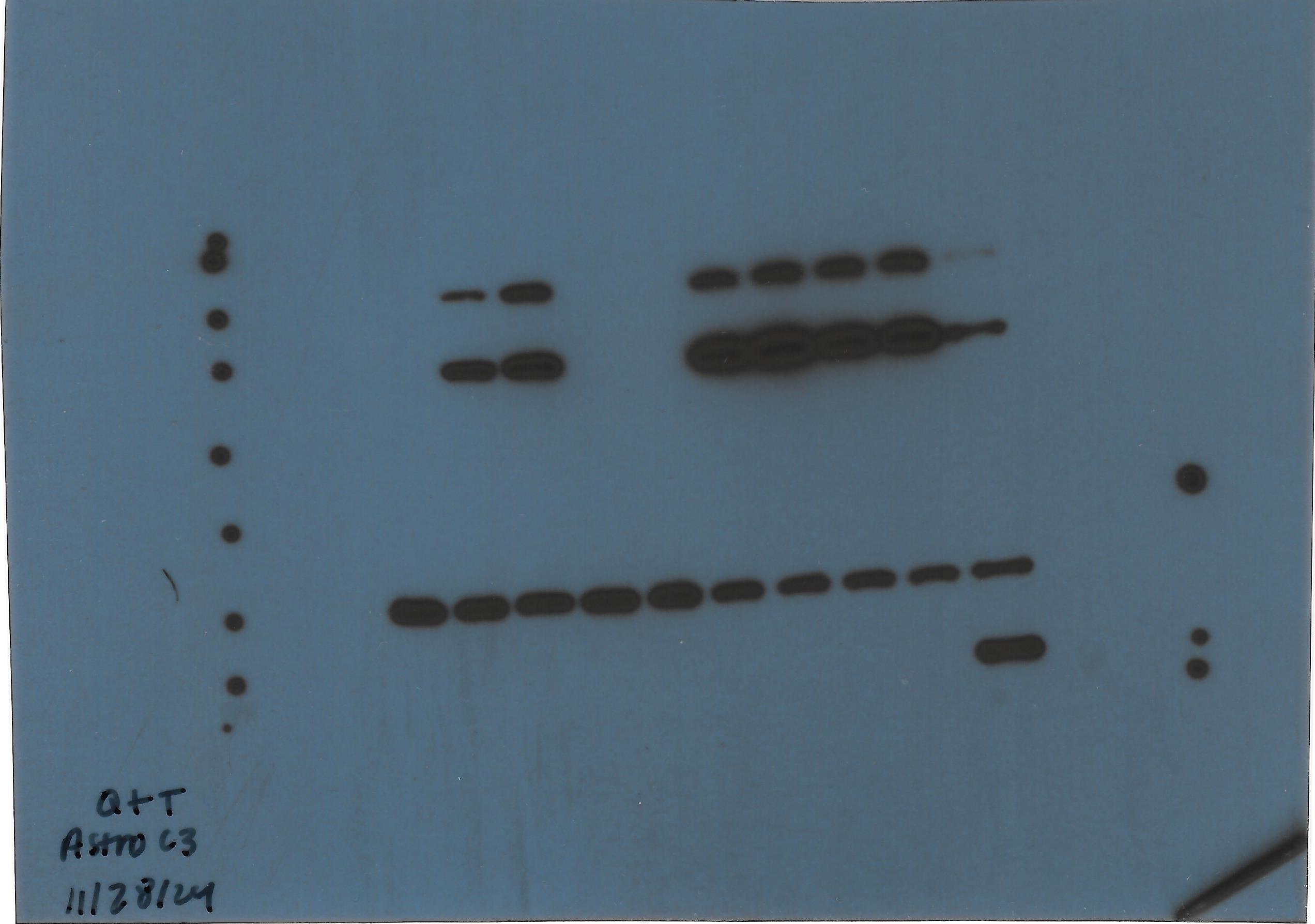

Supplement: Supplementary file 1 [file cancers-17-03197-s001.zip › OriginalBlots/Figure1A-Astro1/2024-11-28_Astro_Q+T_C3_4.jpg]

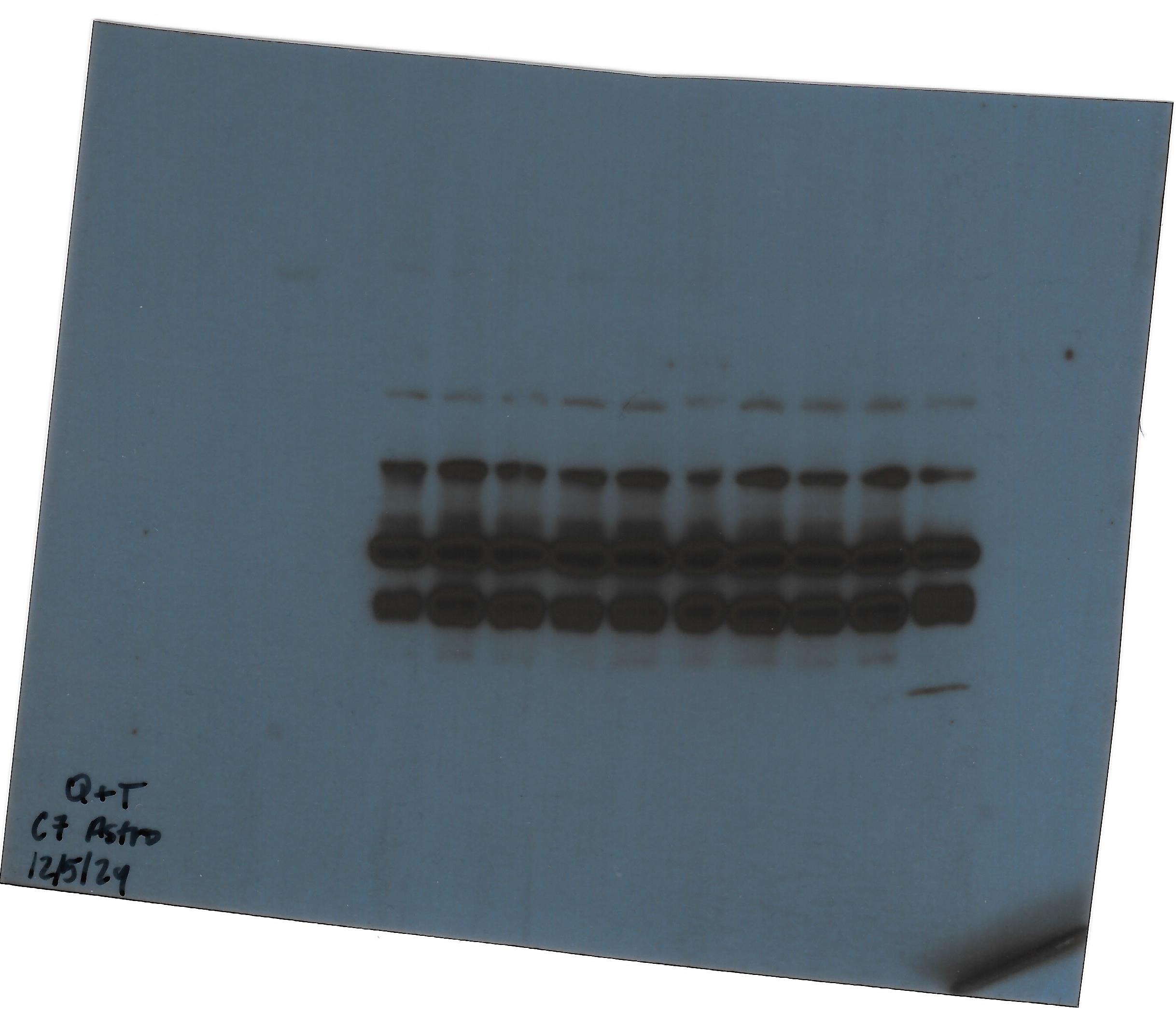

Supplement: Supplementary file 1 [file cancers-17-03197-s001.zip › OriginalBlots/Figure1A-Astro1/2024-12-05_Astro_Q+T_C7_1.jpg]

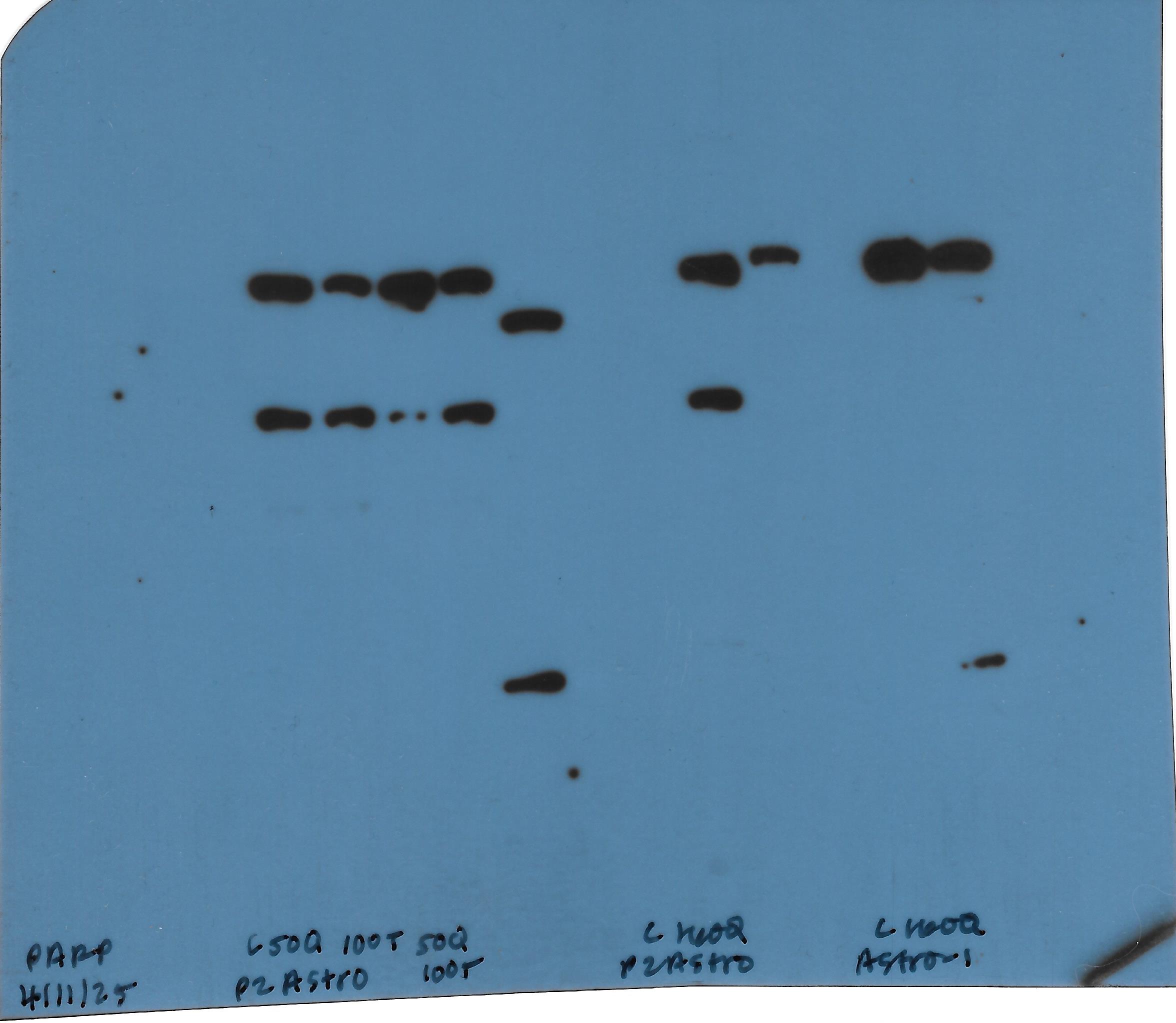

Supplement: Supplementary file 1 [file cancers-17-03197-s001.zip › OriginalBlots/Figure1B-Astro2/2025-04-11_Astro_Q+T_PARP_4.jpg]

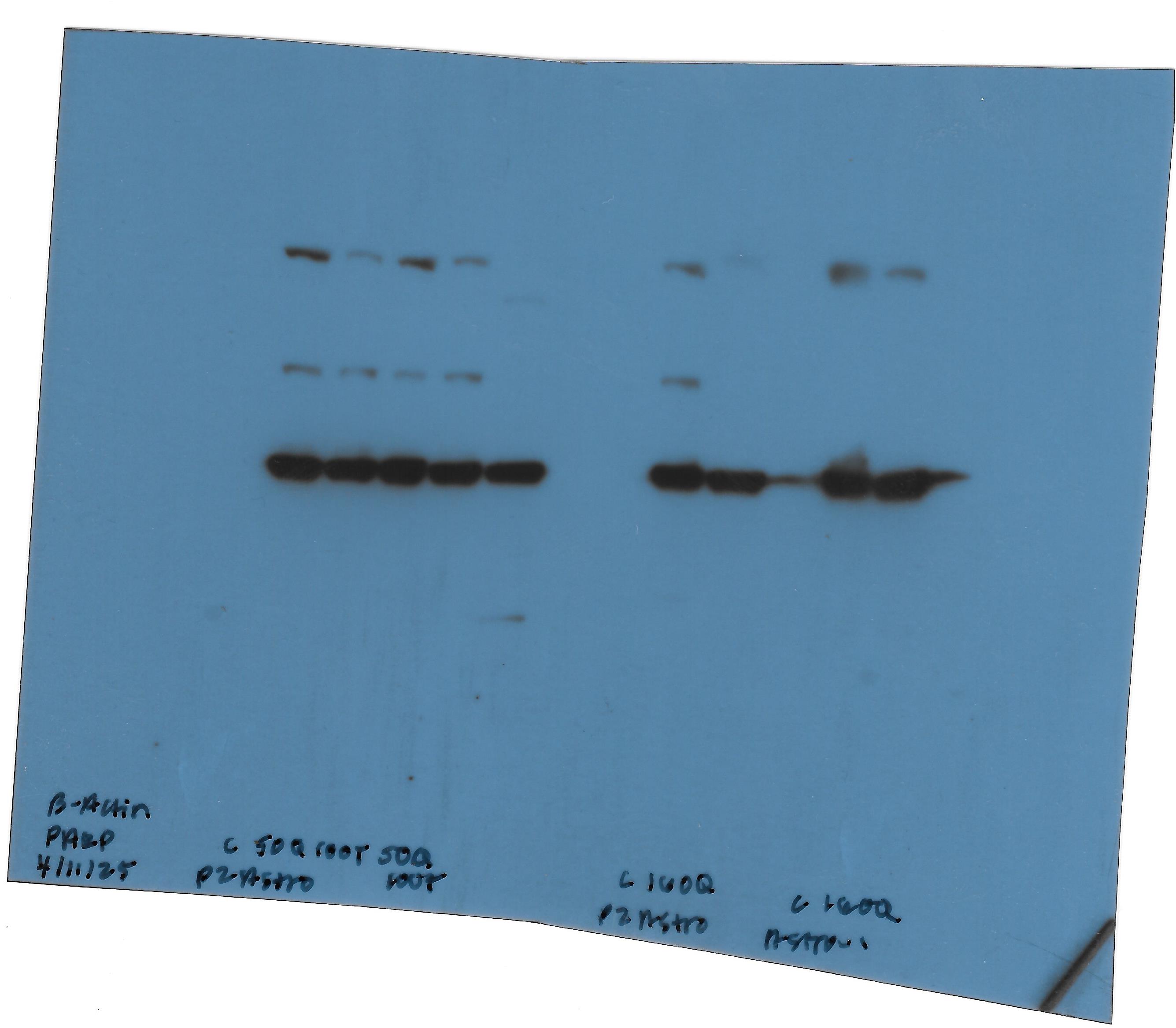

Supplement: Supplementary file 1 [file cancers-17-03197-s001.zip › OriginalBlots/Figure1B-Astro2/2025-04-11_Astro_Q+T_PARP_Actin_1.jpg]

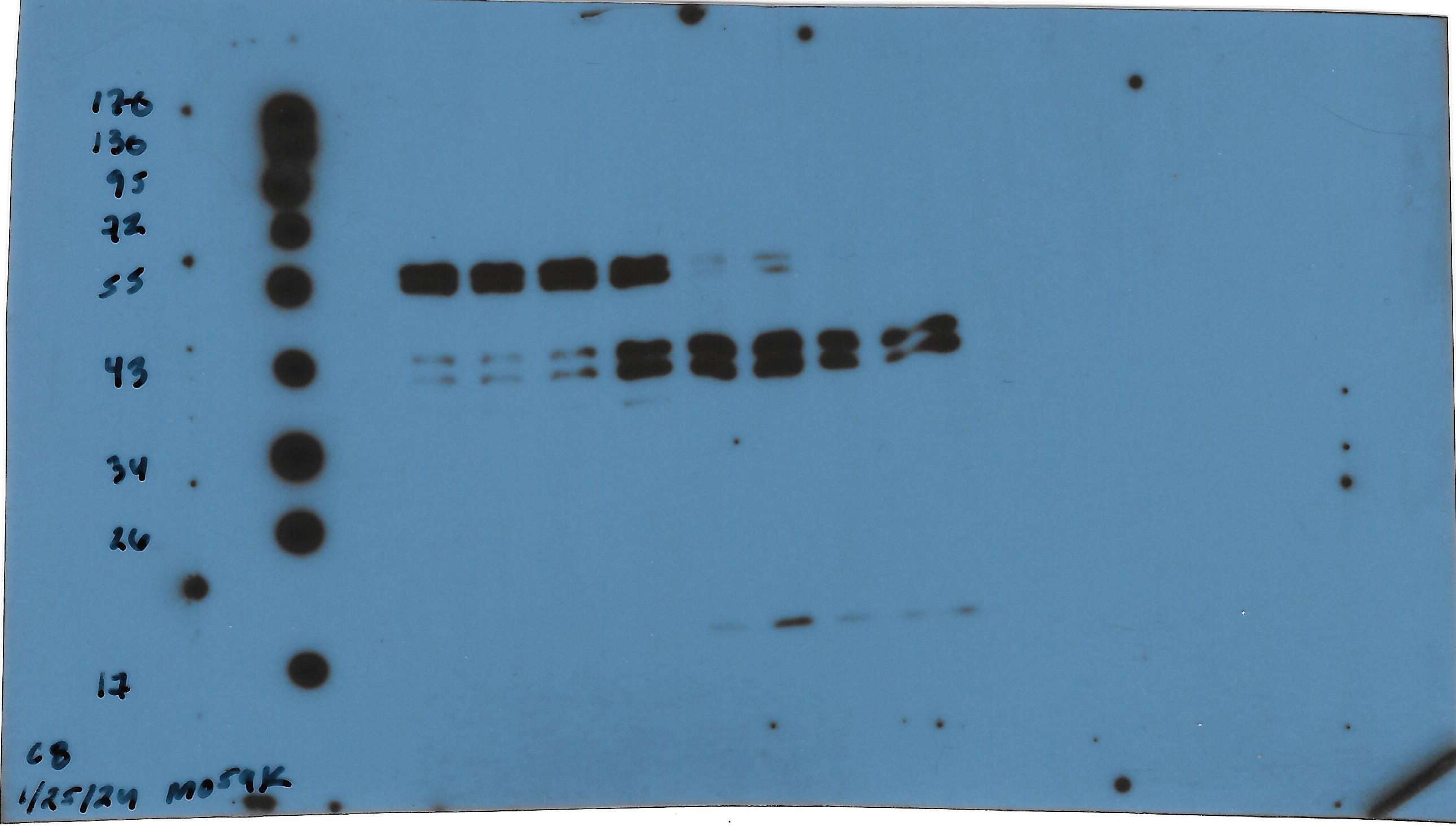

Supplement: Supplementary file 1 [file cancers-17-03197-s001.zip › OriginalBlots/Figure2A-M059K/2024-01-25_M059K_C8_1.jpg]

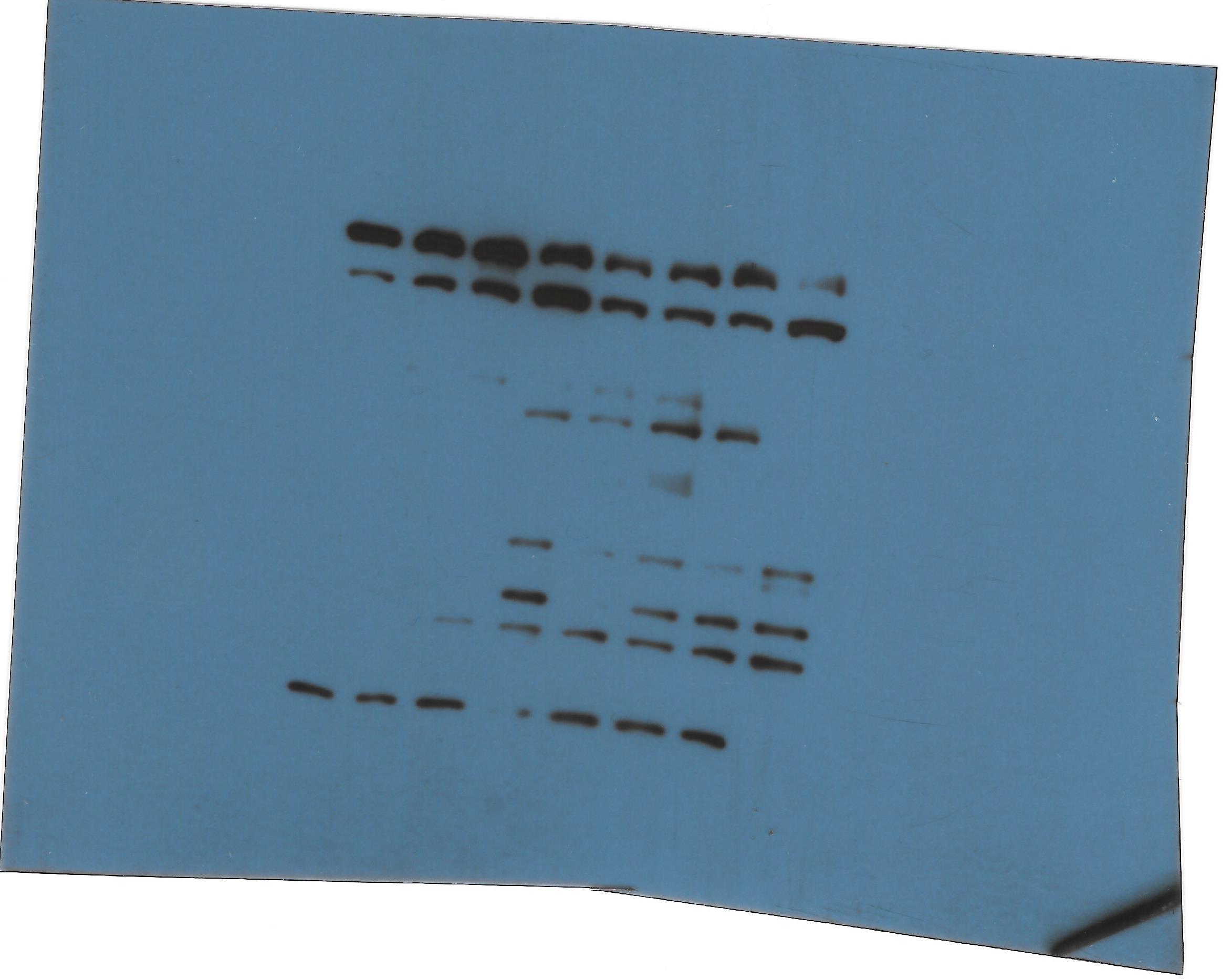

Supplement: Supplementary file 1 [file cancers-17-03197-s001.zip › OriginalBlots/Figure2A-M059K/2024-01-26_M059K_PARP1.jpg]

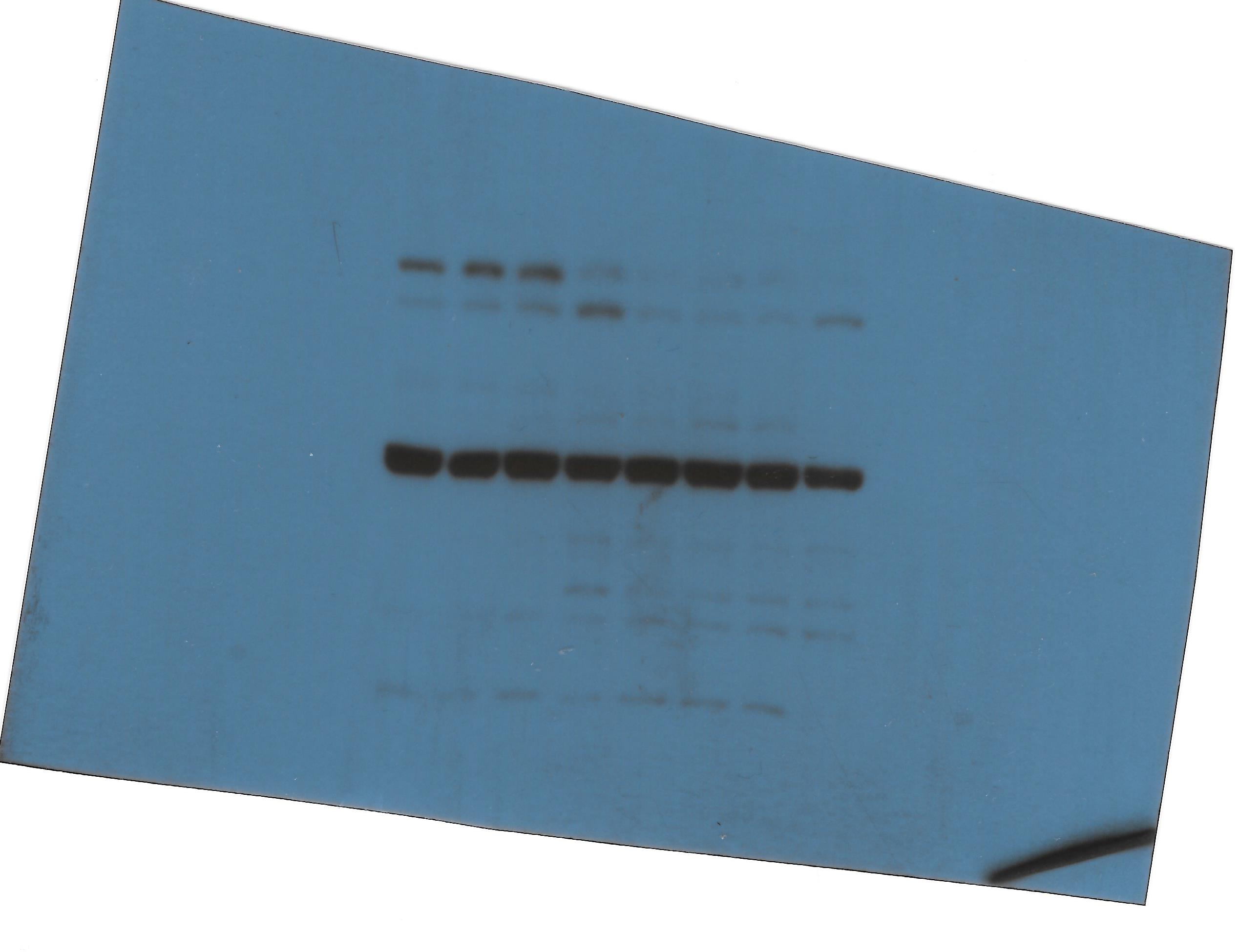

Supplement: Supplementary file 1 [file cancers-17-03197-s001.zip › OriginalBlots/Figure2A-M059K/2024-01-26_M059K_PARP3_Actin.jpg]

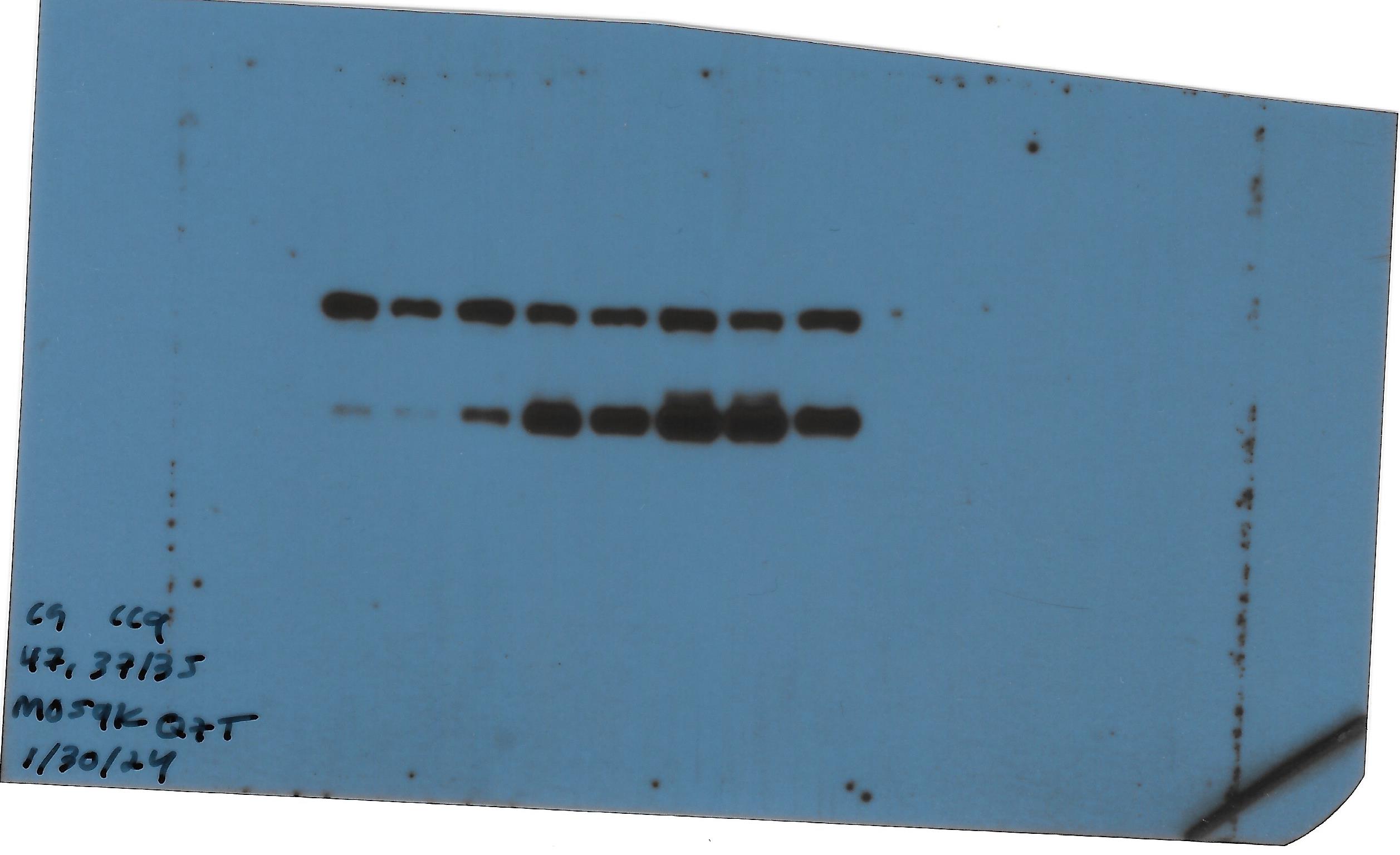

Supplement: Supplementary file 1 [file cancers-17-03197-s001.zip › OriginalBlots/Figure2A-M059K/2024-01-30_M059K_C9&CC9_1.jpg]

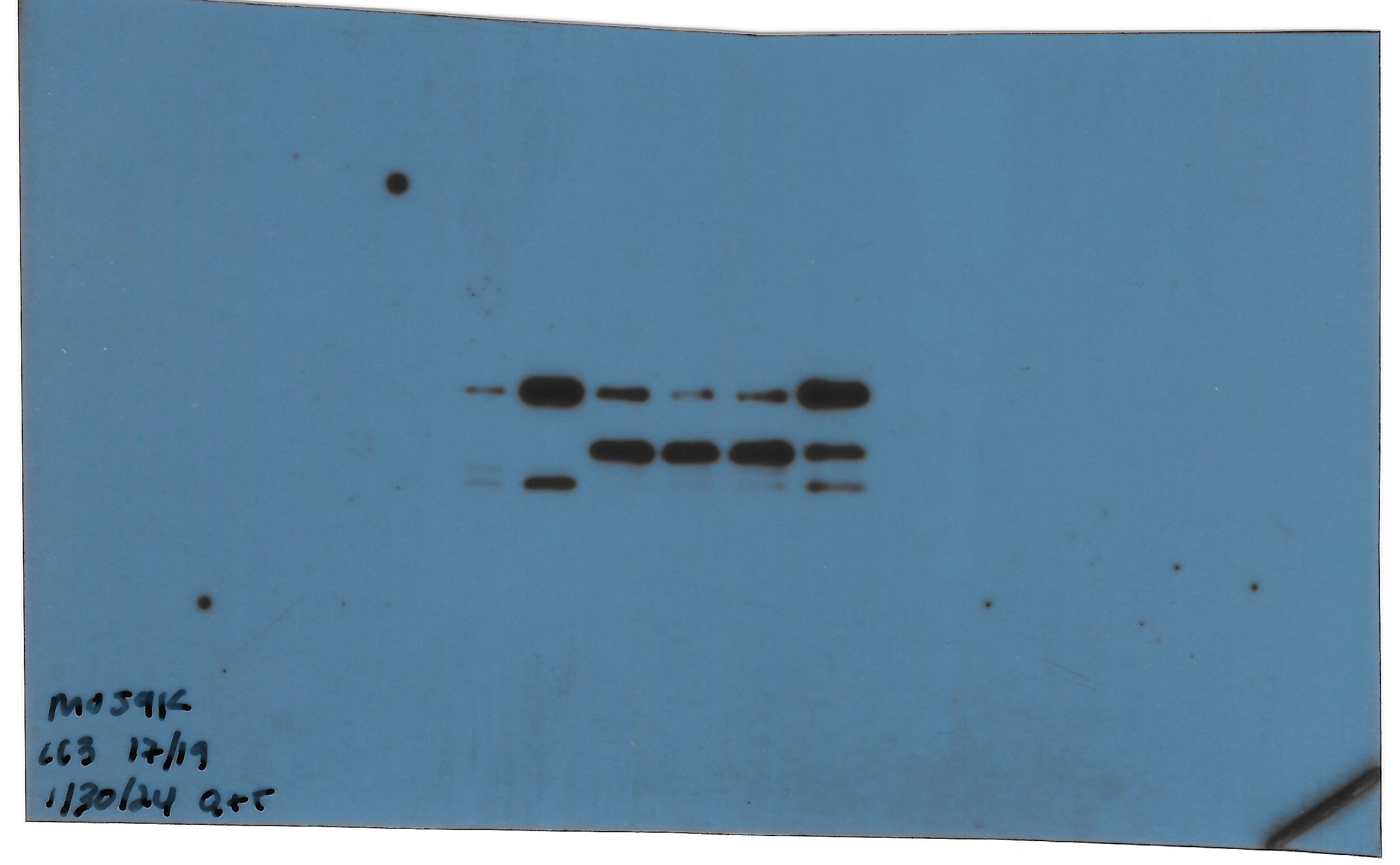

Supplement: Supplementary file 1 [file cancers-17-03197-s001.zip › OriginalBlots/Figure2A-M059K/2024-01-30_M059K_CC3_2.jpg]

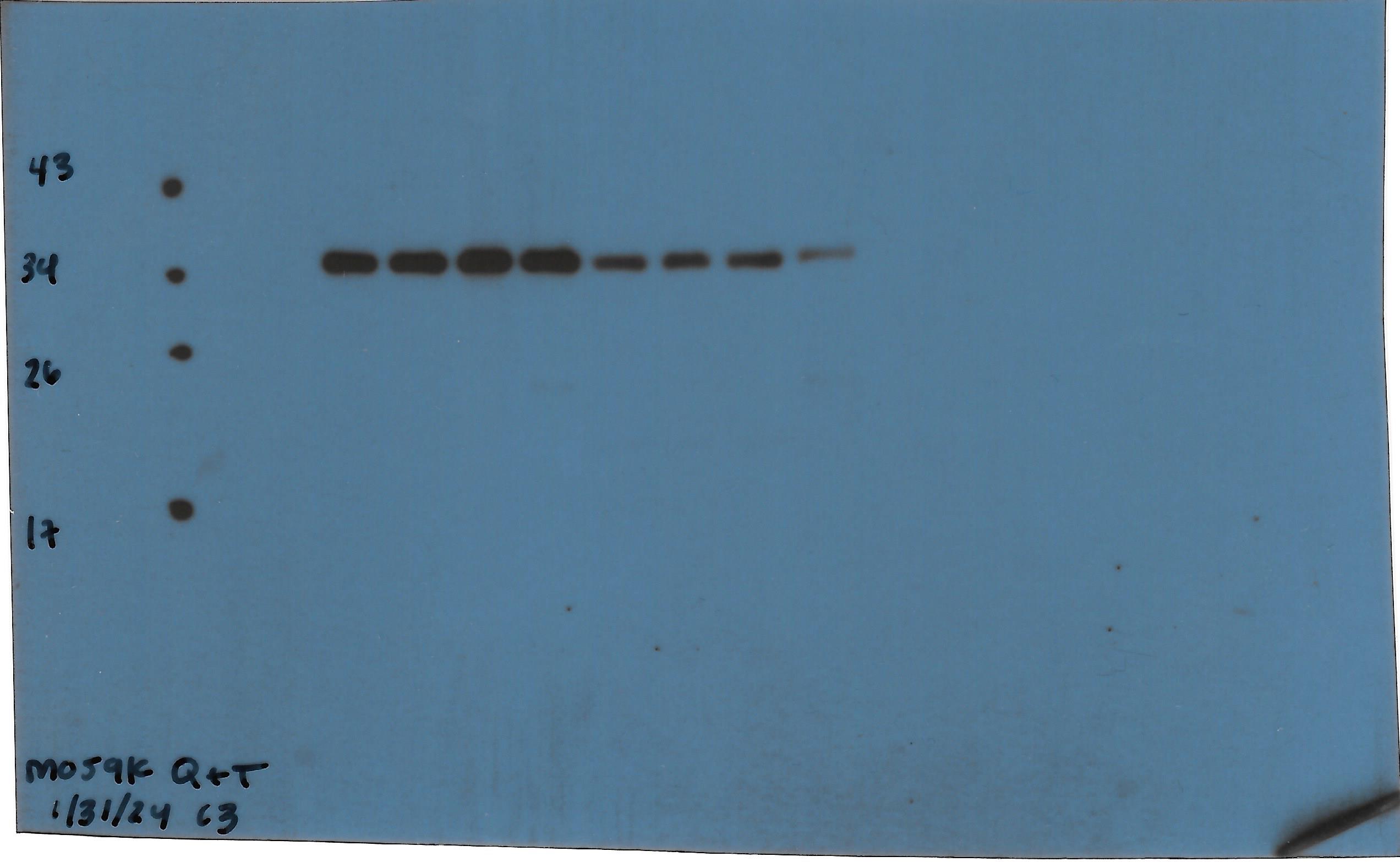

Supplement: Supplementary file 1 [file cancers-17-03197-s001.zip › OriginalBlots/Figure2A-M059K/2024-01-31_M059K_C3_1.jpg]

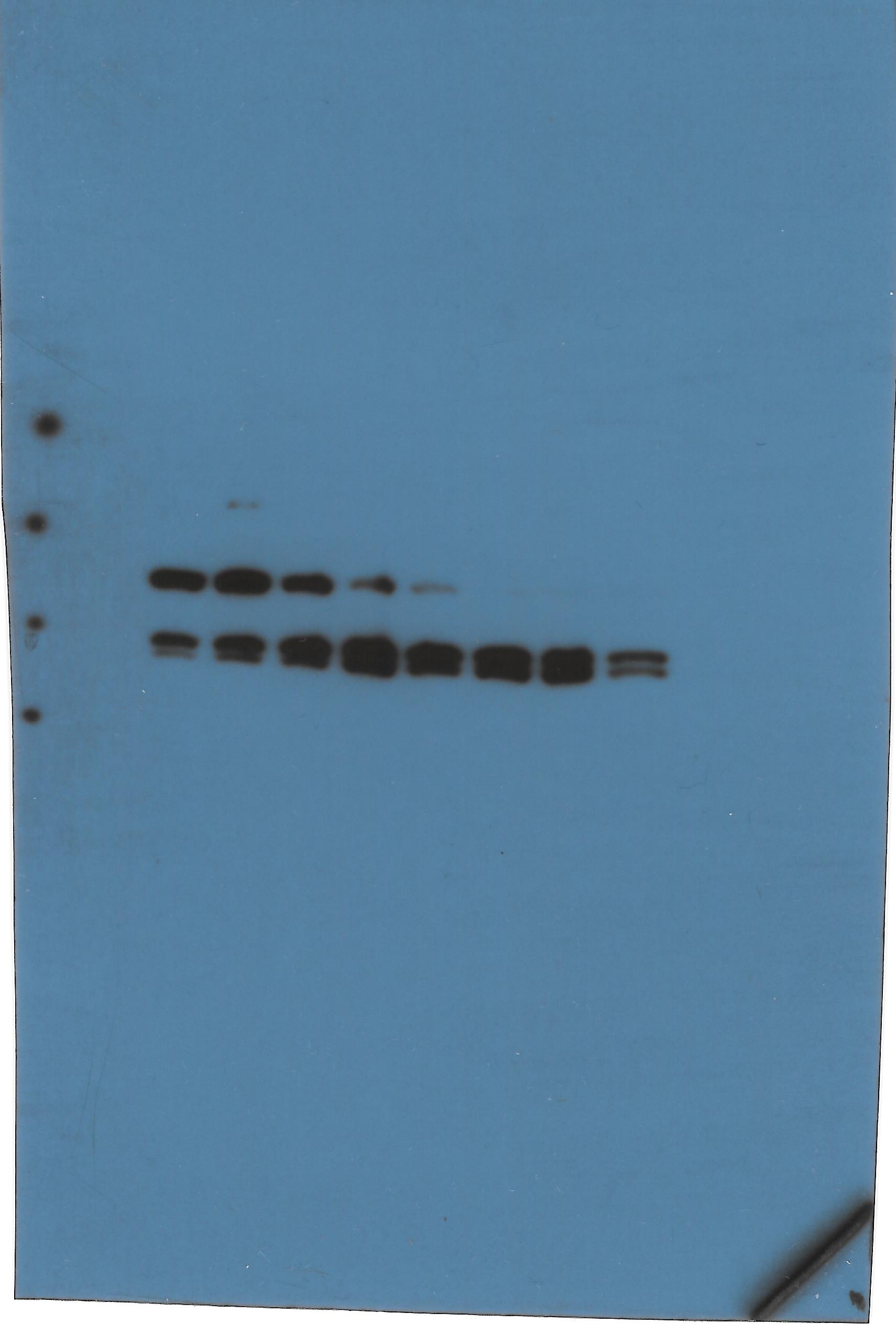

Supplement: Supplementary file 1 [file cancers-17-03197-s001.zip › OriginalBlots/Figure2A-M059K/2024-02-02_M059K_C7_2.jpg]

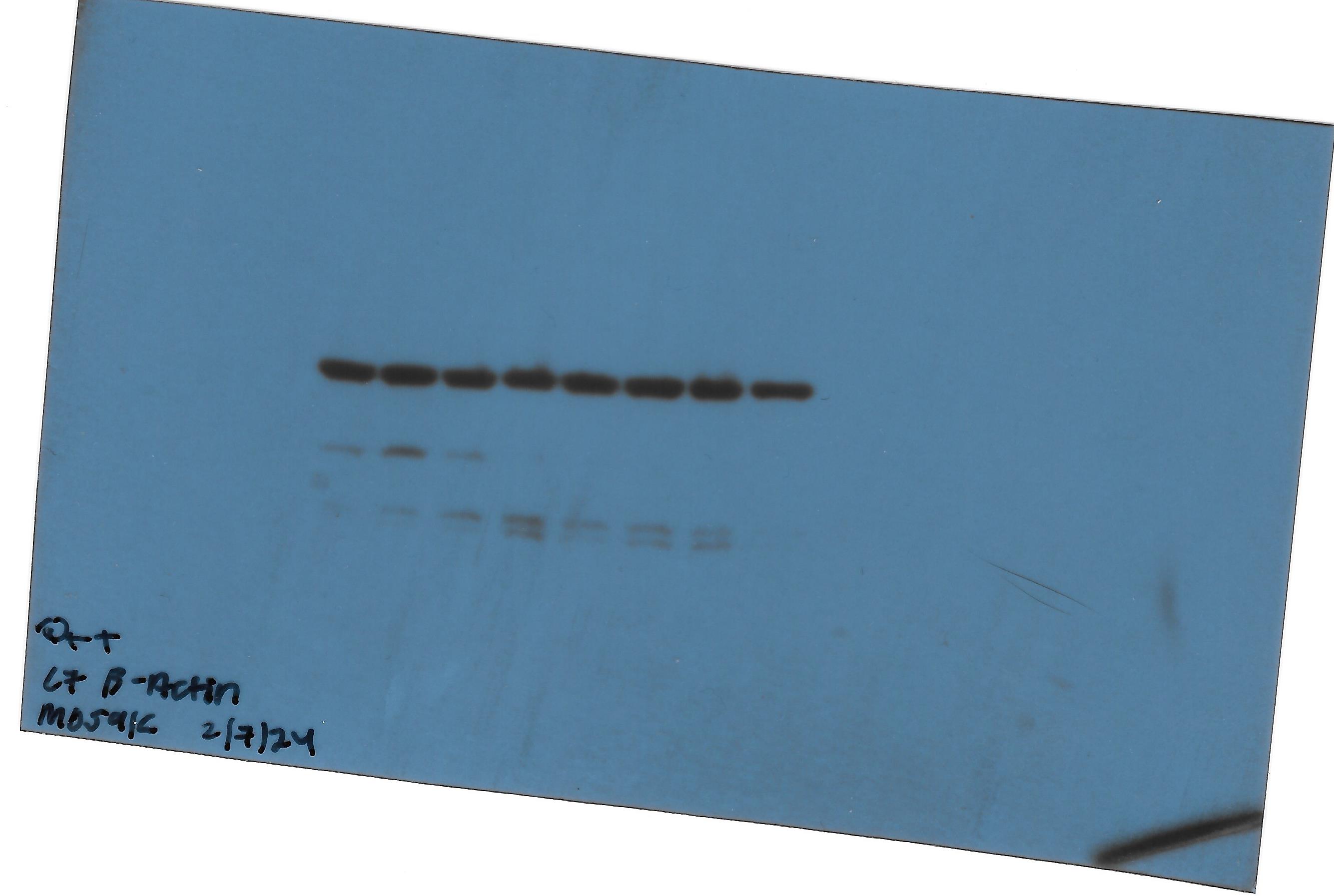

Supplement: Supplementary file 1 [file cancers-17-03197-s001.zip › OriginalBlots/Figure2A-M059K/2024-02-07_M059K_C7_Actin_2.jpg]

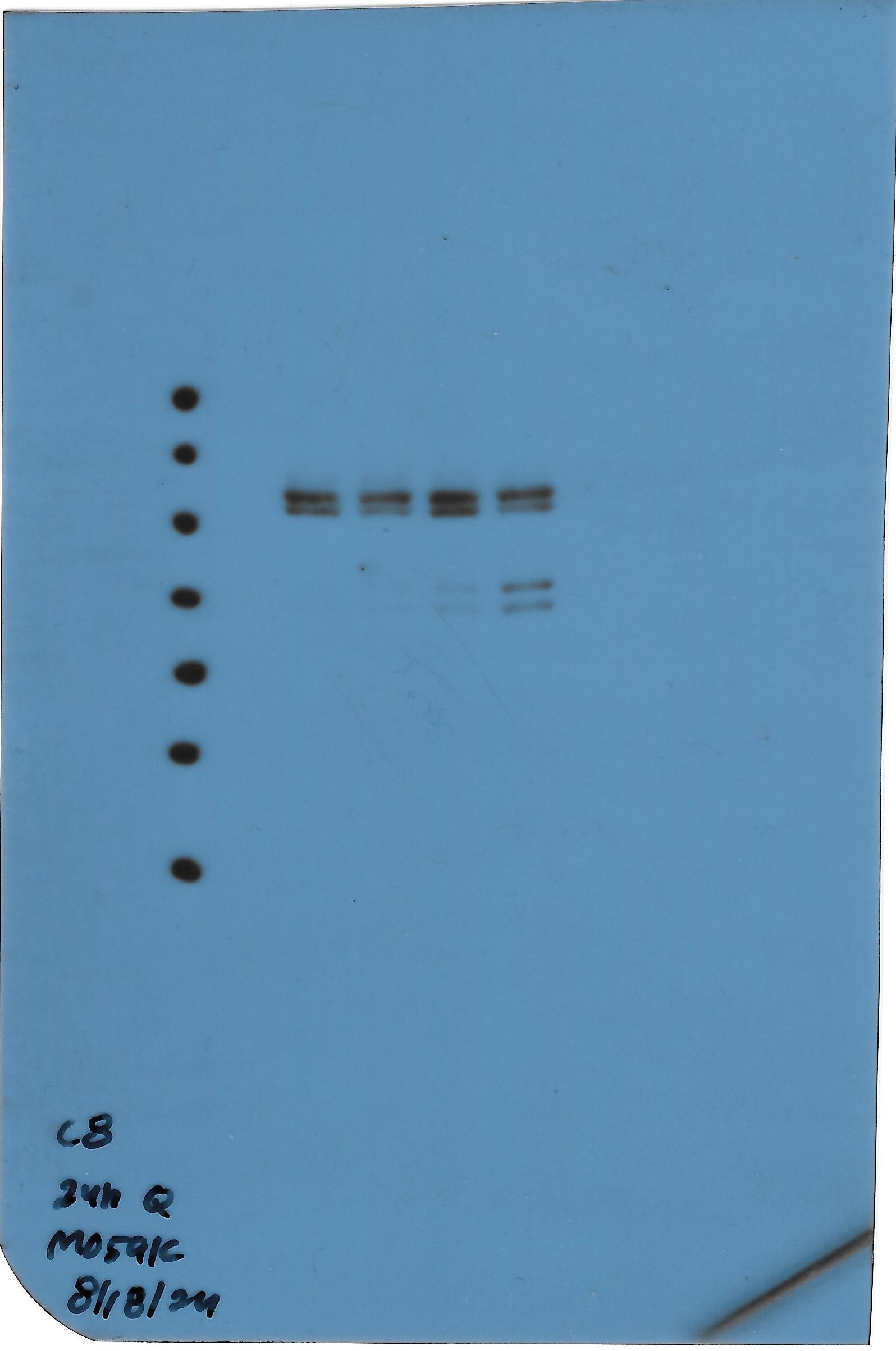

Supplement: Supplementary file 1 [file cancers-17-03197-s001.zip › OriginalBlots/Figure2A-M059K/2024-08-18_M059K_24h_Qonly_C8_1.jpg]

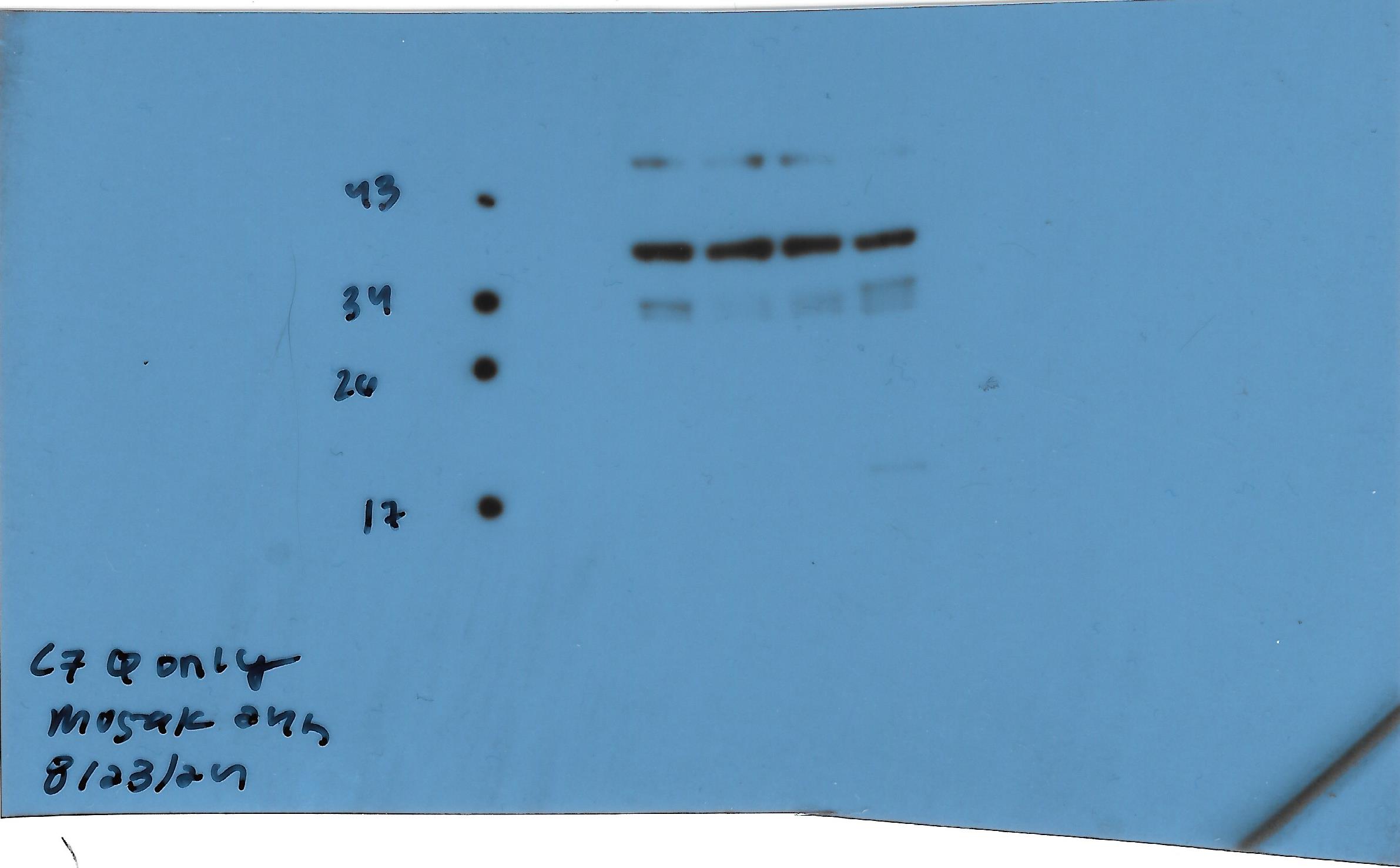

Supplement: Supplementary file 1 [file cancers-17-03197-s001.zip › OriginalBlots/Figure2A-M059K/2024-08-23_M059K_48h_Qonly_C7_2.jpg]

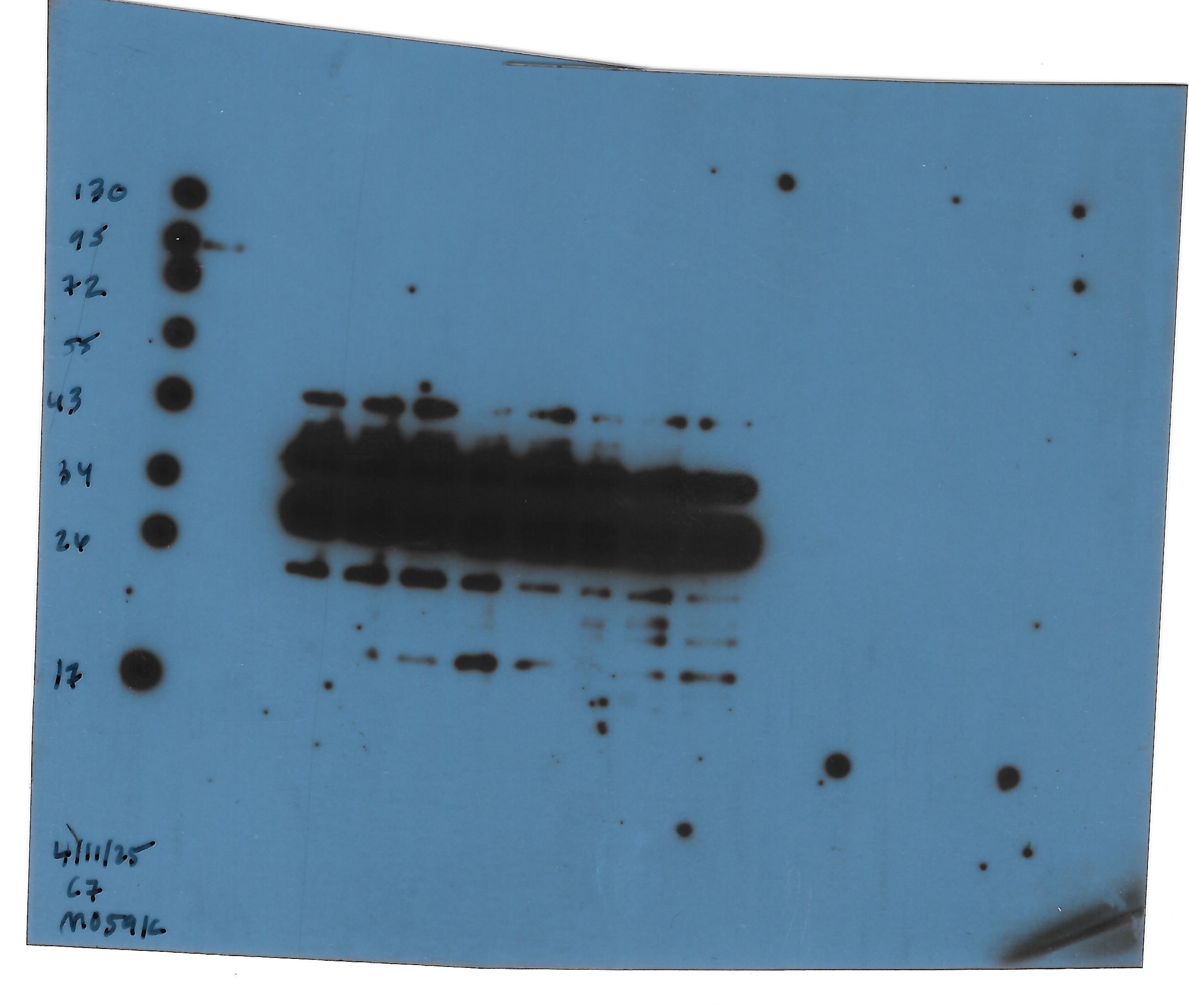

Supplement: Supplementary file 1 [file cancers-17-03197-s001.zip › OriginalBlots/Figure2A-M059K/2025-04-11_M059K_C7_1.jpg]

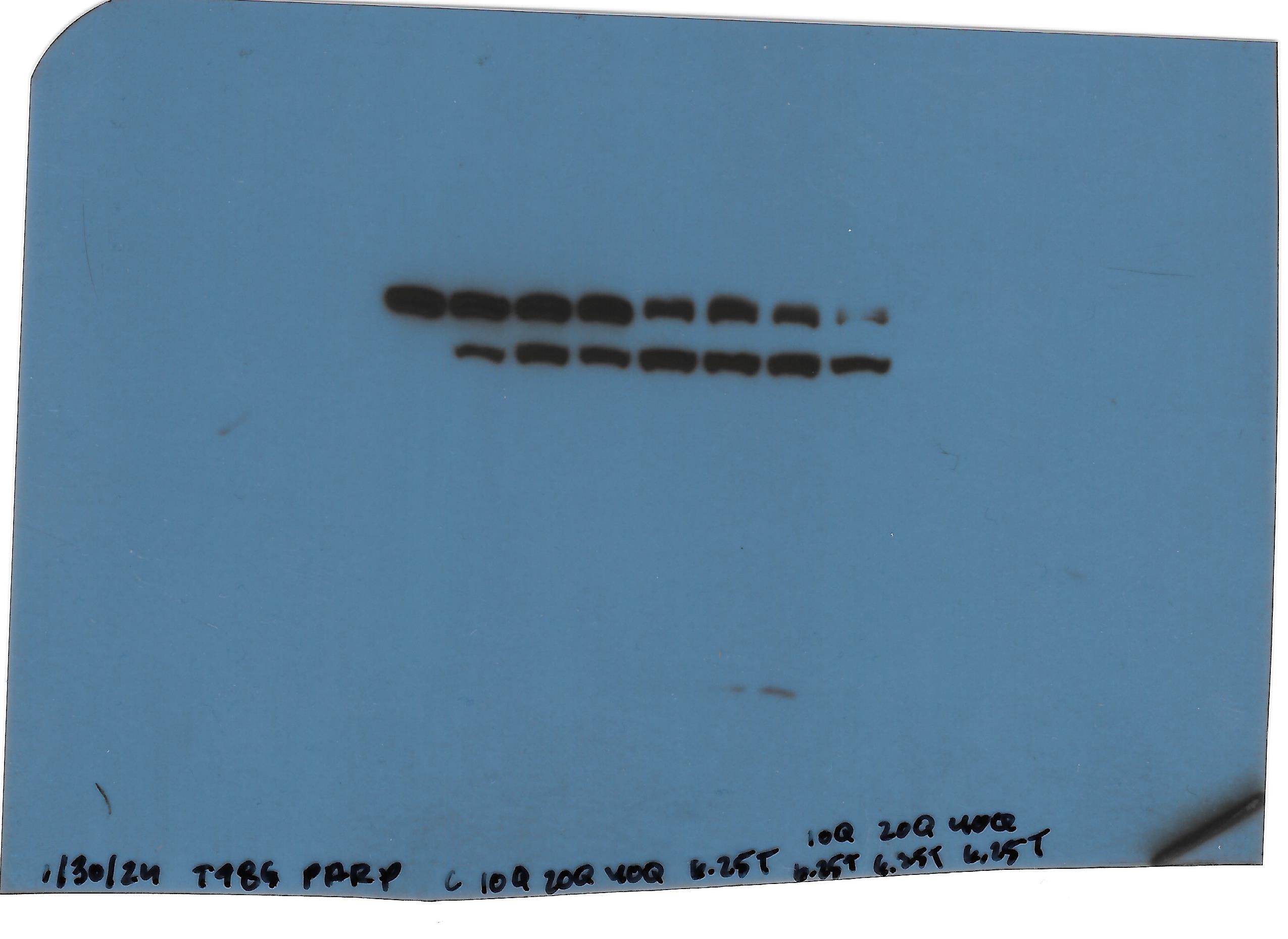

Supplement: Supplementary file 1 [file cancers-17-03197-s001.zip › OriginalBlots/Figure2B-T98G/2025-01-30_T98G_Q+T_PARP.jpg]

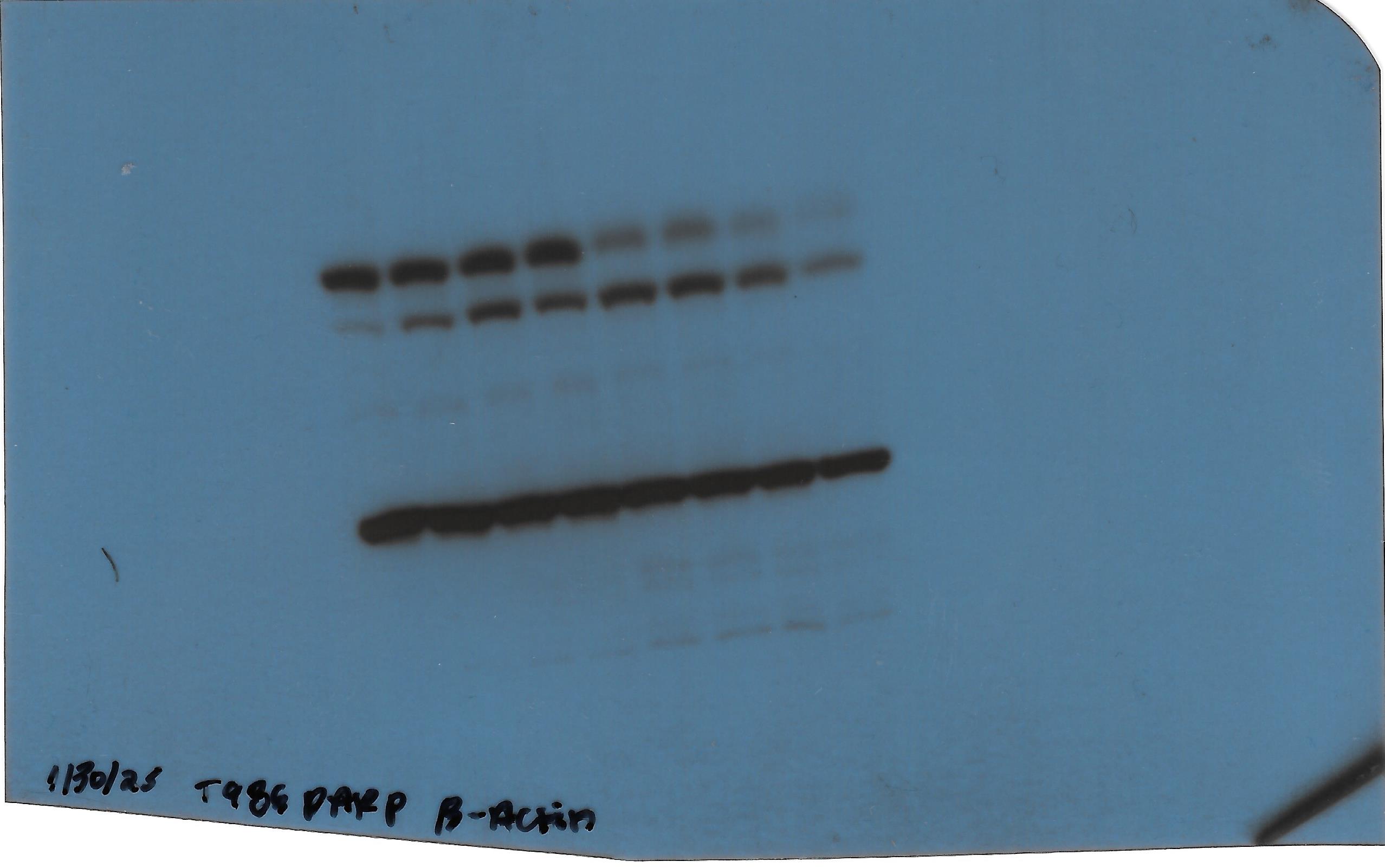

Supplement: Supplementary file 1 [file cancers-17-03197-s001.zip › OriginalBlots/Figure2B-T98G/2025-01-30_T98G_Q+T_PARP_Actin.jpg]

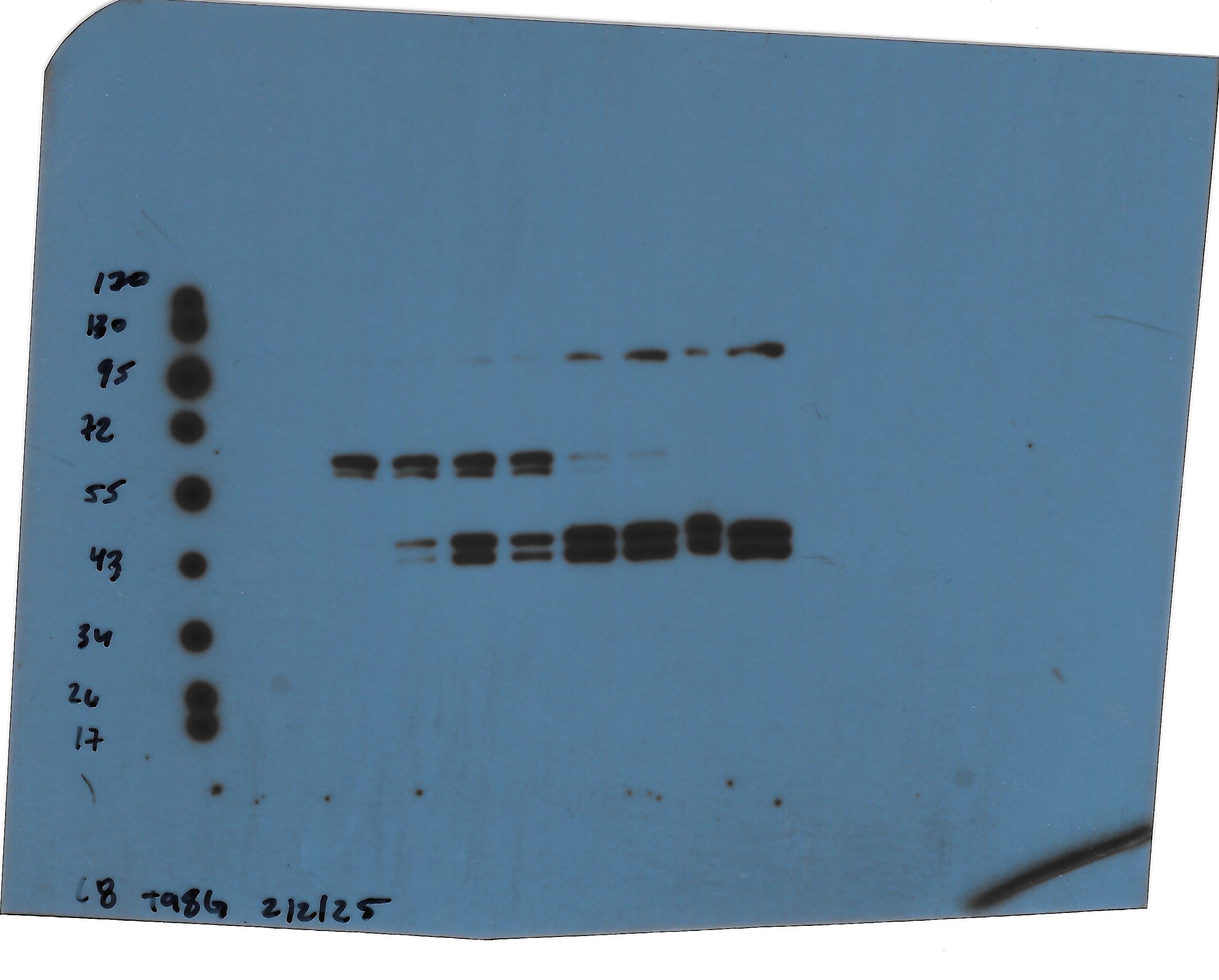

Supplement: Supplementary file 1 [file cancers-17-03197-s001.zip › OriginalBlots/Figure2B-T98G/2025-02-02_T98G_Q+T_C8_2.jpg]

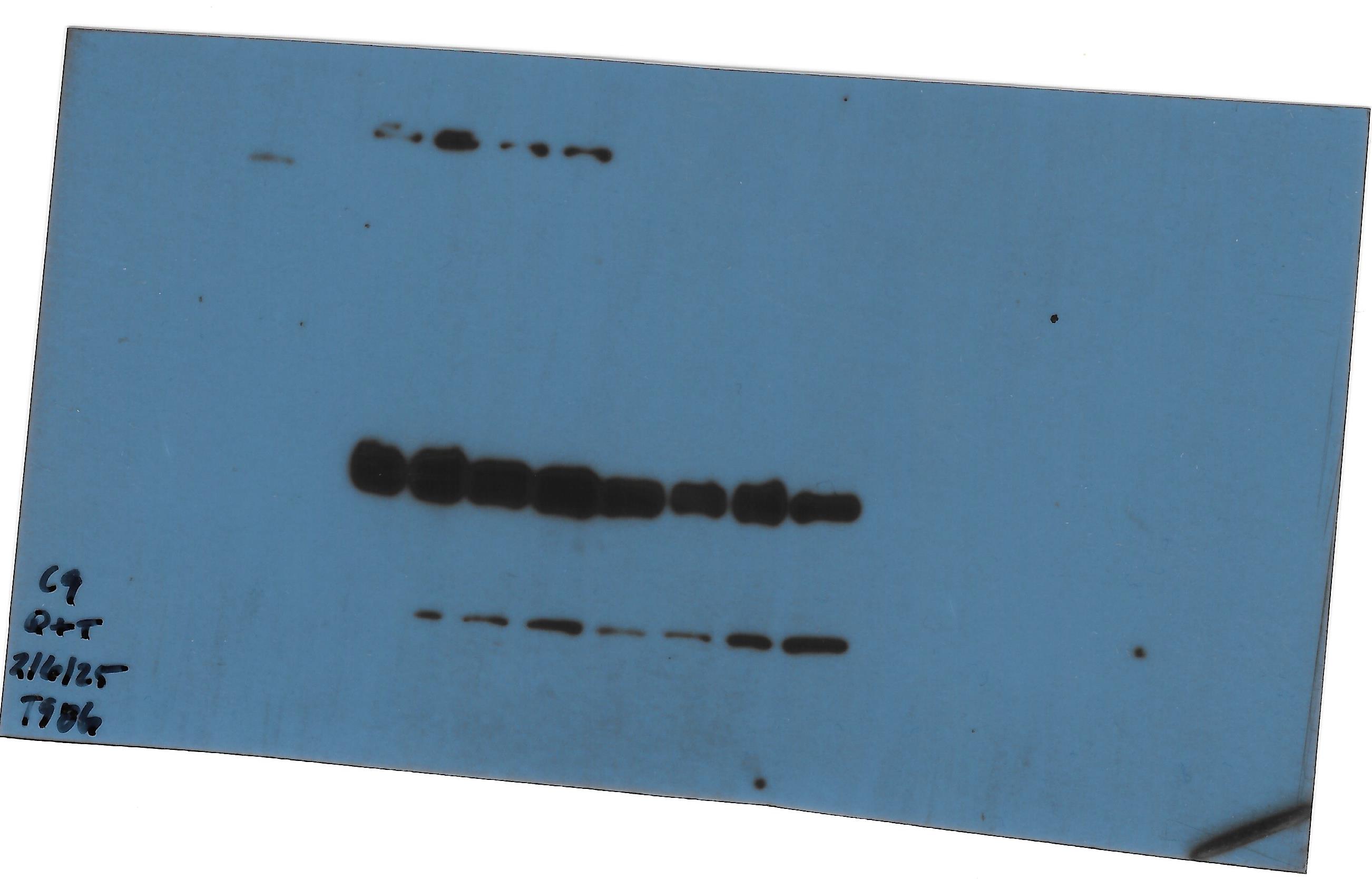

Supplement: Supplementary file 1 [file cancers-17-03197-s001.zip › OriginalBlots/Figure2B-T98G/2025-02-06_T98G_Q+T_C9_1.jpg]

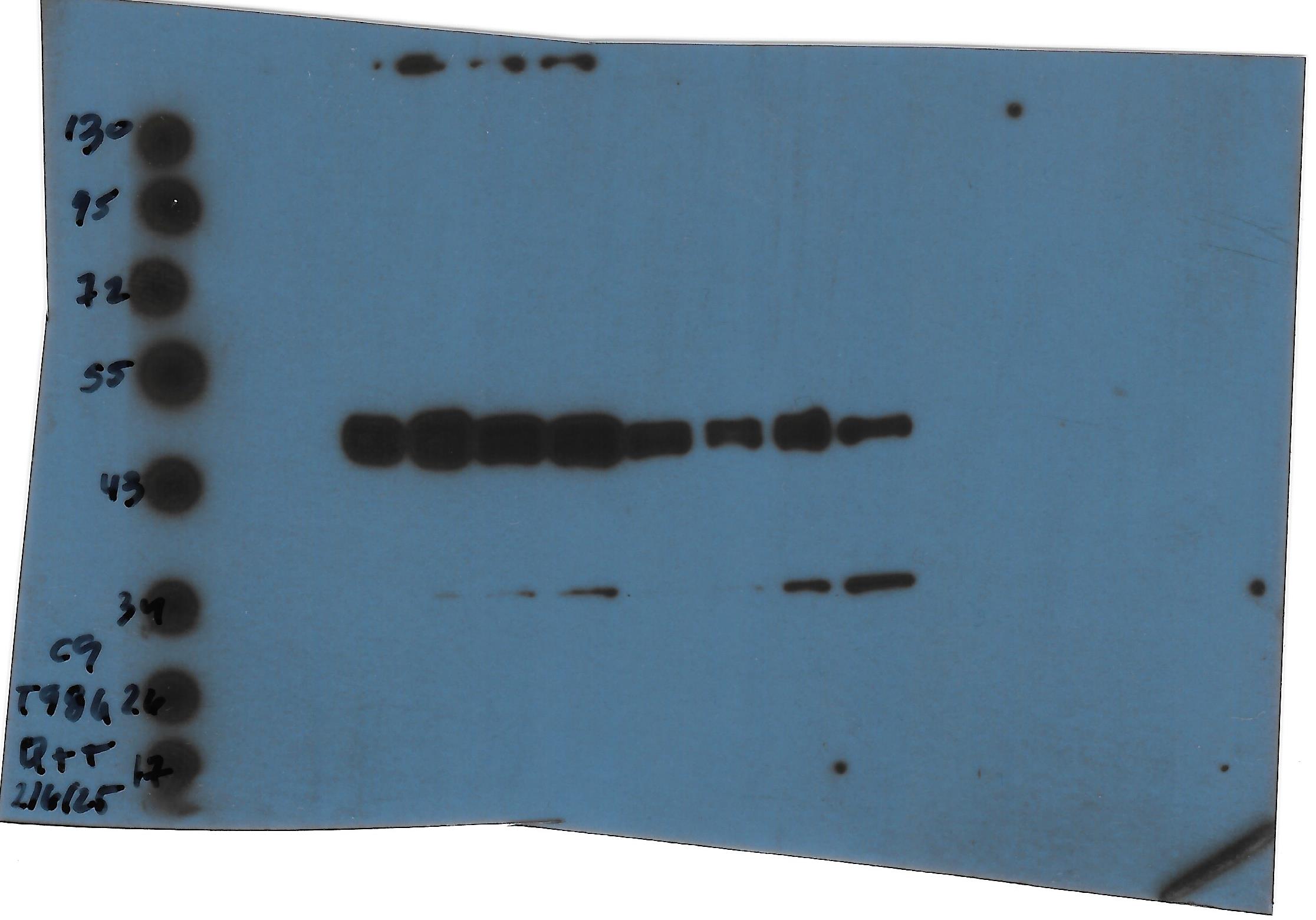

Supplement: Supplementary file 1 [file cancers-17-03197-s001.zip › OriginalBlots/Figure2B-T98G/2025-02-06_T98G_Q+T_C9_2.jpg]

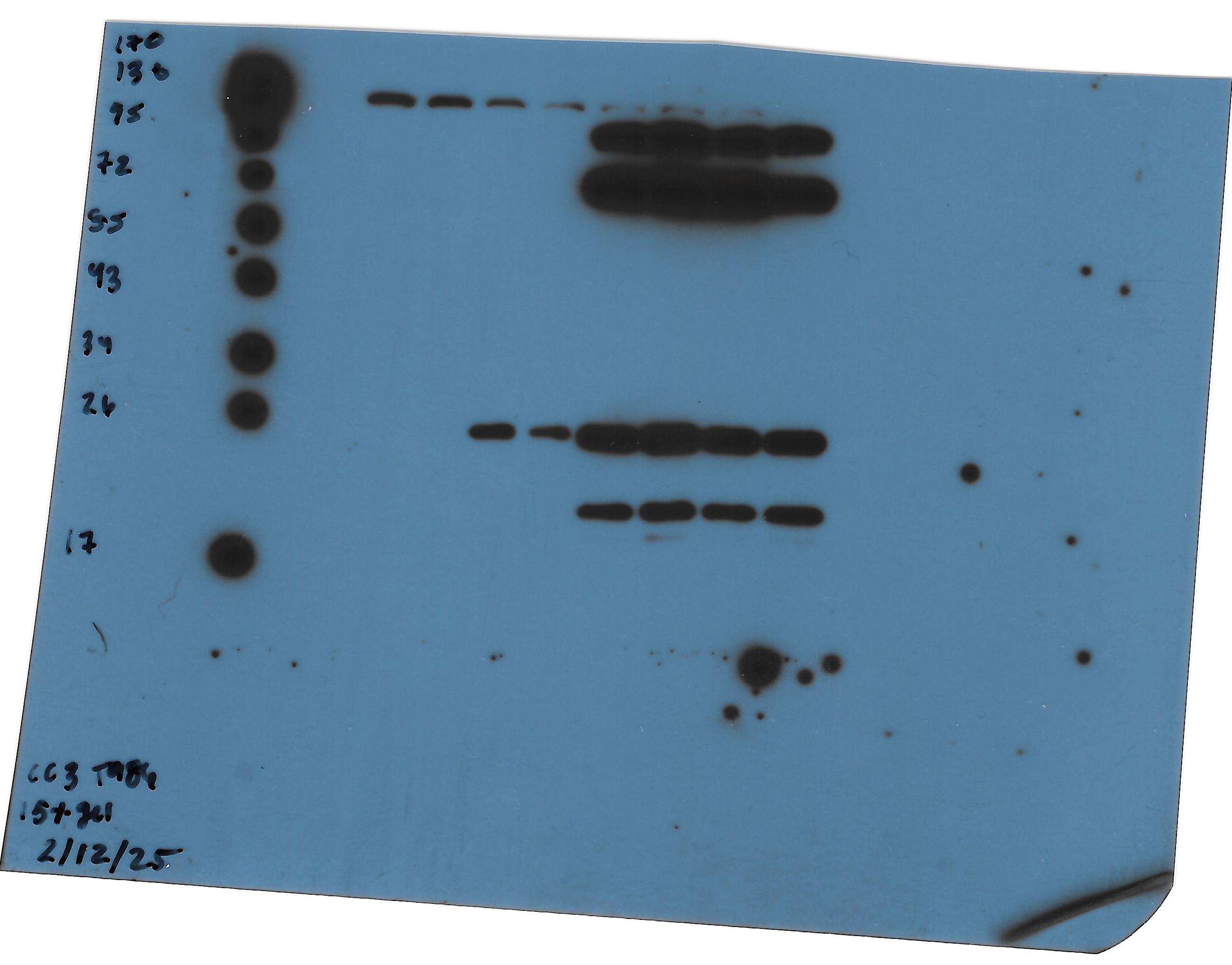

Supplement: Supplementary file 1 [file cancers-17-03197-s001.zip › OriginalBlots/Figure2B-T98G/2025-02-12_T98G_Q+T_CC3_1.jpg]

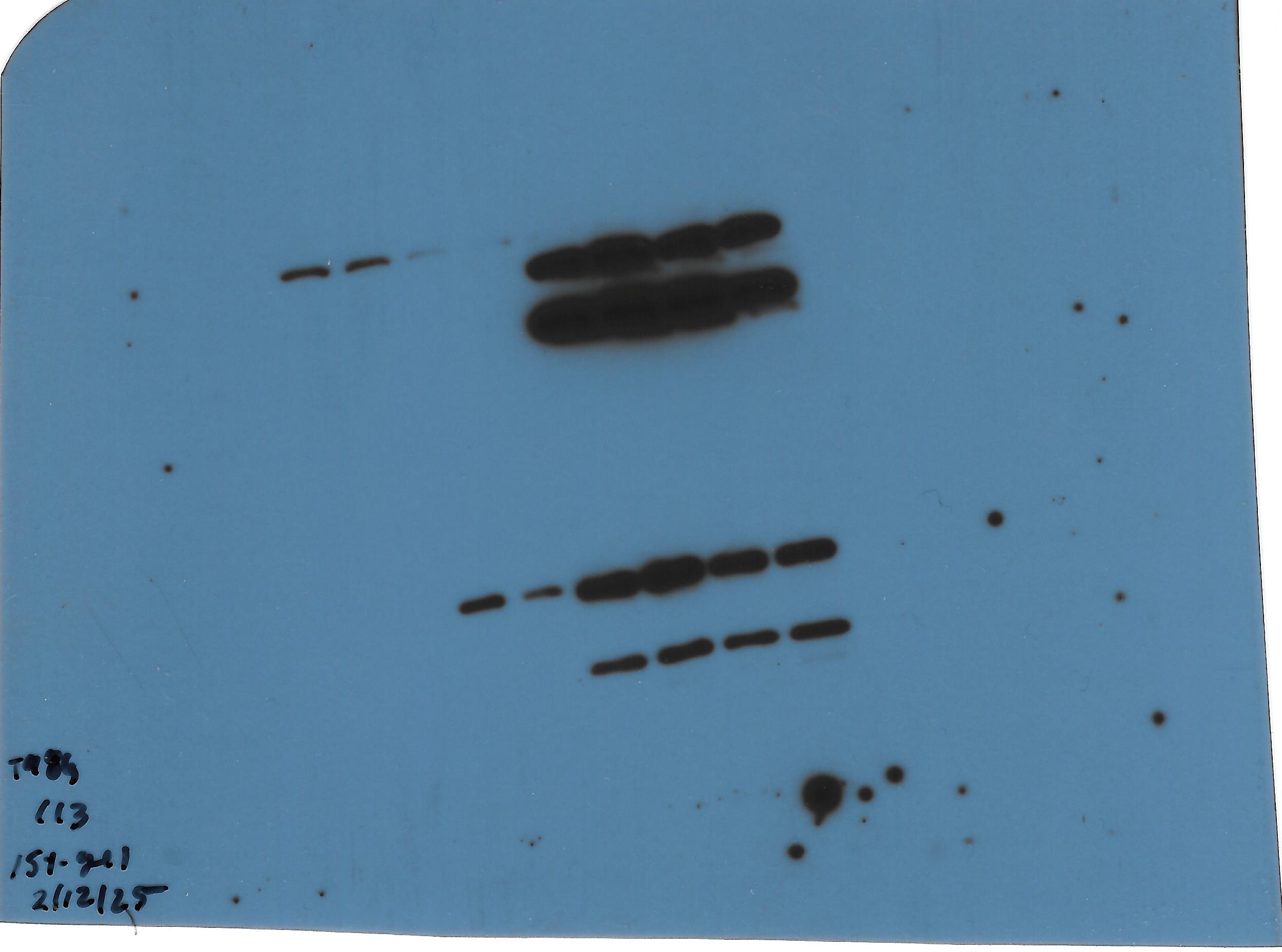

Supplement: Supplementary file 1 [file cancers-17-03197-s001.zip › OriginalBlots/Figure2B-T98G/2025-02-12_T98G_Q+T_CC3_2.jpg]

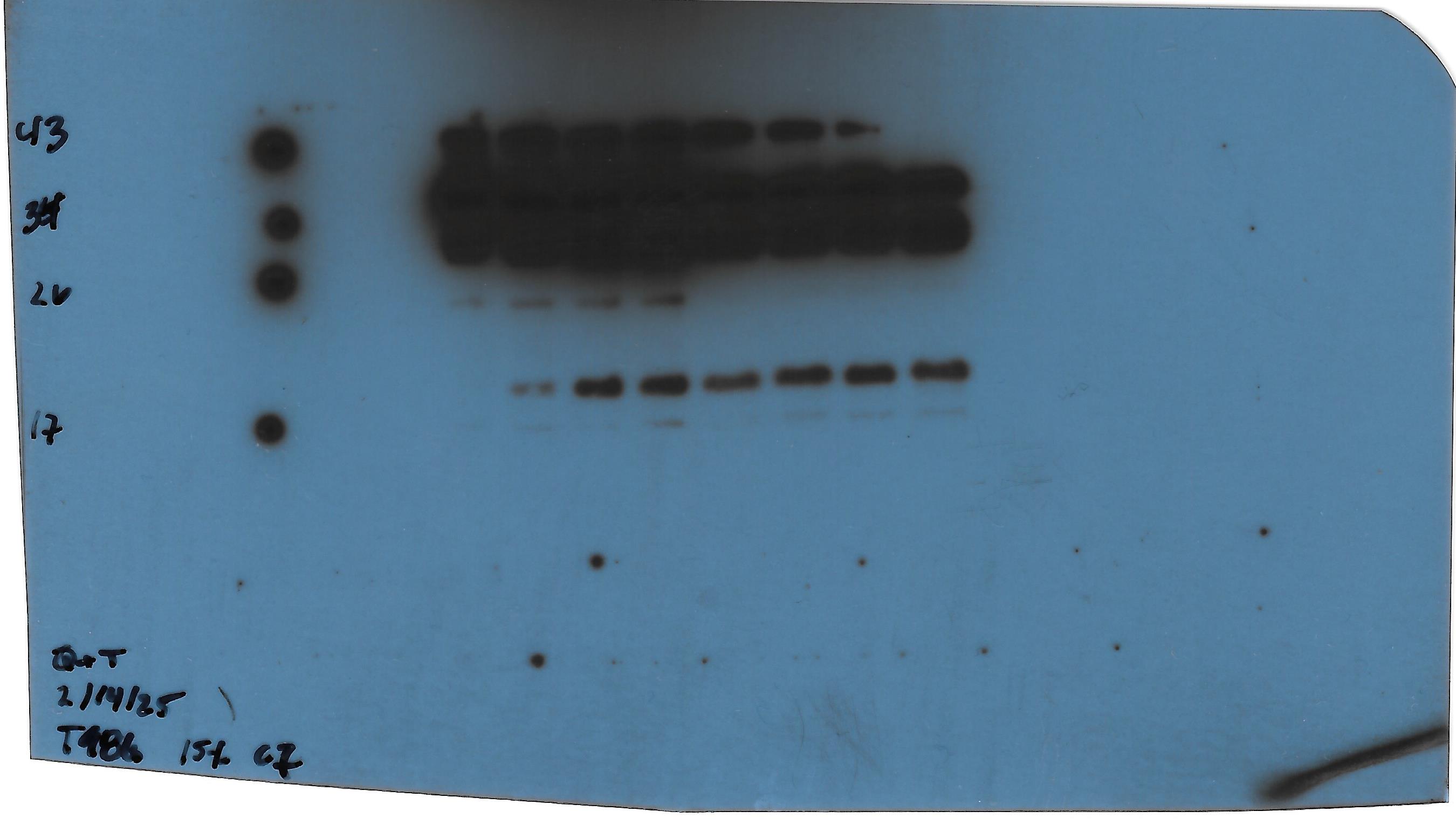

Supplement: Supplementary file 1 [file cancers-17-03197-s001.zip › OriginalBlots/Figure2B-T98G/2025-02-14_T98G_Q+T_C7_1.jpg]

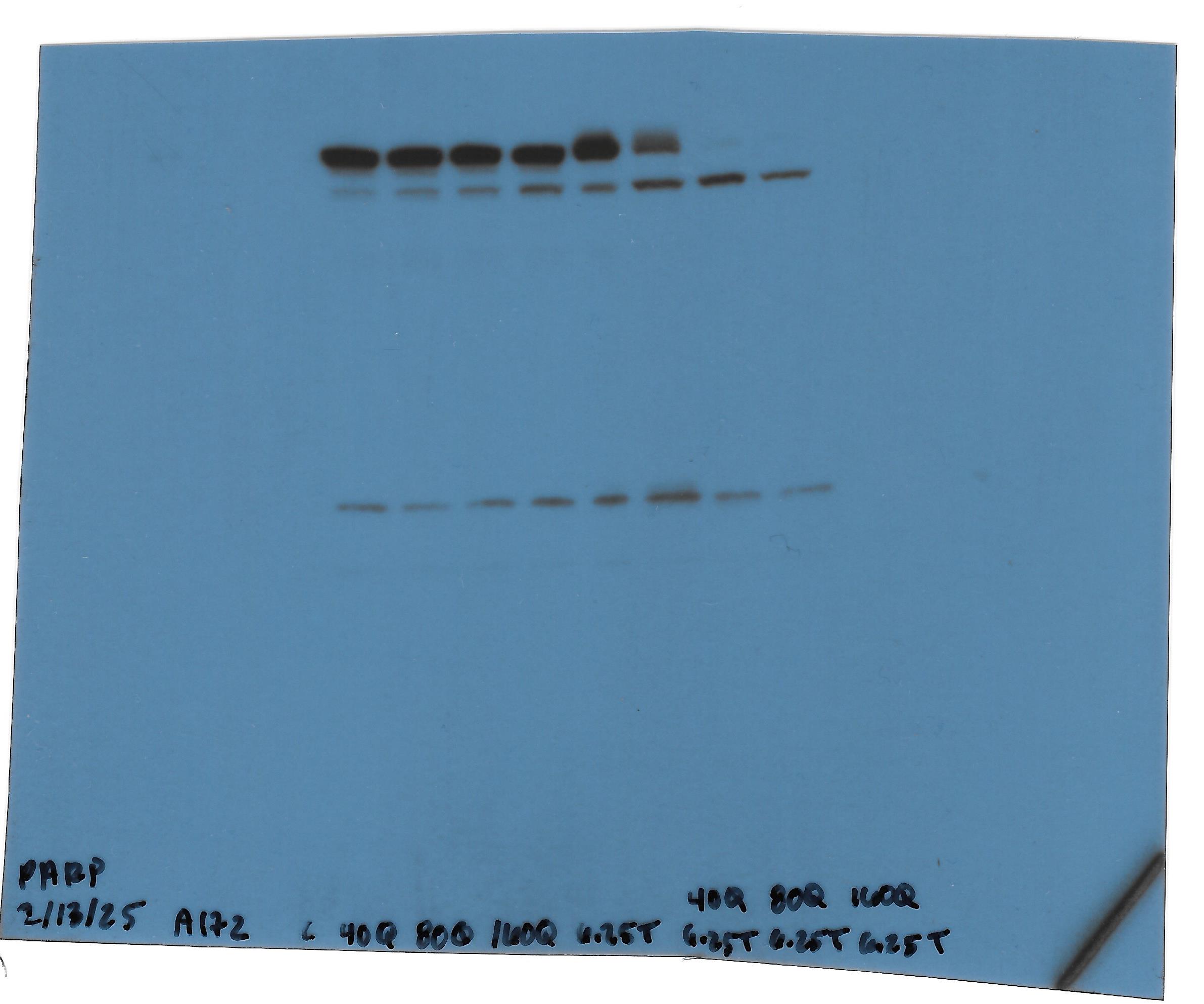

Supplement: Supplementary file 1 [file cancers-17-03197-s001.zip › OriginalBlots/Figure2C-A172/2025-02-13_A172_Q+T_PARP_2.jpg]

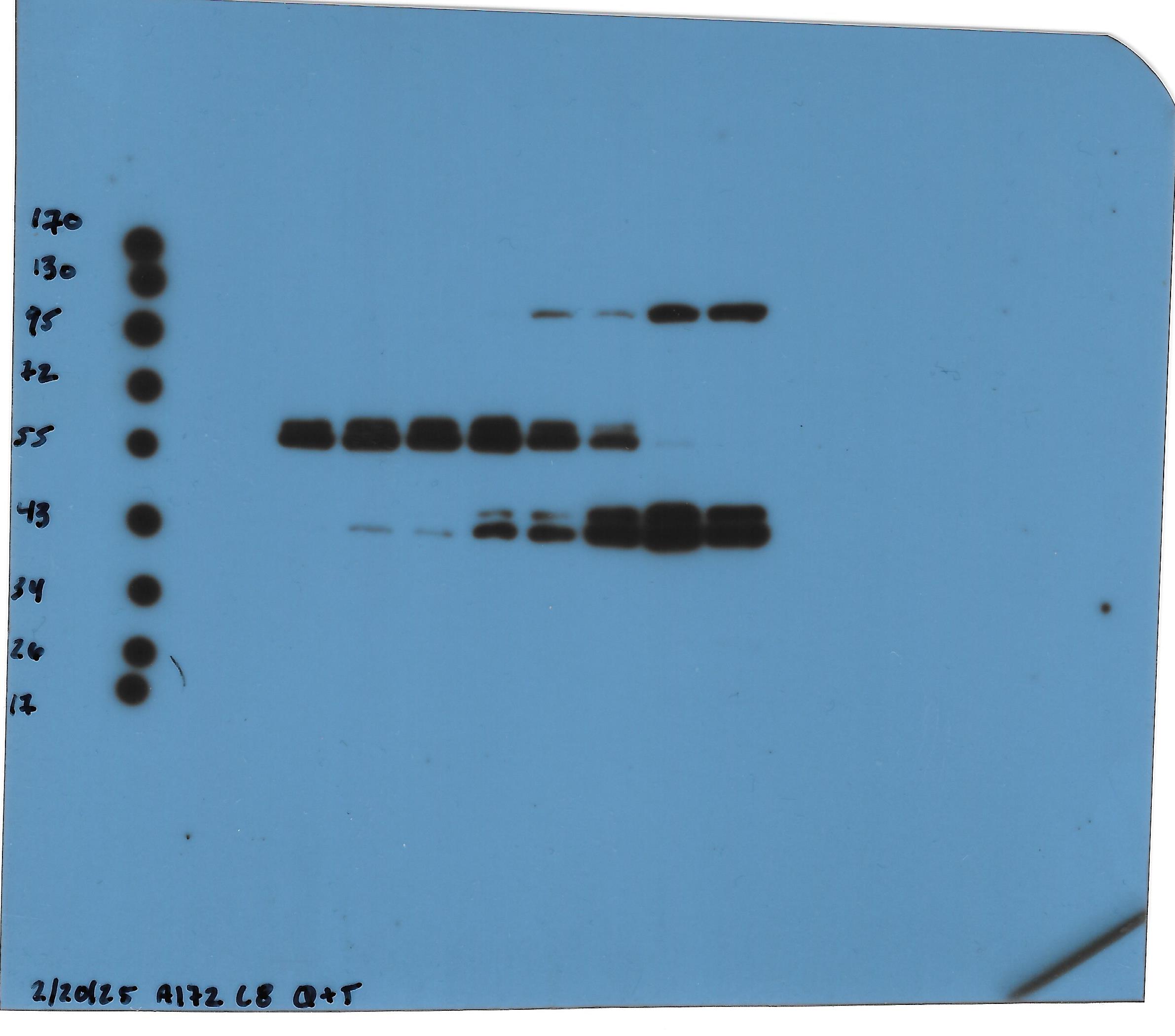

Supplement: Supplementary file 1 [file cancers-17-03197-s001.zip › OriginalBlots/Figure2C-A172/2025-02-20_A172_Q+T_C8_1.jpg]

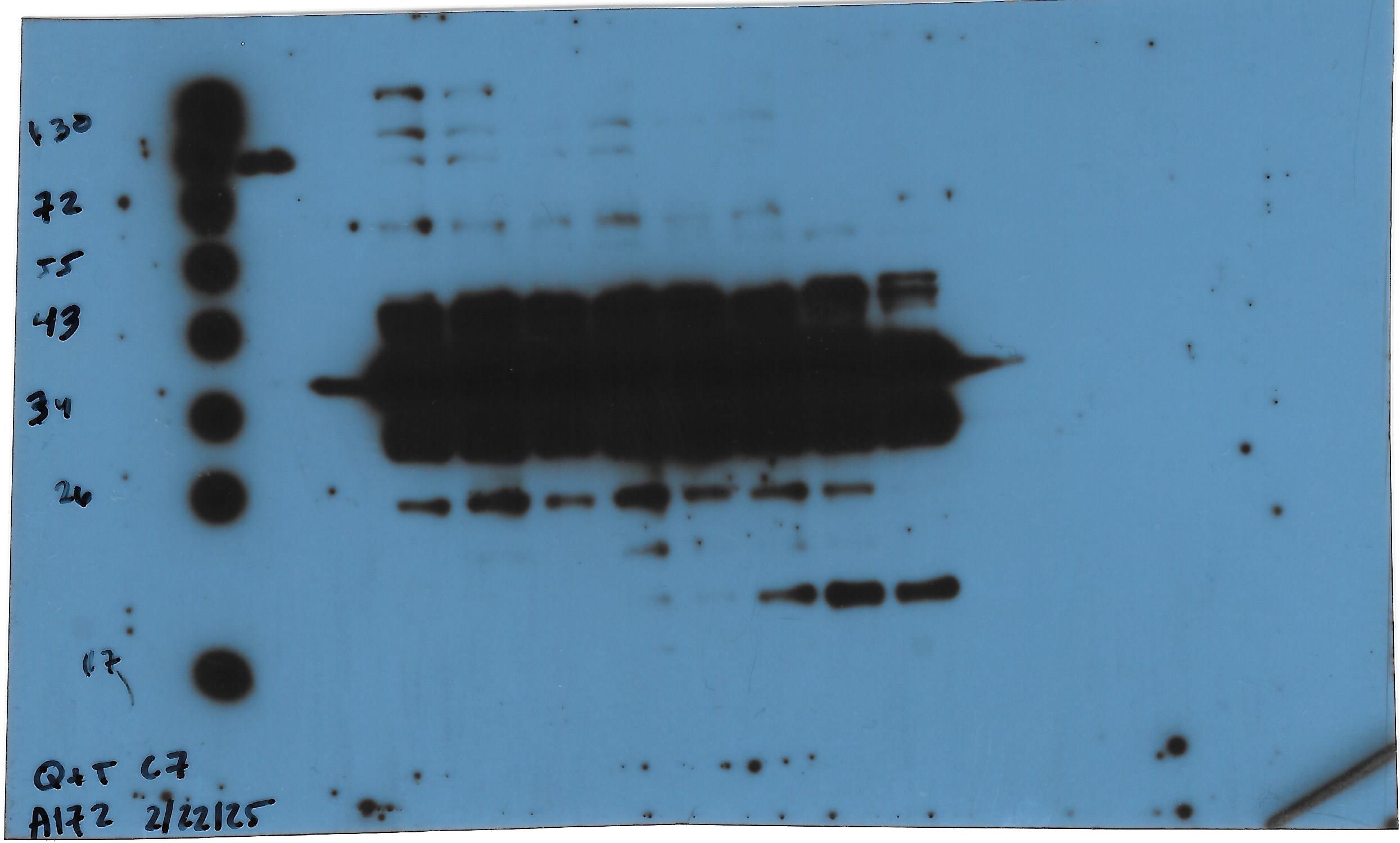

Supplement: Supplementary file 1 [file cancers-17-03197-s001.zip › OriginalBlots/Figure2C-A172/2025-02-22_A172_Q+T_C7_1.jpg]

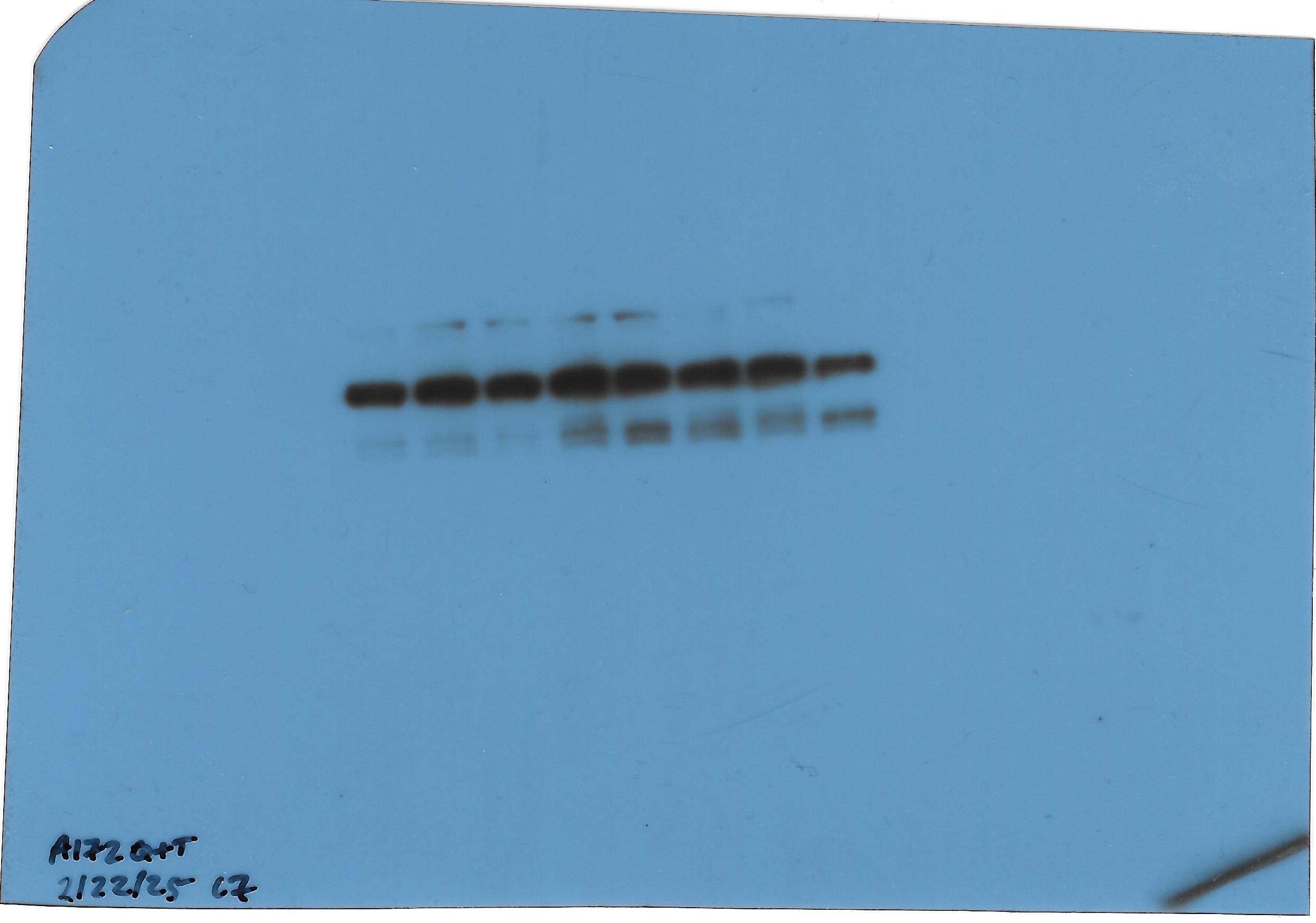

Supplement: Supplementary file 1 [file cancers-17-03197-s001.zip › OriginalBlots/Figure2C-A172/2025-02-22_A172_Q+T_C7_6.jpg]

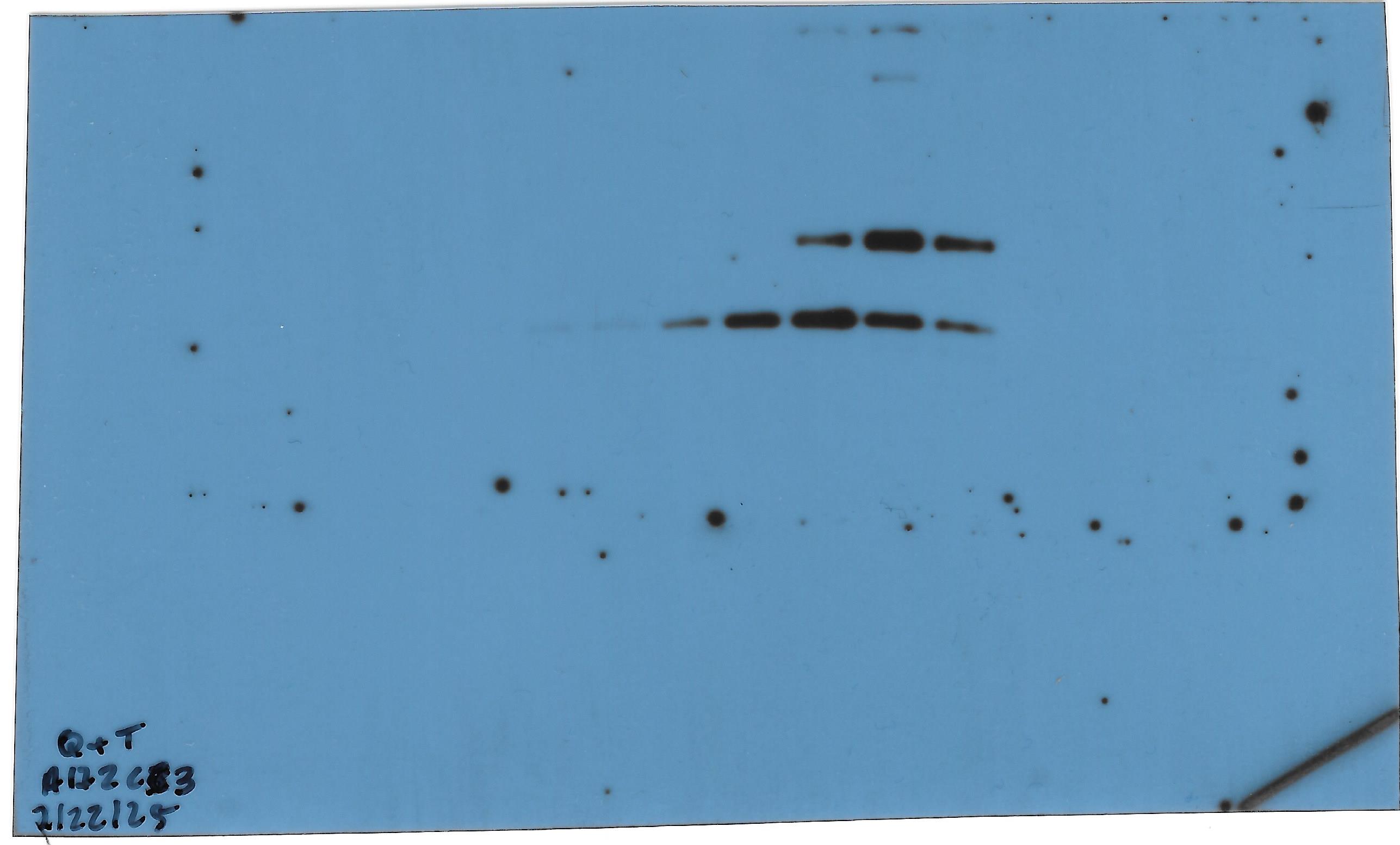

Supplement: Supplementary file 1 [file cancers-17-03197-s001.zip › OriginalBlots/Figure2C-A172/2025-02-22_A172_Q+T_CC3_2.jpg]

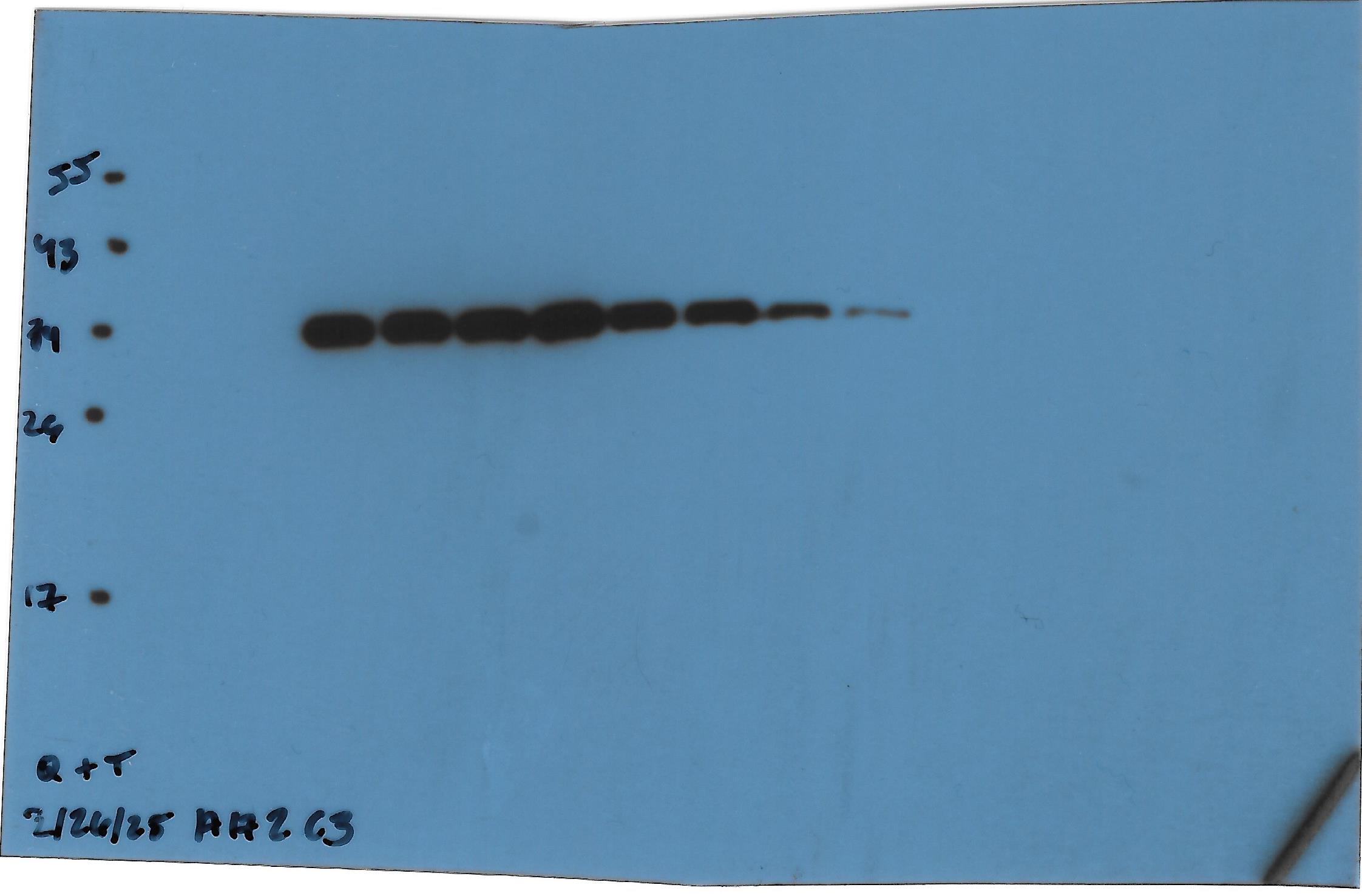

Supplement: Supplementary file 1 [file cancers-17-03197-s001.zip › OriginalBlots/Figure2C-A172/2025-02-26_A172_Q+T_C3_2.jpg]

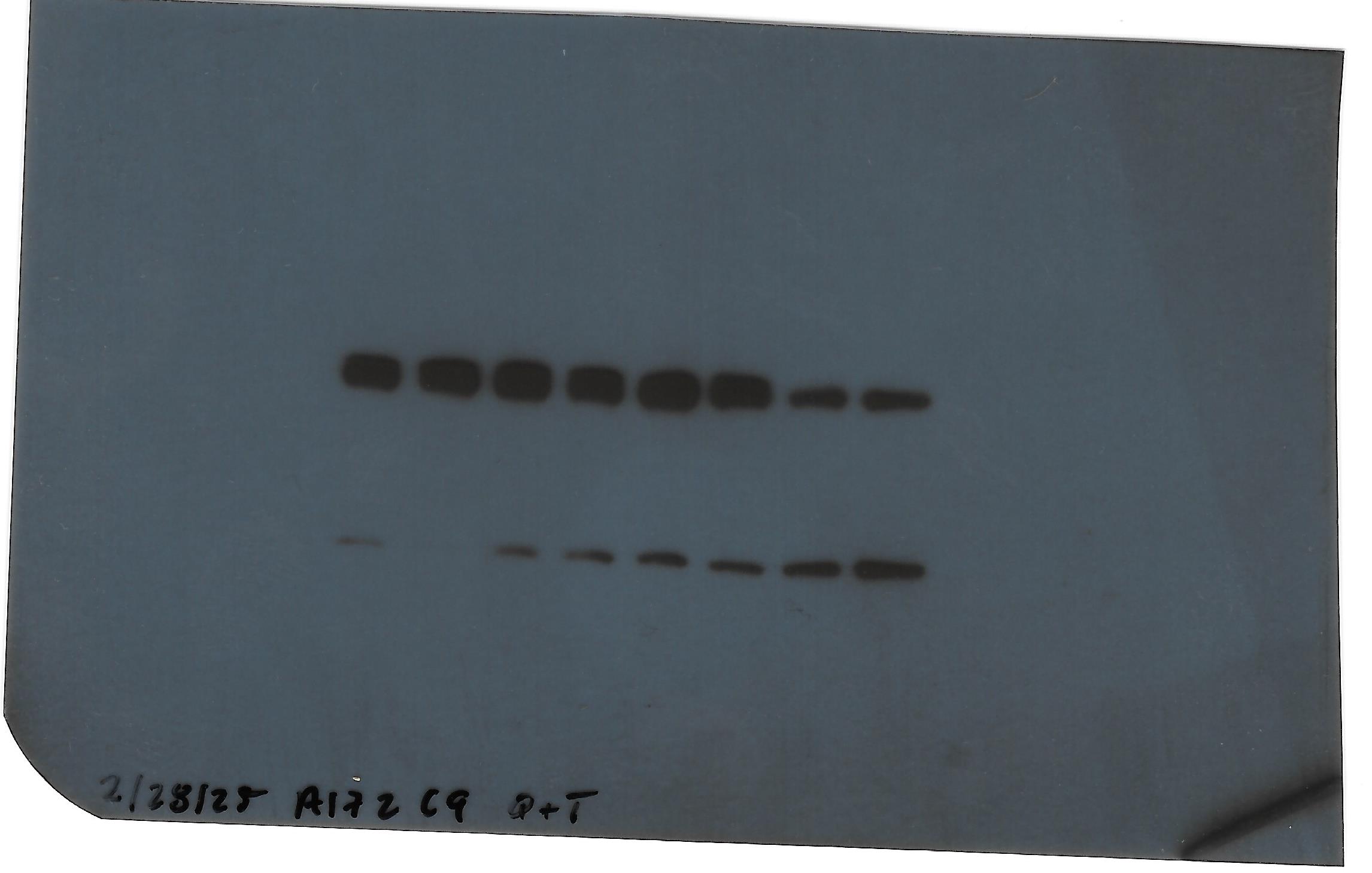

Supplement: Supplementary file 1 [file cancers-17-03197-s001.zip › OriginalBlots/Figure2C-A172/2025-02-28_A172_Q+T_C9_4.jpg]

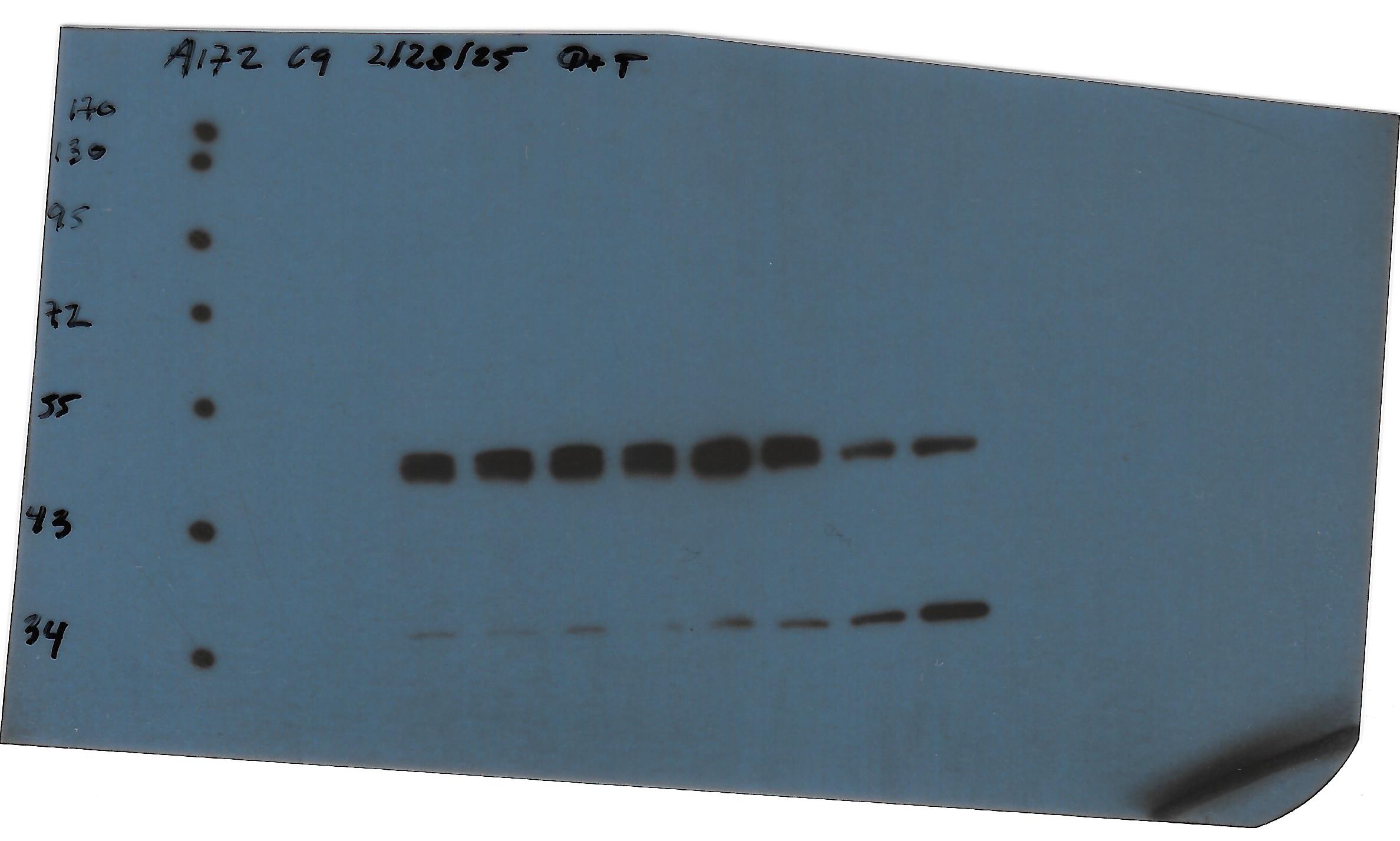

Supplement: Supplementary file 1 [file cancers-17-03197-s001.zip › OriginalBlots/Figure2C-A172/2025-02-28_A172_Q+T_C9_6.jpg]

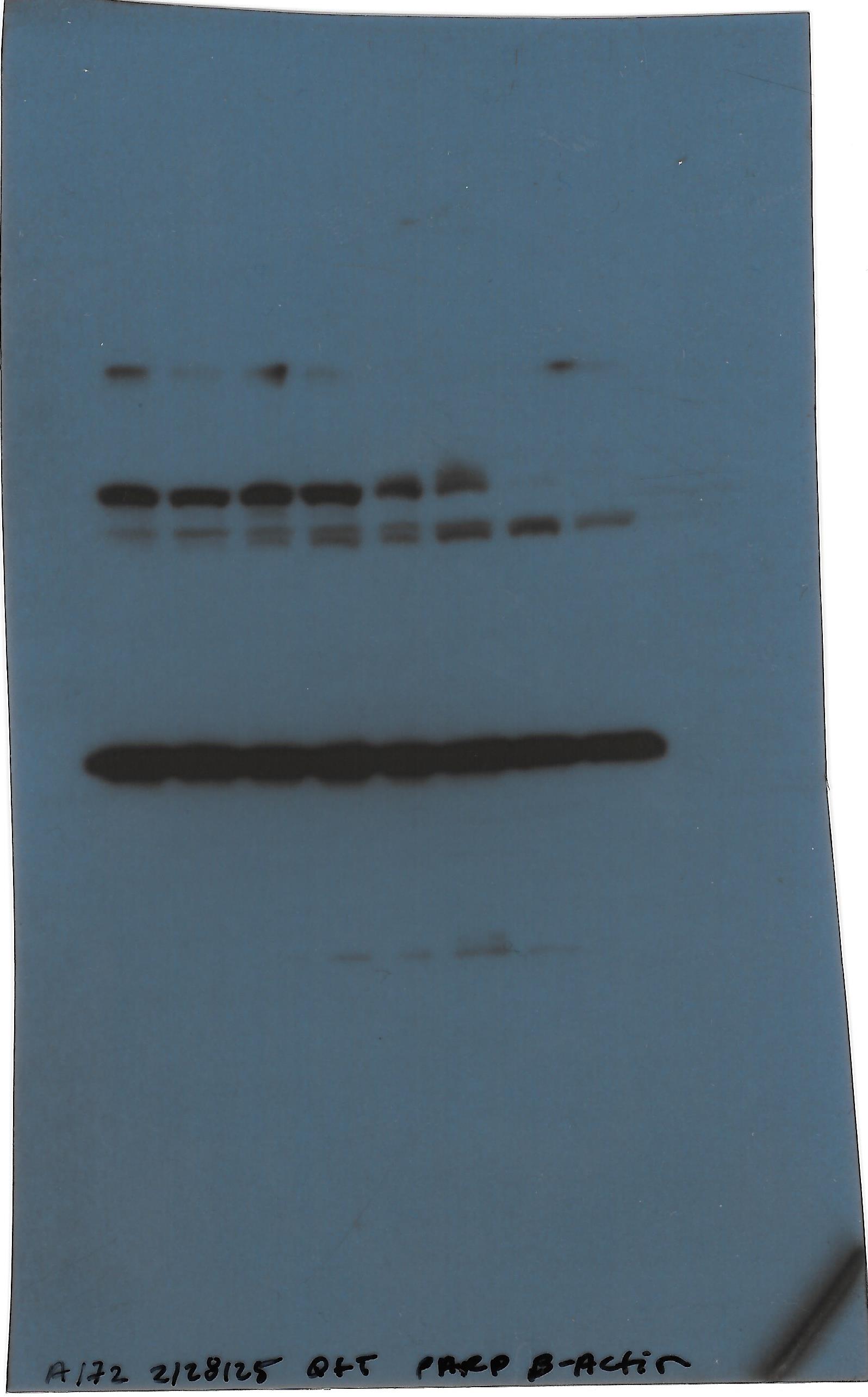

Supplement: Supplementary file 1 [file cancers-17-03197-s001.zip › OriginalBlots/Figure2C-A172/2025-02-28_A172_Q+T_PARP_Actin3.jpg]

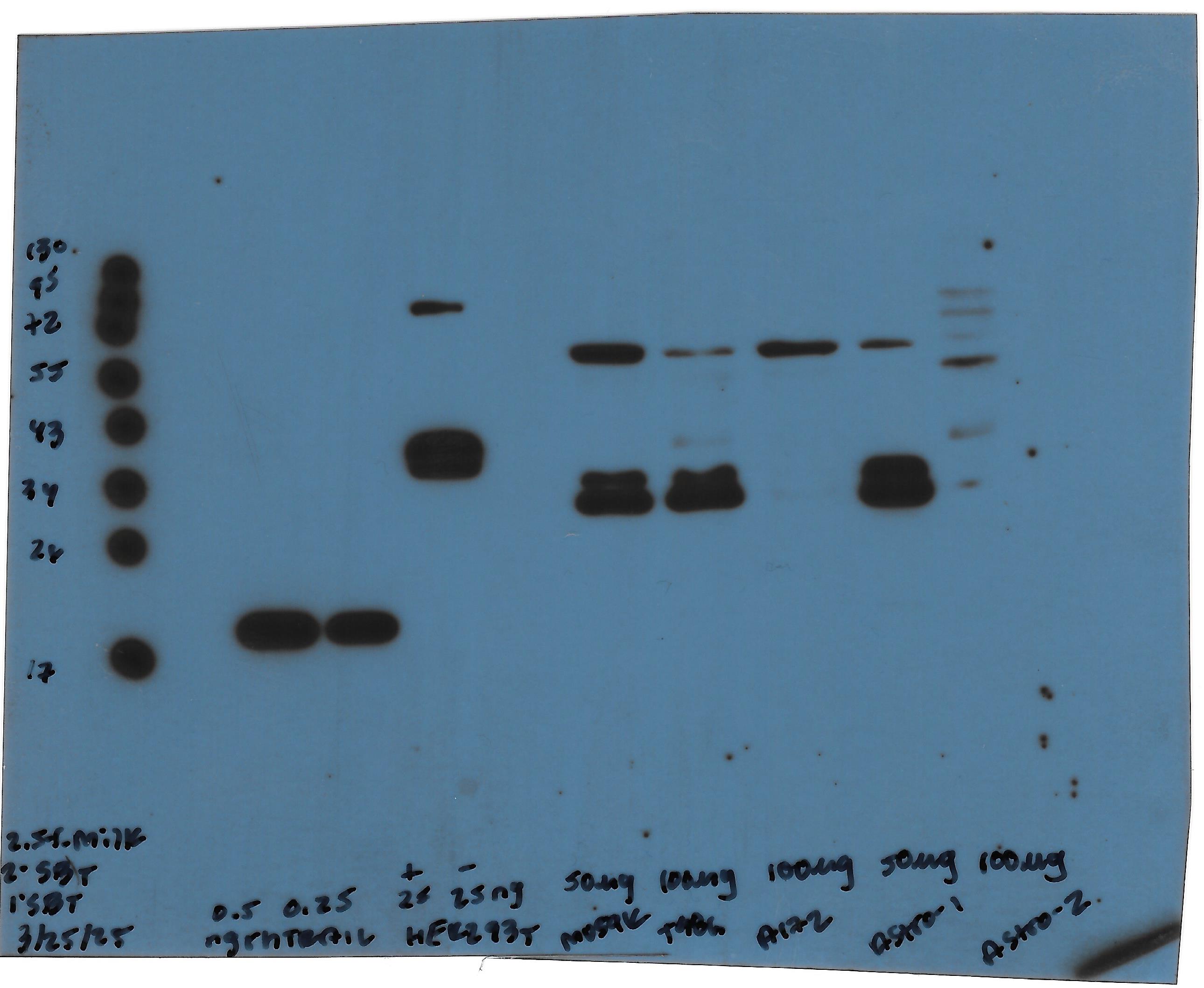

Supplement: Supplementary file 1 [file cancers-17-03197-s001.zip › OriginalBlots/Figure4/2025-03-25_SBT_2.5milk_1-333_15gel_4.jpg]

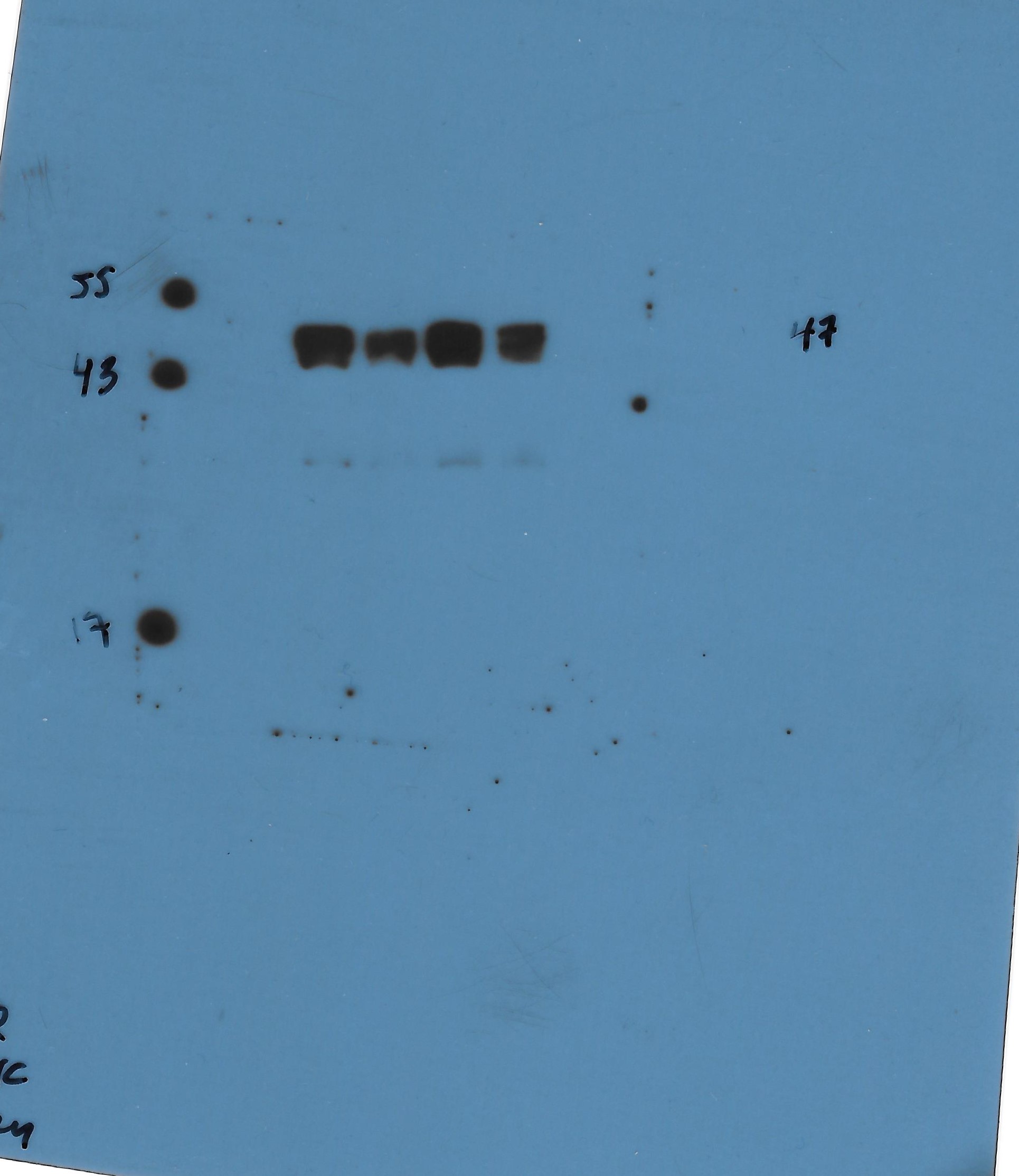

Supplement: Supplementary file 1 [file cancers-17-03197-s001.zip › OriginalBlots/FigureS1A-M059K-24h/2024-08-15_M059K_24h_Qonly_C9_1.jpg]

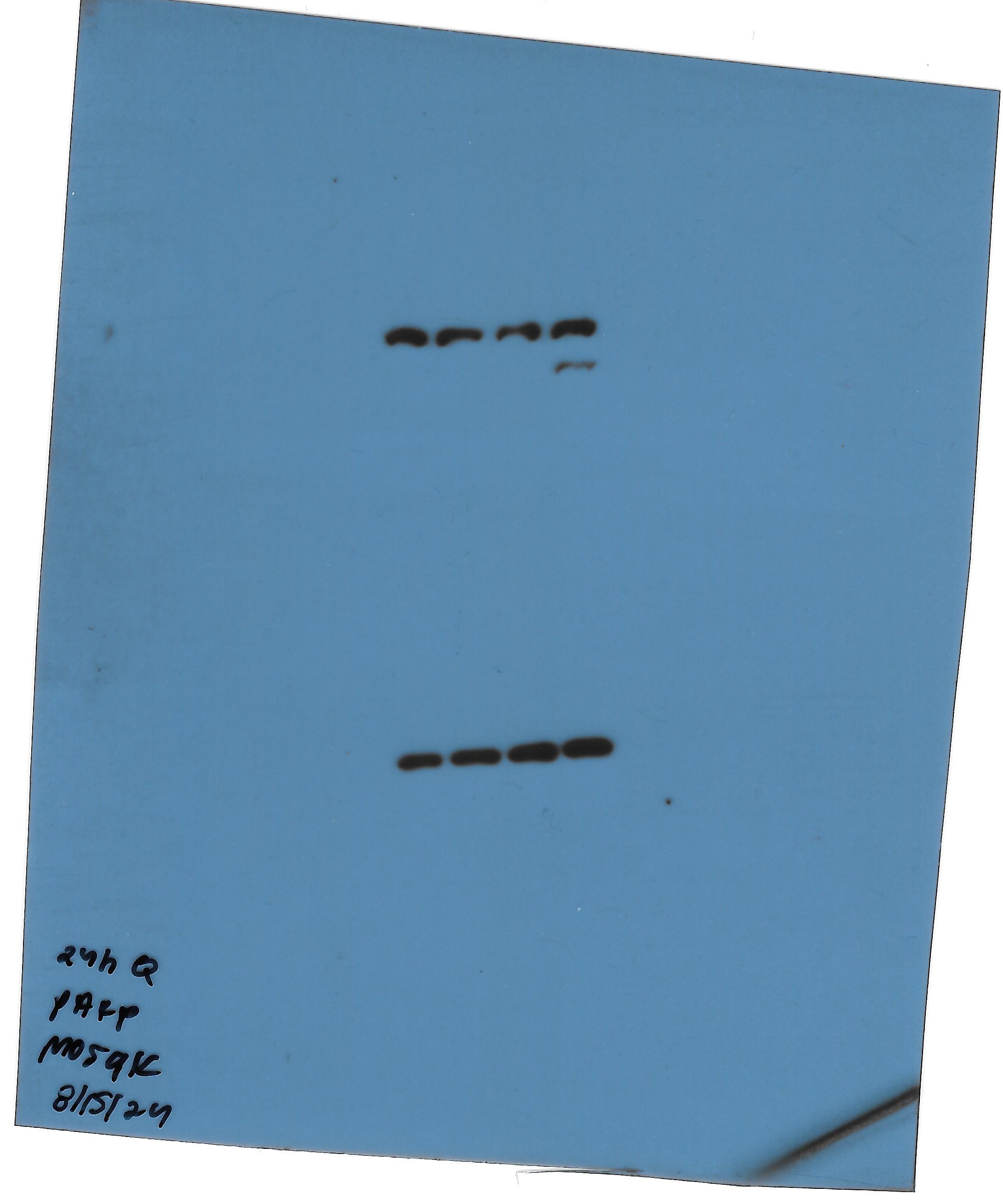

Supplement: Supplementary file 1 [file cancers-17-03197-s001.zip › OriginalBlots/FigureS1A-M059K-24h/2024-08-15_M059K_24h_Qonly_PARP_4.jpg]

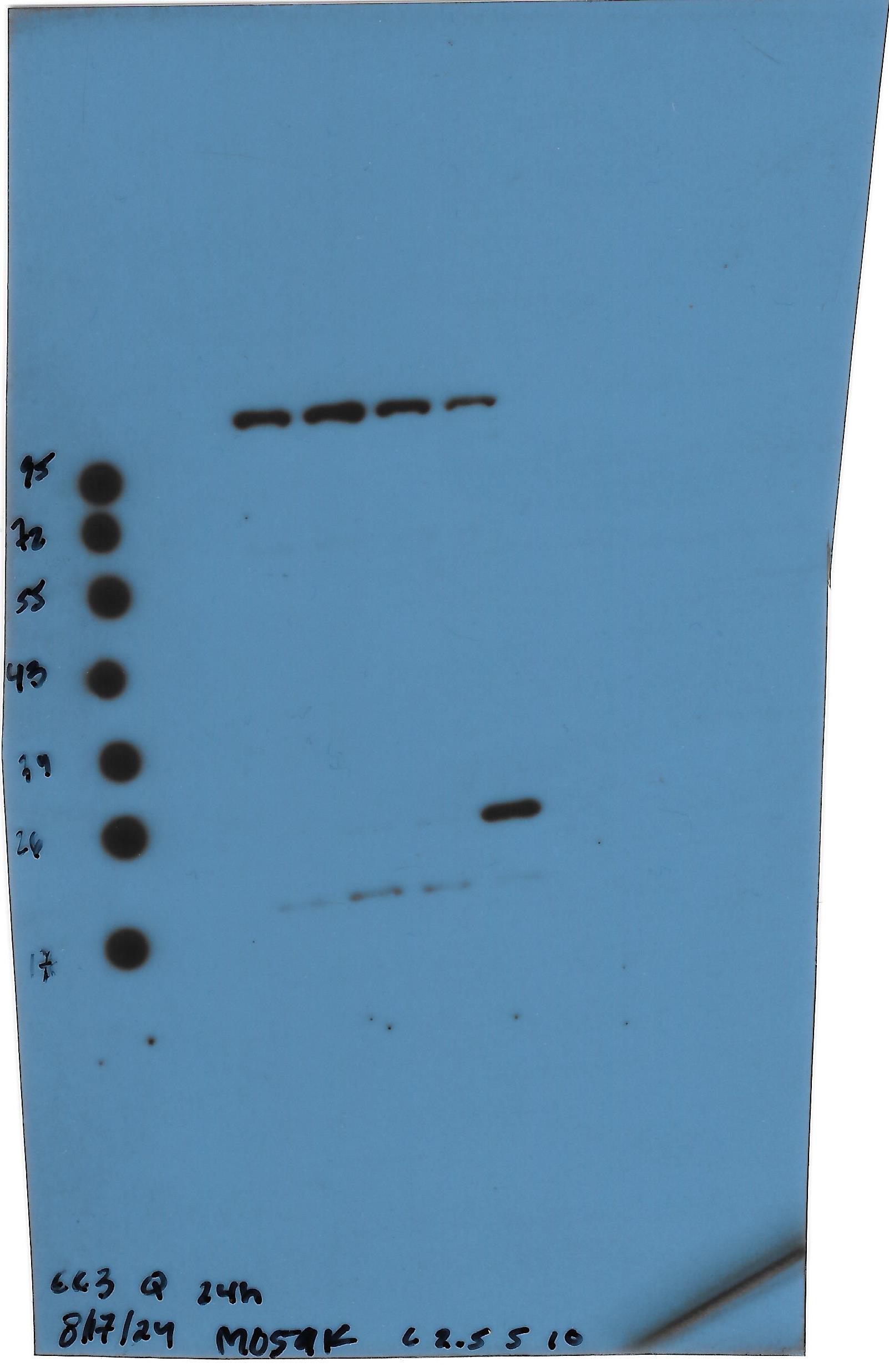

Supplement: Supplementary file 1 [file cancers-17-03197-s001.zip › OriginalBlots/FigureS1A-M059K-24h/2024-08-17_M059K_24h_Qonly_CC3_3.jpg]

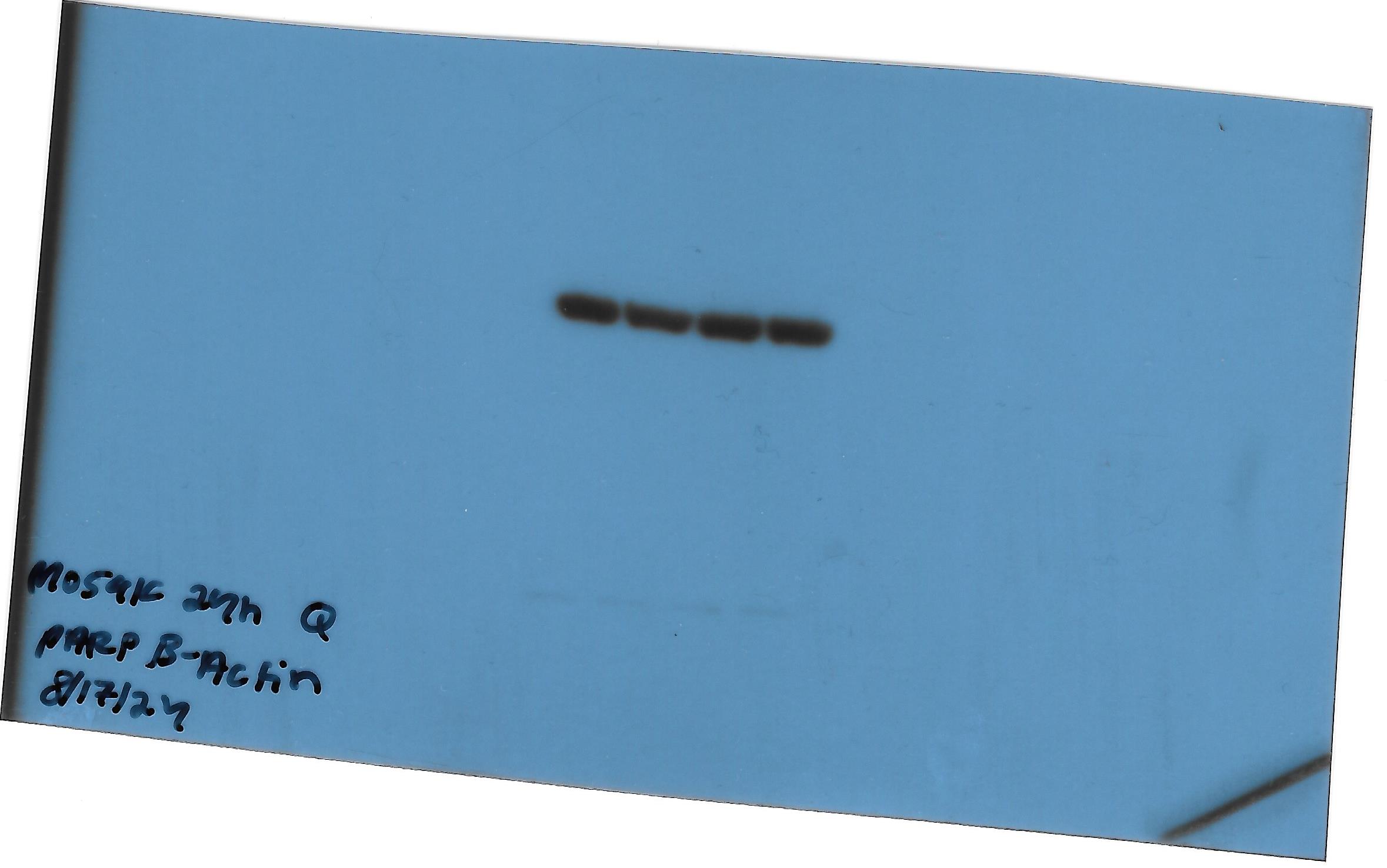

Supplement: Supplementary file 1 [file cancers-17-03197-s001.zip › OriginalBlots/FigureS1A-M059K-24h/2024-08-17_M059K_24h_Qonly_PARP_Actin_2.jpg]

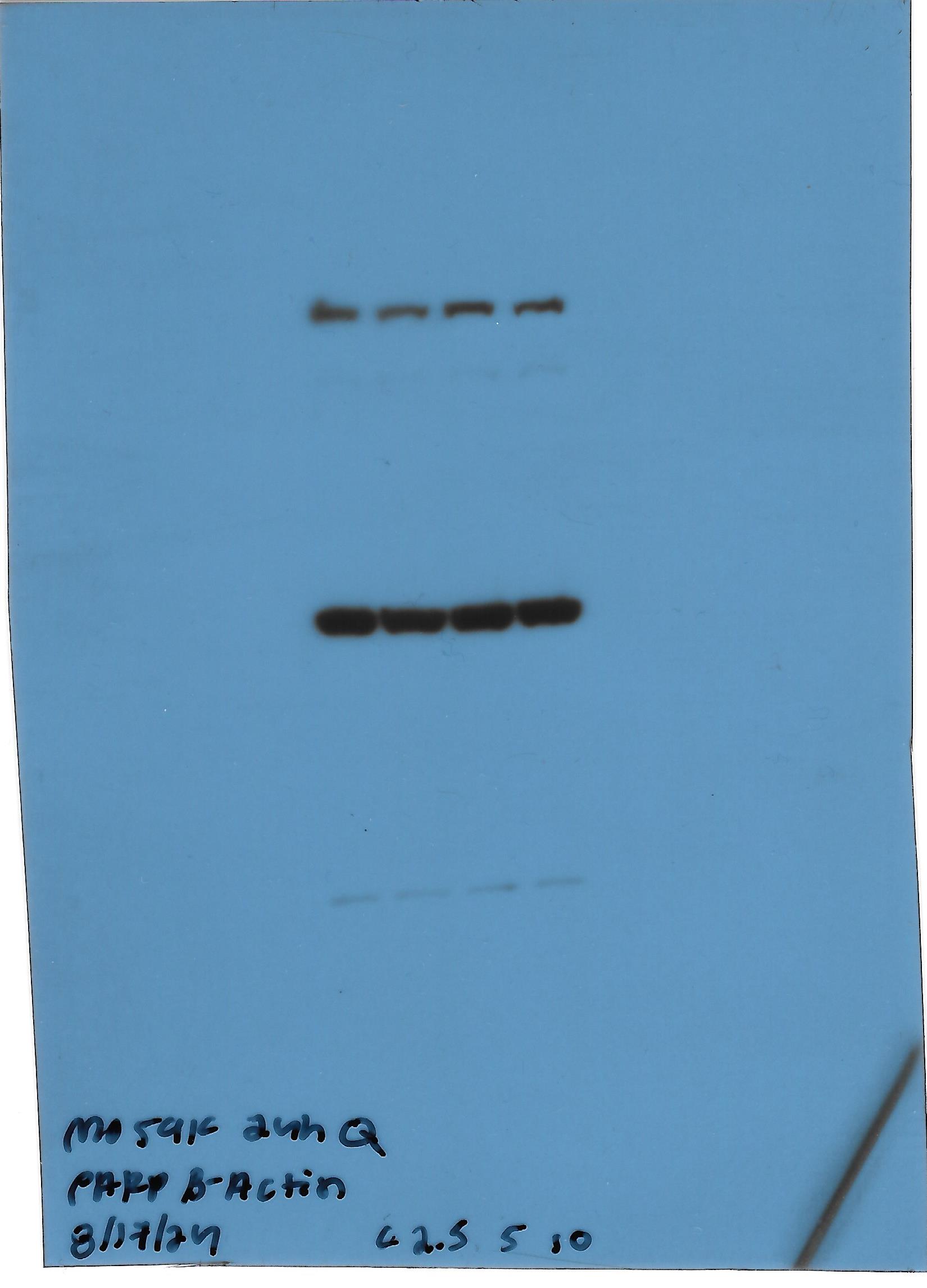

Supplement: Supplementary file 1 [file cancers-17-03197-s001.zip › OriginalBlots/FigureS1A-M059K-24h/2024-08-17_M059K_24h_Qonly_PARP_Actin_3.jpg]

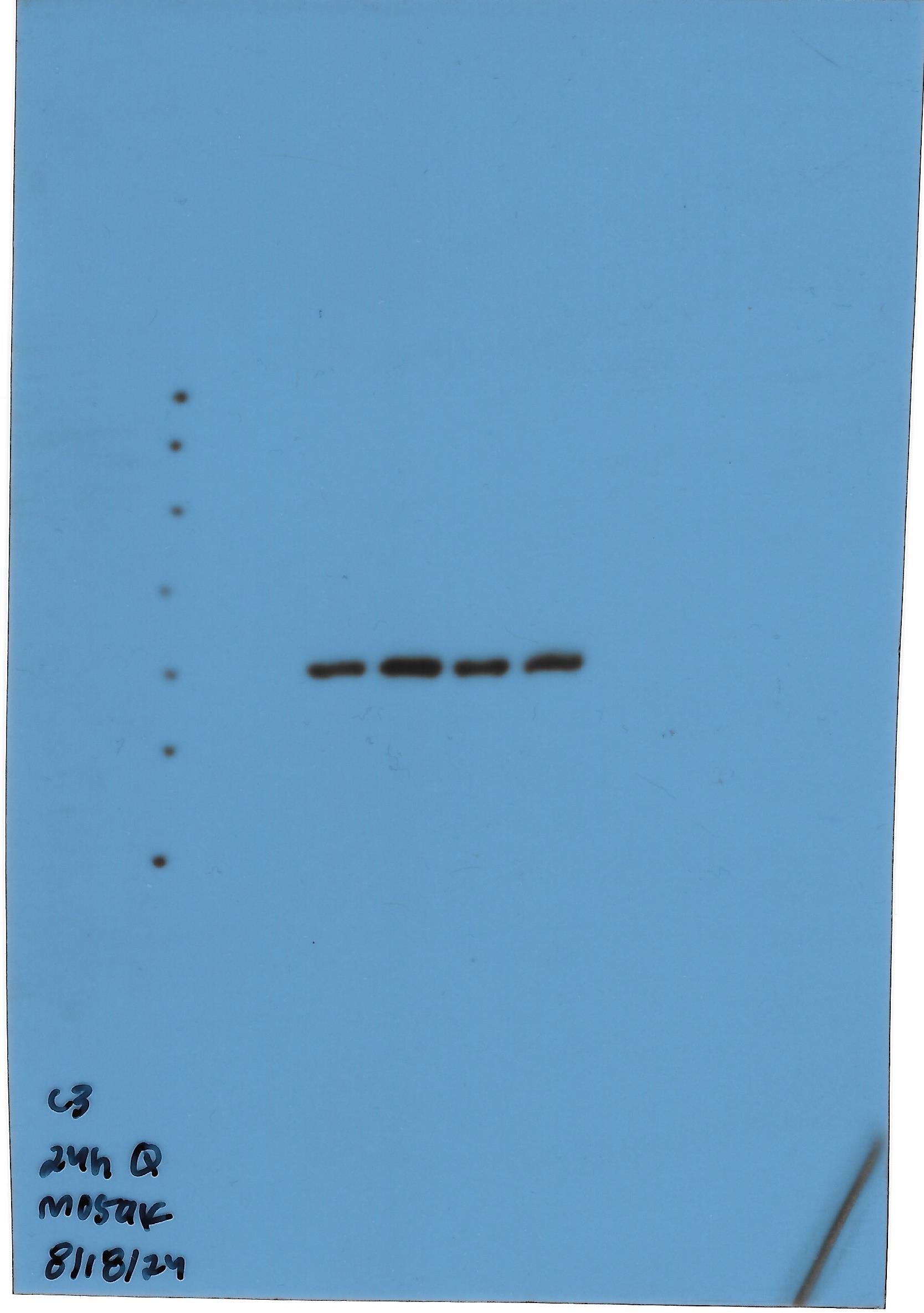

Supplement: Supplementary file 1 [file cancers-17-03197-s001.zip › OriginalBlots/FigureS1A-M059K-24h/2024-08-18_M059K_24h_Qonly_C3_2.jpg]

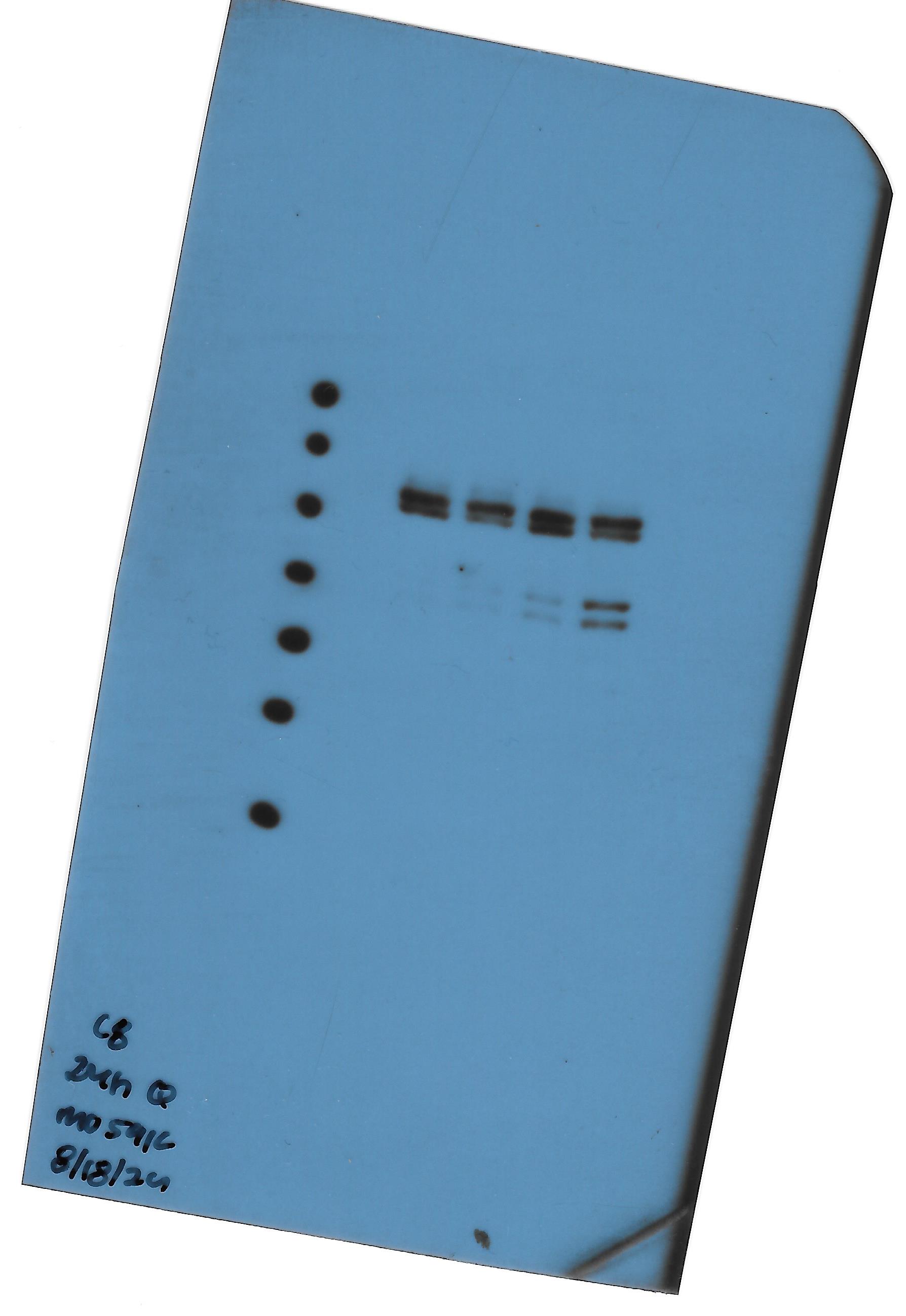

Supplement: Supplementary file 1 [file cancers-17-03197-s001.zip › OriginalBlots/FigureS1A-M059K-24h/2024-08-18_M059K_24h_Qonly_C8_2.jpg]

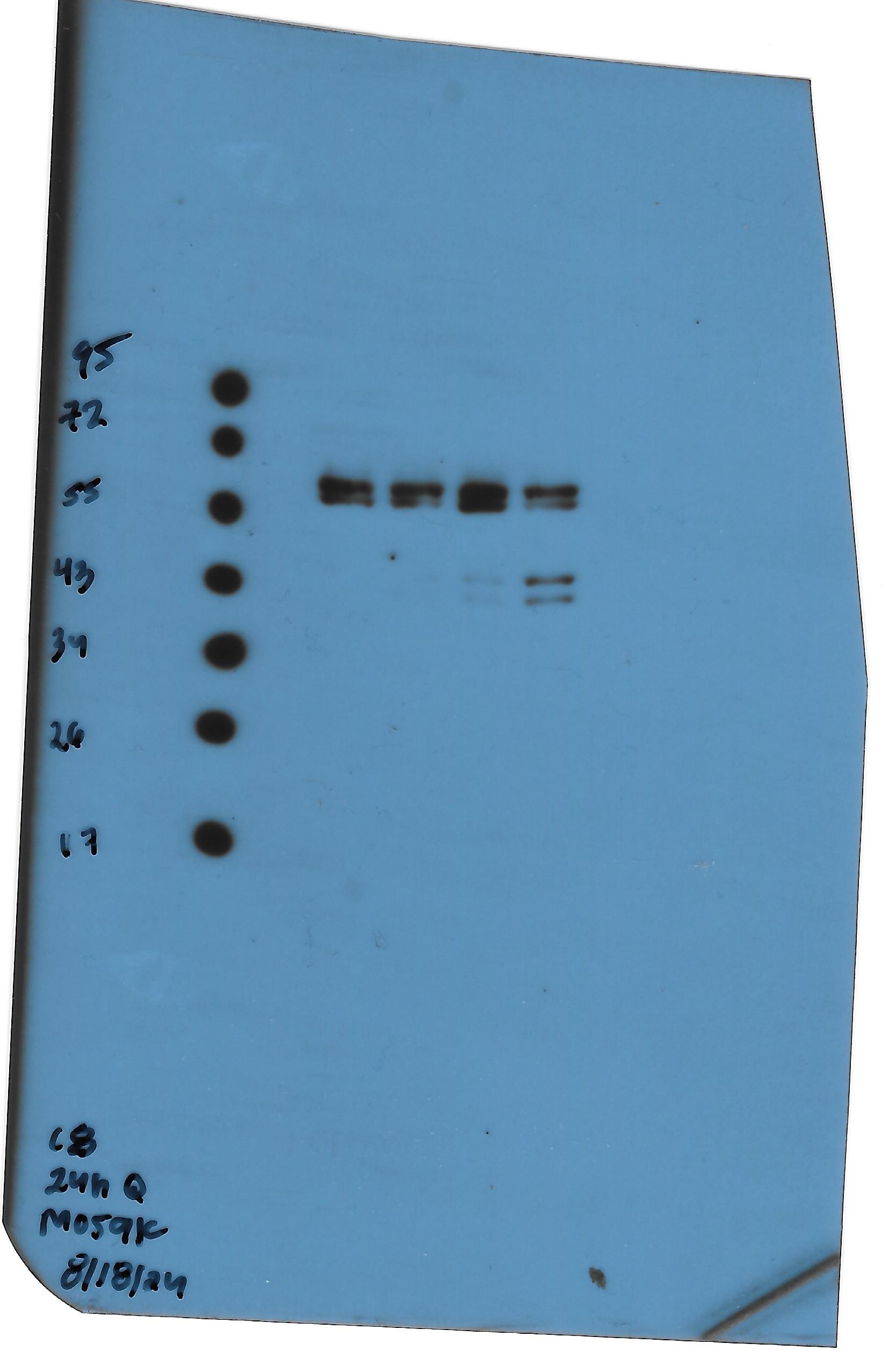

Supplement: Supplementary file 1 [file cancers-17-03197-s001.zip › OriginalBlots/FigureS1A-M059K-24h/2024-08-18_M059K_24h_Qonly_C8_5.jpg]

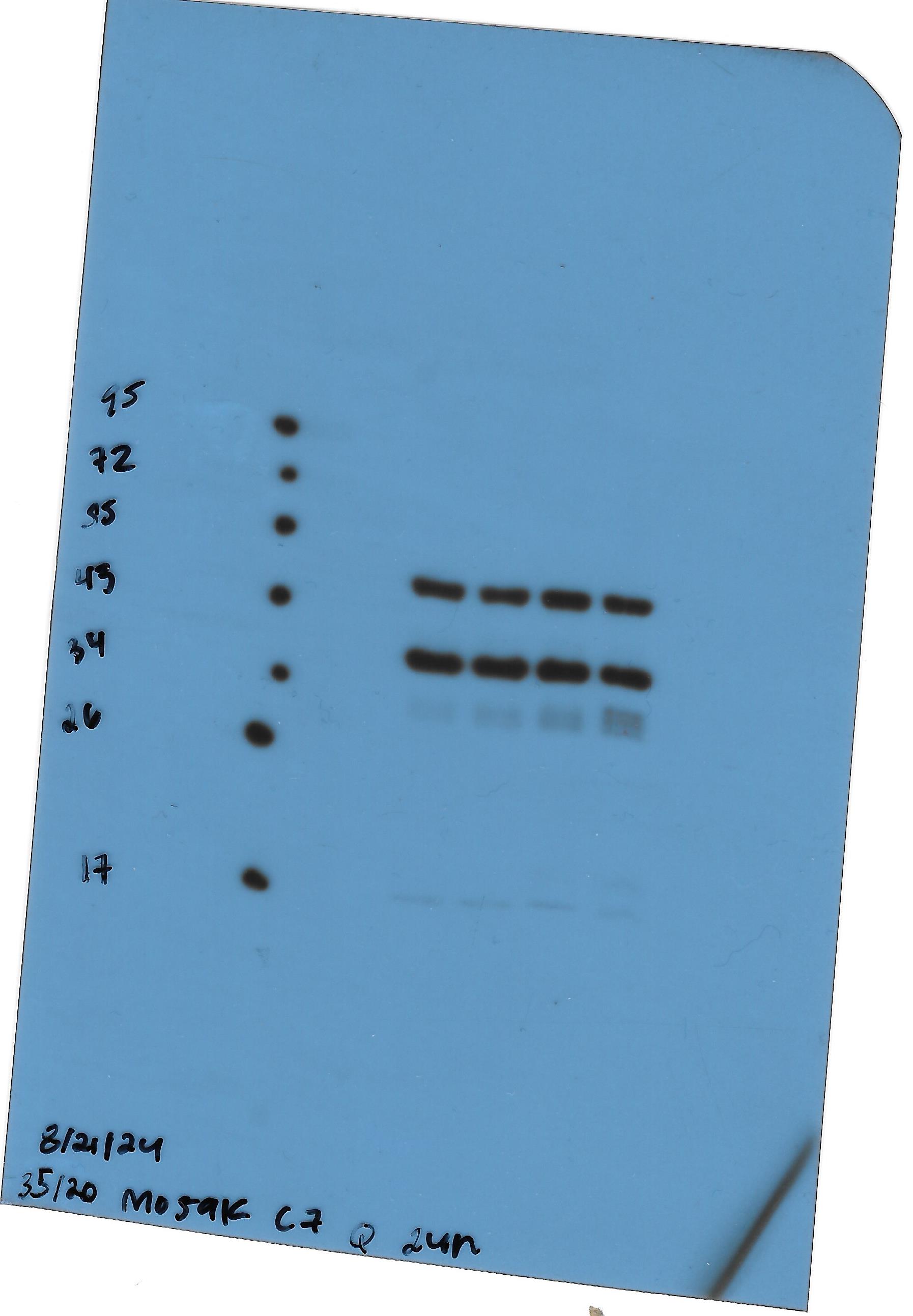

Supplement: Supplementary file 1 [file cancers-17-03197-s001.zip › OriginalBlots/FigureS1A-M059K-24h/2024-08-21_M059K_24h_Qonly_C7_1.jpg]

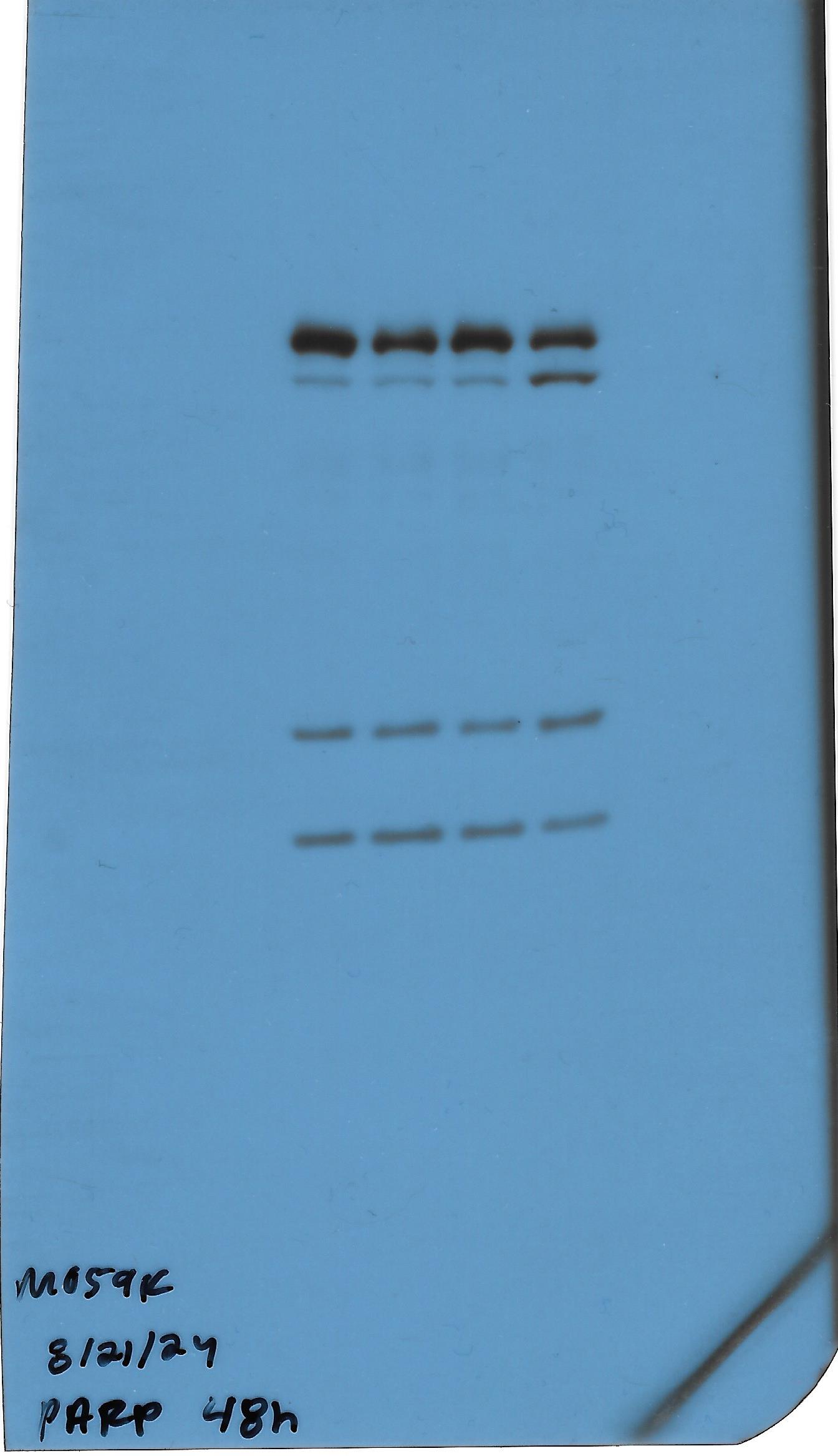

Supplement: Supplementary file 1 [file cancers-17-03197-s001.zip › OriginalBlots/FigureS1B-M059K-48h/2024-08-21_M059K_48h_Qonly_PARP_4.jpg]

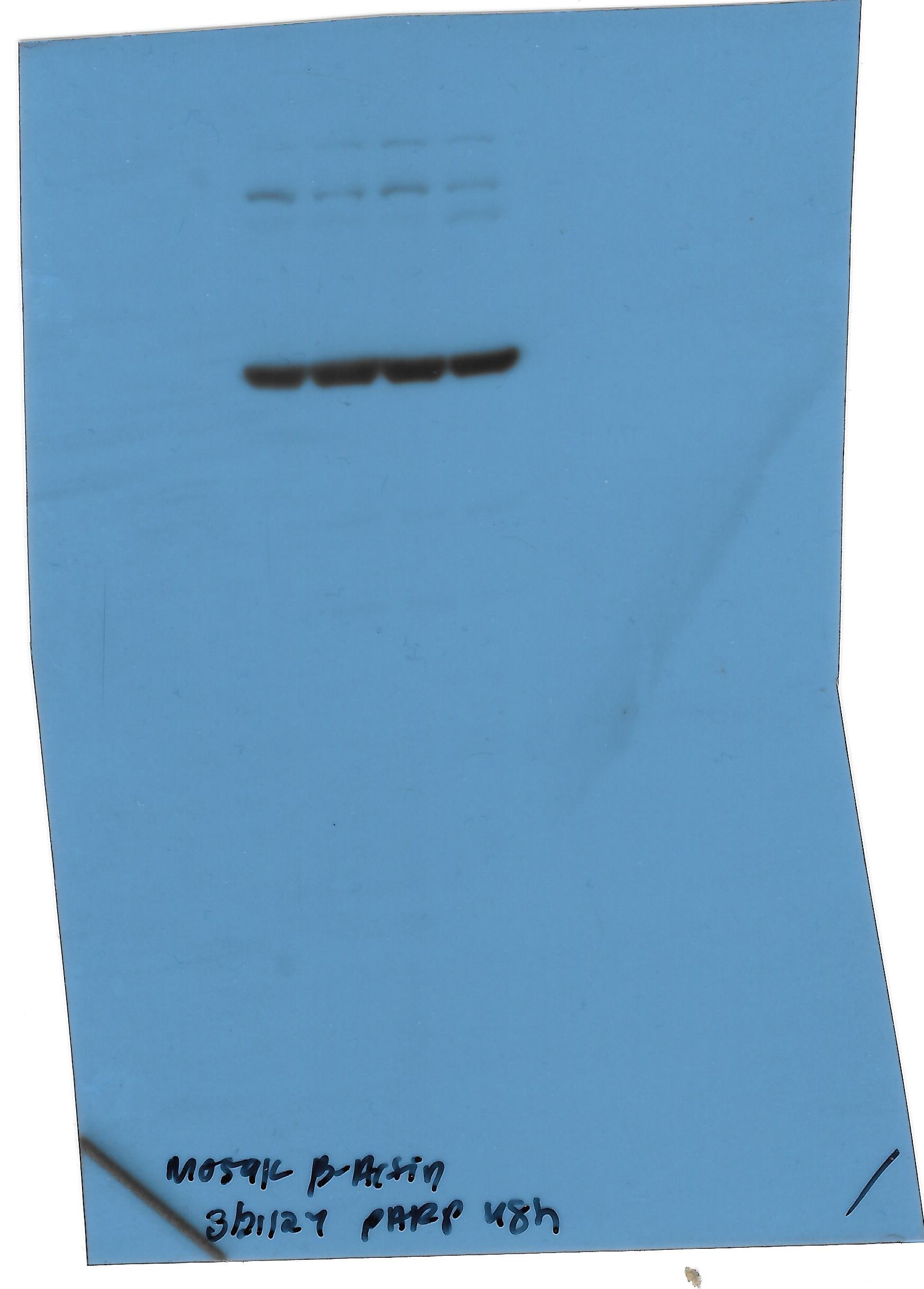

Supplement: Supplementary file 1 [file cancers-17-03197-s001.zip › OriginalBlots/FigureS1B-M059K-48h/2024-08-21_M059K_48h_Qonly_PARP_Actin_2.jpg]

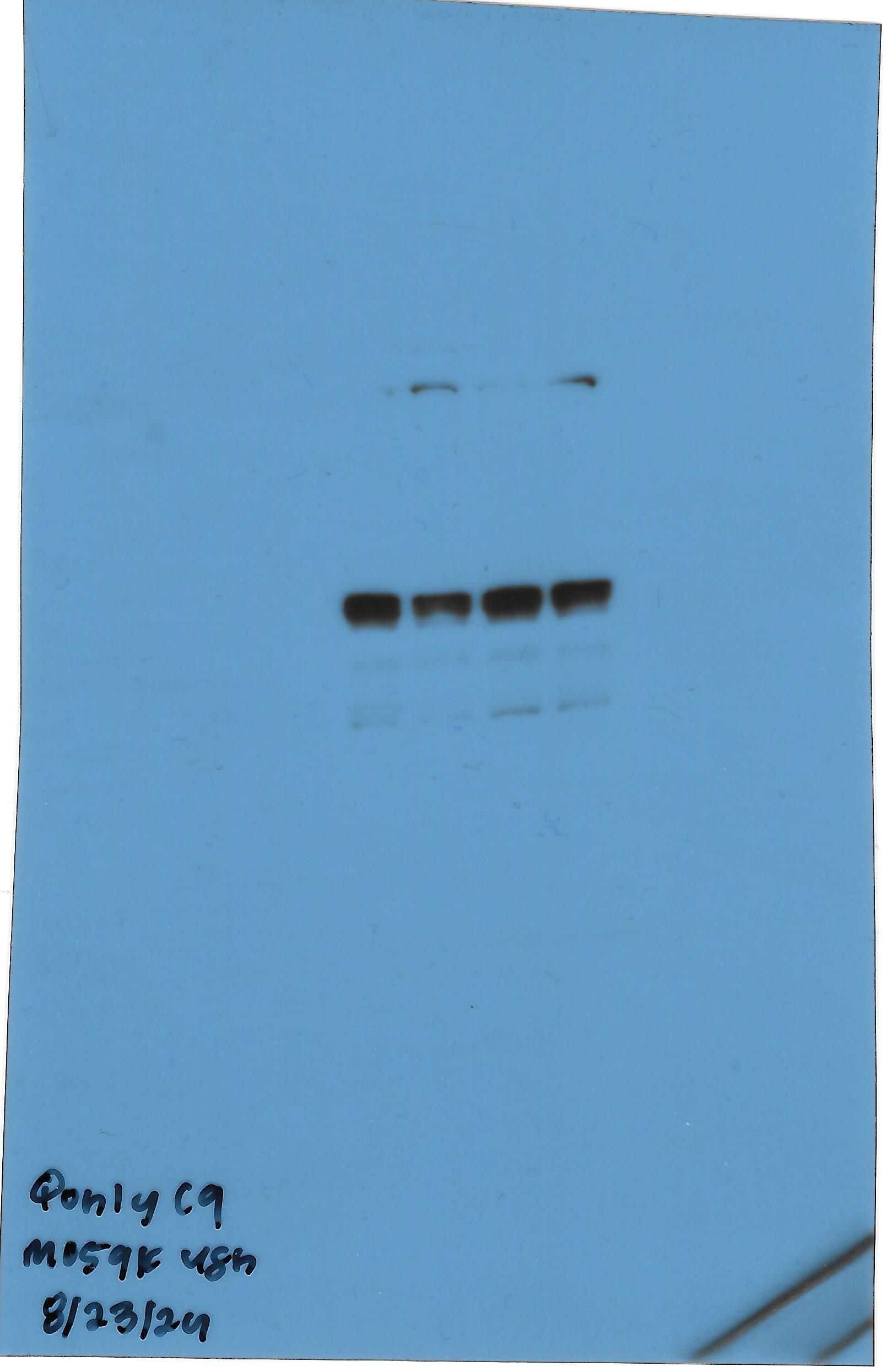

Supplement: Supplementary file 1 [file cancers-17-03197-s001.zip › OriginalBlots/FigureS1B-M059K-48h/2024-08-23_M059K_48h_Qonly_C9_1.jpg]

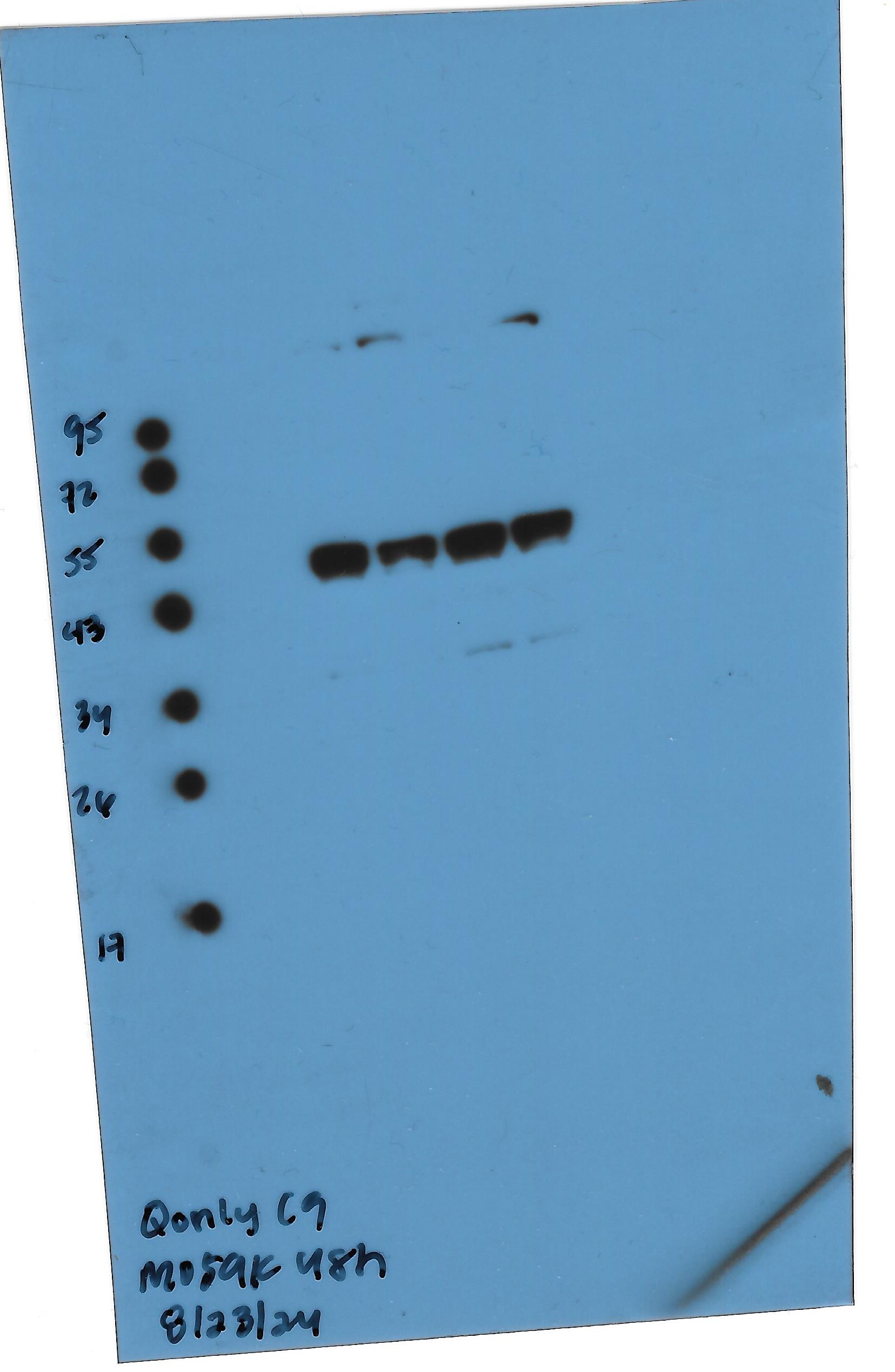

Supplement: Supplementary file 1 [file cancers-17-03197-s001.zip › OriginalBlots/FigureS1B-M059K-48h/2024-08-23_M059K_48h_Qonly_C9_2.jpg]

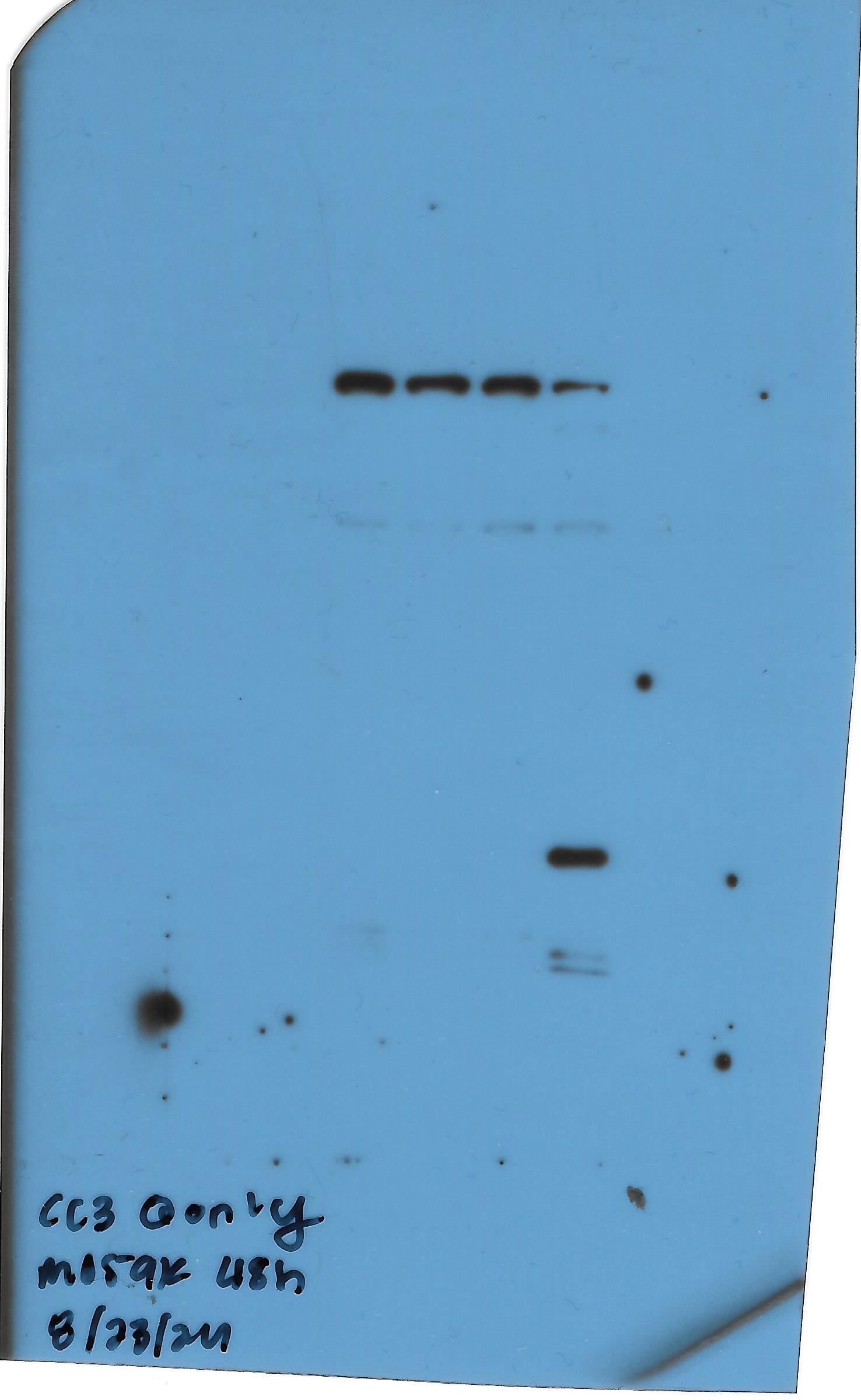

Supplement: Supplementary file 1 [file cancers-17-03197-s001.zip › OriginalBlots/FigureS1B-M059K-48h/2024-08-23_M059K_48h_Qonly_CC3_3.jpg]

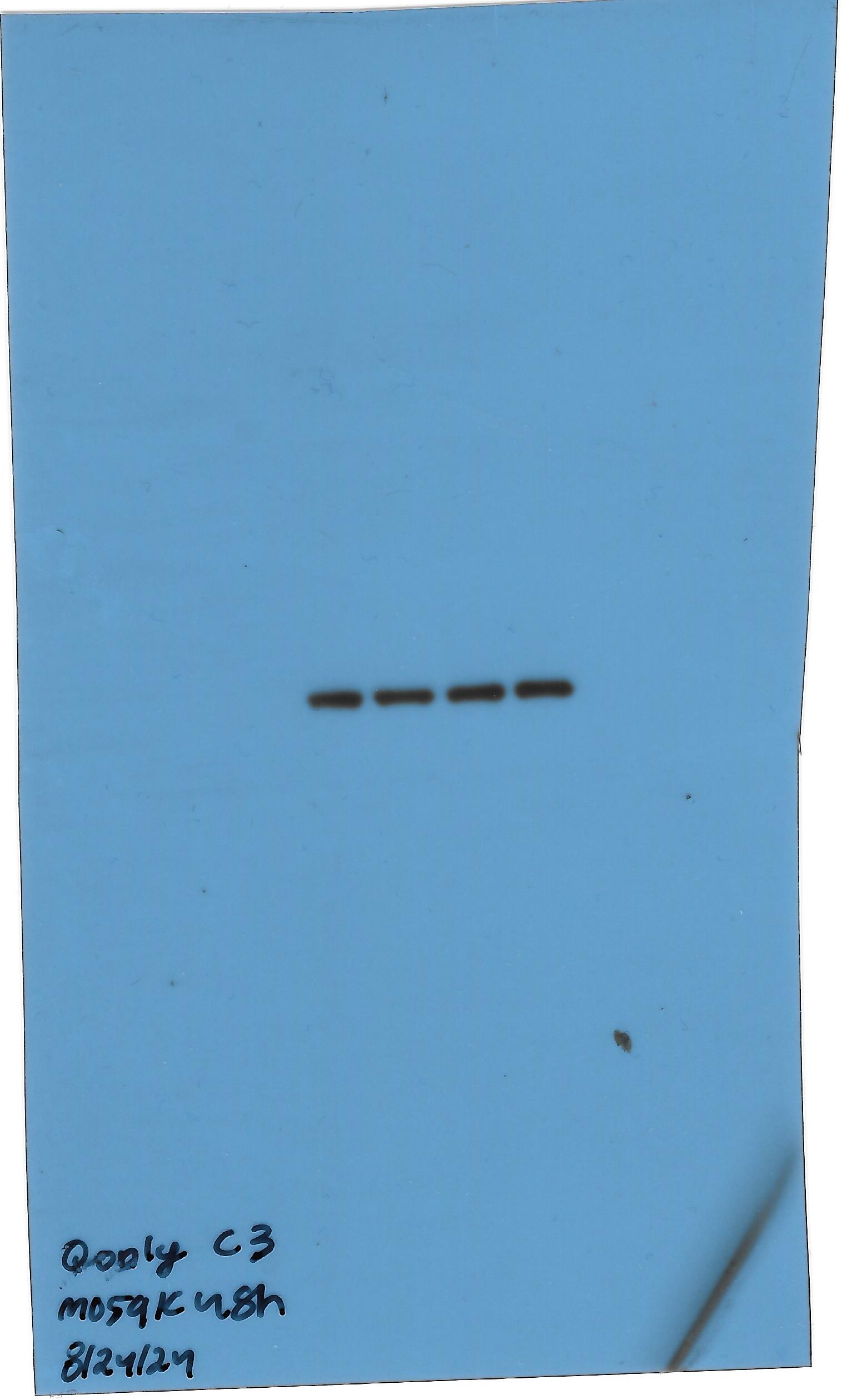

Supplement: Supplementary file 1 [file cancers-17-03197-s001.zip › OriginalBlots/FigureS1B-M059K-48h/2024-08-24_M059K_48h_Qonly_C3_3.jpg]

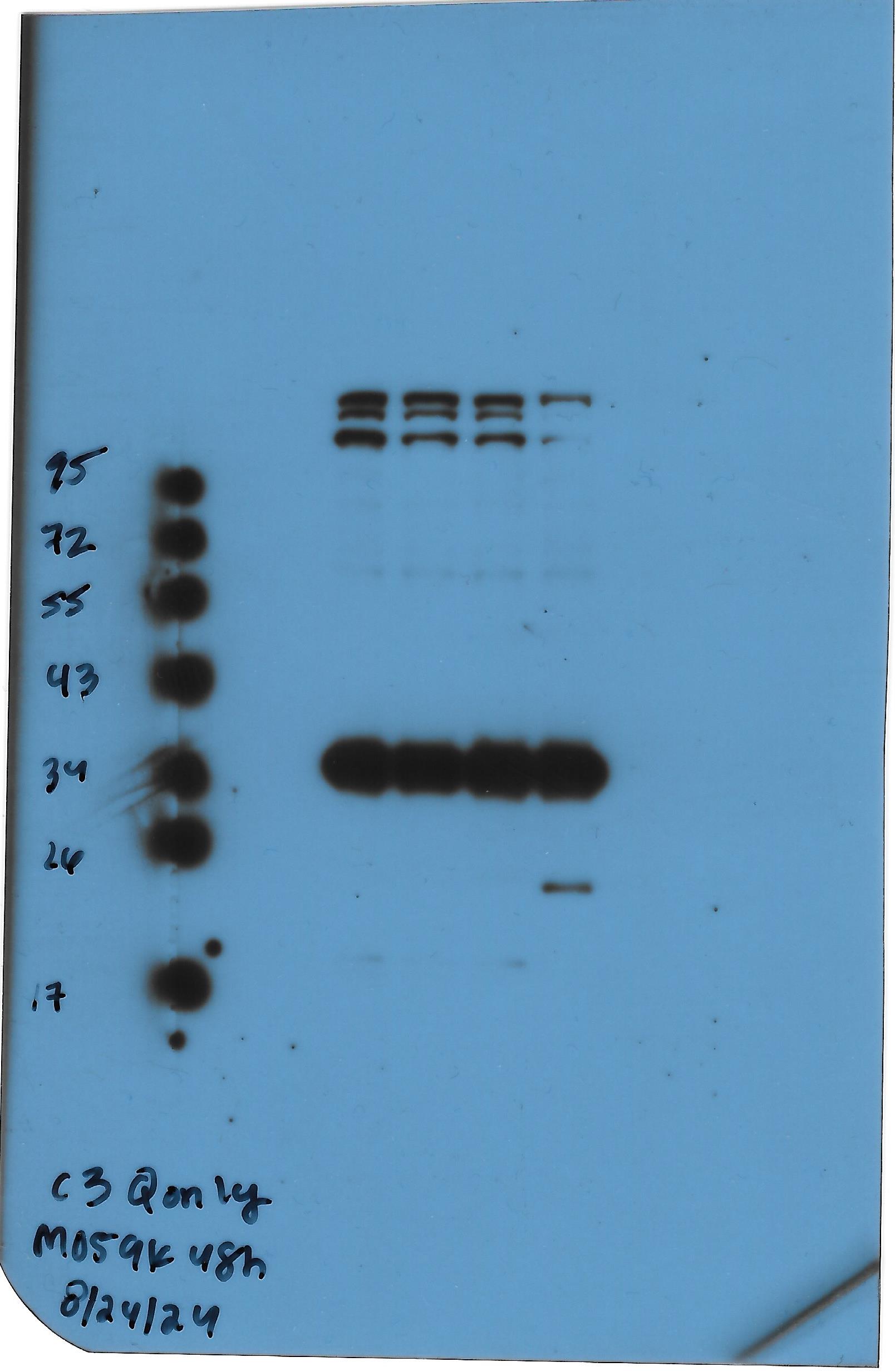

Supplement: Supplementary file 1 [file cancers-17-03197-s001.zip › OriginalBlots/FigureS1B-M059K-48h/2024-08-24_M059K_48h_Qonly_C3_5.jpg]

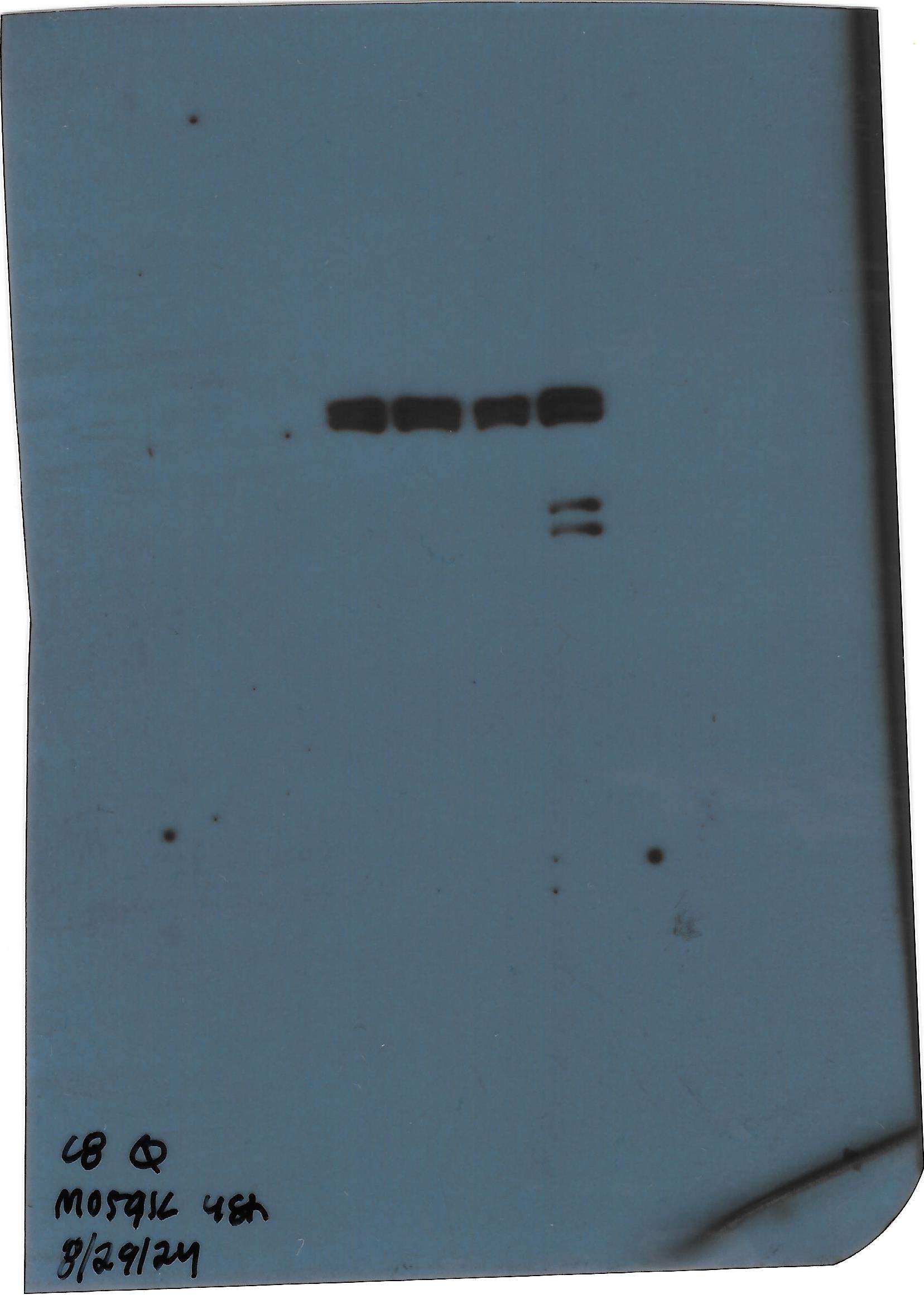

Supplement: Supplementary file 1 [file cancers-17-03197-s001.zip › OriginalBlots/FigureS1B-M059K-48h/2024-08-29_M059K_48h_Qonly_C8_2.jpg]
